# Progressive Reduction in Mitochondrial Mass Is Triggered by Alterations in Mitochondrial Biogenesis and Dynamics in Chronic Kidney Disease Induced by 5/6 Nephrectomy

**DOI:** 10.3390/biology10050349

**Published:** 2021-04-21

**Authors:** Rodrigo Prieto-Carrasco, Fernando E. García-Arroyo, Omar Emiliano Aparicio-Trejo, Pedro Rojas-Morales, Juan Carlos León-Contreras, Rogelio Hernández-Pando, Laura Gabriela Sánchez-Lozada, Edilia Tapia, José Pedraza-Chaverri

**Affiliations:** 1 Department of Biology, Faculty of Chemistry, National Autonomous University of Mexico (UNAM), Mexico City 04510, Mexico; rodrigo_prieto@my.uvm.edu.mx (R.P.-C.); svarta02@comunidad.unam.mx (O.E.A.-T.); pedrorojasm@comunidad.unam.mx (P.R.-M.); 2 Department of Cardio-Renal Pathophysiology, National Institute of Cardiology “Ignacio Chávez”, Mexico City 14080, Mexico; enrique.garcia@cardiologia.org.mx (F.E.G.-A.); laura.sanchez@cardiologia.org.mx (L.G.S.-L.); edilia.tapia@cardiologia.org.mx (E.T.); 3 Experimental Pathology Section, National Institute of Medical Sciences and Nutrition “Salvador Zubirán”, Mexico City 14000, Mexico; carlos.leonc@innsz.mx (J.C.L.-C.); rogelio.hernandezp@innsz.mx (R.H.-P.)

**Keywords:** chronic kidney disease, 5/6 nephrectomy, mitochondrial biogenesis, dynamics, mitochondrial impairment

## Abstract

**Simple Summary:**

In this work, we show for the first time a time-course study of the changes in mitochondrial biogenesis and dynamics markers in remnant renal mass from day 2 to day 28 after 5/6 nephrectomy. The present work shows a progressive reduction in mitochondrial biogenesis triggered by reducing two principal regulators of mitochondrial protein expression, the peroxisome proliferator-activated receptor-gamma coactivator 1-alpha (PGC-1α) and the peroxisome proliferator-activated receptor alfa (PPARα). Additionally, we found a slow and gradual change in mitochondrial dynamics from fusion to fission, favoring mitochondrial fragmentation at later stages after 5/6Nx. These changes involved in chronic kidney disease (CKD) development provide important advances in the molecular study of this disease that is a growing worldwide health problem.

**Abstract:**

The five-sixth nephrectomy (5/6Nx) model is widely used to study the mechanisms involved in chronic kidney disease (CKD) progression. Mitochondrial impairment is a critical mechanism that favors CKD progression. However, until now, there are no temporal studies of the change in mitochondrial biogenesis and dynamics that allow determining the role of these processes in mitochondrial impairment and renal damage progression in the 5/6Nx model. In this work, we determined the changes in mitochondrial biogenesis and dynamics markers in remnant renal mass from days 2 to 28 after 5/6Nx. Our results show a progressive reduction in mitochondrial biogenesis triggered by reducing two principal regulators of mitochondrial protein expression, the peroxisome proliferator-activated receptor-gamma coactivator 1-alpha and the peroxisome proliferator-activated receptor alpha. Furthermore, the reduction in mitochondrial biogenesis proteins strongly correlates with the increase in renal damage markers. Additionally, we found a slow and gradual change in mitochondrial dynamics from fusion to fission, favoring mitochondrial fragmentation at later stages after 5/6Nx. Together, our results suggest that 5/6Nx induces the progressive reduction in mitochondrial mass over time via the decrease in mitochondrial biogenesis factors and a slow shift from mitochondrial fission to fusion; both mechanisms favor CKD progression in the remnant renal mass.

## 1. Introduction

Renal function highly depends upon mitochondrion homeostasis; this organelle is the principal source of adenosine triphosphate (ATP) production in the kidney and is also involved in several cellular signaling processes [1,2,3]. Consequently, it is not surprising that this organelle has emerged in recent years as a critical element in the development of numerous kidney diseases [4,5,6,7], including chronic kidney disease (CKD), a renal pathology characterized by the progressive loss of renal function over time and progressive decrement in the glomerular filtration rate (GFR) [8,9]. Currently, CKD is a growing worldwide public health problem [10], and the current clinical strategies to prevent illness progression are not enough [11]. Therefore, it is necessary to explore and understand the mechanism involved in this pathology to develop new treatments able to mitigate this disease’s advancement.

Five-sixth nephrectomy (5/6Nx) is an experimental model widely used to study the mechanisms involved in CKD progression because renal ablation triggers progressive deterioration that emulates clinical CKD [12,13]. In this model, early after surgery (1 day), the adaptive changes in remnant mass such as hypermetabolic state, hemodynamic changes, and hypertrophy trigger the impairment of mitochondrial bioenergetics [5,13], characterized by a reduction in the oxidative phosphorylation capacity (OXPHOS) and mitochondrial uncoupling [14]. Furthermore, we previously demonstrated that the impairment of mitochondrial bioenergetics observed from day 1 persisted until day 28 [15]. Such an effect favors the decrease in mitochondrial β-oxidation and ATP production, favoring permanent oxidative stress in the mitochondria, triggering CKD development in the 5/6Nx model [15]. This mitochondrial impairment was first related to a reduction in the activity of the complexes of the electron transport system (ETS) [14,15]. However, the other processes that regulate mitochondrial dynamics and turnover (fission, fusion, biogenesis, and mitophagy) [16] could also be involved, especially later. However, until now, there are no temporal studies of the change in the mitochondrial biogenesis, dynamics, and mitophagy that allow determining the participation of this process in mitochondrial impairment in the 5/6Nx model. Hence, their participation in renal damage progression in this model is presently unclarified.

Accordingly, in this work, we determined the changes in mitochondrial biogenesis and dynamics markers in remnant renal mass from day 2 to day 28 after 5/6Nx. We observed alterations in the abovementioned markers which can trigger the accumulation of damaged mitochondria in renal remnant mass favoring CKD development in this model. Taking into account that mitochondria have emerged as a key factor in CKD development, we believe that the present work contributes with new and valuable information of the mechanisms involved in mitochondrial dysfunction in the 5/6Nx model.

## 2. Materials and Methods

### 2.1. Reagents

Bovine serum albumin (BSA), fat-free BSA, ethylene glycol-bis(2-aminoethylether)-*N*,*N*,*N*′,*N*′-tetraacetic acid (EGTA), 4-(2-hydroxyethyl)-1-piperazineethanesulfonic acid (HEPES), manganese(II) chloride (MgCl_2_) tetrahydrate, paraformaldehyde, sodium dodecyl sulfate (SDS), sucrose, cacodylate buffer, osmium tetraoxide, uranyl acetate, and lead citrate were purchased from Sigma-Aldrich (St. Louis, MO, USA). Calcium chloride (CaCl_2_), sodium bicarbonate (NaHCO₃), ethylenediaminetetraacetic acid disodium salt dihydrate (EDTA), sodium phosphate dibasic (Na_2_HPO_4_), and sodium phosphate monobasic (NaH_2_PO_4_) were purchased from JT Baker (Xalostoc, Edo. Mex., Mexico). Sodium pentobarbital (Sedalphorte^®^), used as a sedative, was from Salud y Bienestar Animal S.A. de C.V. (Mexico City, Mexico). The PageRuler^TM^ Prestained Protein Ladder (10 to 180 kDa) was purchased from ThermoFisher Scientific (Waltham, MA, USA).

### 2.2. Experimental Design

The experimental protocol was approved by the Institutional Animal Care and Use Committee (CICUAL) of the National Institute of Cardiology “Ignacio Chávez” (INC/CICUAL/014/2018) and was conducted according to Mexican Official Norm Guides for the use and care of laboratory animals (NOM-062-ZOO-1999) and the disposal of biological residues (NOM-087-SEMARNAT-SSA1-2002). Male Wistar rats with an initial body weight between 250 to 300 g were randomly separated into two groups: (1) sham-operated rats (sham) and (2) 5/6Nx. Five-sixths nephrectomy was performed using the protocol previously described [12,14]. The analysis was carried out on days 2, 4, 7, 14, and 28 after 5/6Nx or sham surgery, with *n* = 4 per group for each time point. The animals were housed in a temperature-controlled temperature (22–24 °C) with a 12 h/12 h light/dark cycle and maintained with water and food ad libitum. At the end of each time point, animals were anesthetized with sodium pentobarbital (120 mg/kg), and renal remnant mass was excised for further analysis.

### 2.3. Histologic Studies

Immediately after rats were euthanized, tissue slices of remnant kidney were fixed by immersion in 10% formaldehyde in phosphate-buffered saline (PBS). Tissue slices of 1 mm width were dehydrated and embedded in paraffin, sectioned at 5 μm, and stained with hematoxylin/eosin (H&E). For morphometric evaluation of tubular damage, the numbers of normal proximal convoluted tubules, injured tubules (necrotic and detached epithelium with hyaline cylinders), and atrophic tubules (flattened epithelium and wide tubular lumen) were counted in five randomly chosen fields at 200× magnification per kidney, and their percentages were determined. Three animals were studied per time point.

### 2.4. Electron Microscopy Study

For ultrastructural electron microscopy evaluation, small tissue fragments from the remnant kidney were fixed with 2.5% glutaraldehyde in 0.15 M cacodylate buffer, post-fixed with 1% osmium tetroxide, dehydrated with ethyl alcohol in ascending concentrations, and infiltrated in epoxy resin. Ultrathin sections, from 70–90 nm, were mounted on copper grids, contrasted with uranyl acetate and lead citrate, and subsequently observed with an electron microscope (Tecnai Spirit BioTwin, FEI, Hillsboro, OR, USA).

### 2.5. Renal Mitochondrial Isolation

Mitochondria were isolated from remnant kidneys as previously described [14,15]. Briefly, mitochondria were isolated by differential centrifugation in 4 °C isolation buffer (225 mM d-mannitol, 75 mM sucrose, 1 mM EDTA, 5 mM HEPES, and 0.1% BSA fatty acid- free, pH 7.4). The final mitochondria pellet was resuspended in 180 μL of BSA-free isolation buffer, and the total mitochondrial protein was measured using the Lowry method.

### 2.6. Protein Extraction

To obtain total protein from remnant kidney or isolated mitochondria, the samples were resuspended in radioimmunoprecipitation buffer (RIPA): 40 mM Tris-HCl, 150 mM NaCl, 2 mM EDTA, 10% glycerol, 1% Triton X-100, 0.5% sodium deoxycholate, and 0.2% SDS, pH 7.6. Then, the samples were incubated for 30 min at 48 °C and were sonicated three times for 30 s at low intensity in an ultrasonic processor. Homogenates were centrifuged at 14,000× *g* for 40 min at 4 °C, and the supernatants were collected. Proteins were denatured by boiling for 10 min and then diluted 1:5 in Laemmli buffer (60 mM Tris-Cl, pH = 6.8, 2% SDS, 10% glycerol, 5% β-mercaptoethanol, and 0.01% bromophenol blue).

### 2.7. Western Blot (WB) Analysis

First, 30 µg of protein obtained from remnant kidneys as previously described was separated by 10% SDS-polyacrylamide gel electrophoresis (PAGE), and electrophoresis was run (Mini Protean II, Bio-Rad, Hercules, CA, USA). Molecular weight standards were run in parallel. Proteins were transferred to polyvinylidene fluoride (PVDF) membranes. Nonspecific protein binding was blocked by incubation with 5% nonfat milk in PBS containing 0.4% Tween-20, for 1 h, at room temperature. Membranes were incubated overnight at 4 °C first with the appropriated primary antibodies and then were incubated with the corresponding secondary antibodies for 1 h. The following antibodies were from Abcam (Cambridge, UK): anti-carnitine palmitoyltransferase I (CPT1) (ab234111, 1:2500 dilution), anti-peroxisome proliferator-activated receptor gamma coactivator 1-alpha (PGC-1α) (ab3242, 1:5000 dilution), anti-peroxisome proliferator-activated receptor alpha (PPARα) (ab24509, 1:5000 dilution), anti-optic atrophy 1 (OPA1) (ab42364, 1:1000 dilution), anti-phosphate and tensin homolog (PTEN)-induced kinase 1 (PINK1) (ab216144, 1:1000 dilution), and anti-parkin (ab77924, 1:1000 dilution). Anti-guanine adenine (GA)-binding protein subunit beta-1 (GABP-β ½ or nuclear respiratory factor 2 (NRF2), sc-271571, 1:1000 dilution), anti- nuclear respiratory factor 1 (NRF1) (sc-33771, 1:2000 dilution), anti-neutrophil gelatinase-associated lipocalin (NGAL) (sc-515876, 1:10,000 dilution), anti-profibrotic molecule transforming growth factor beta 1 (TGF-β1) (sc-130348, 1:1000 dilution), anti-dynamin-1-like protein (DRP1) (sc-32898, 1:1000 dilution), anti-mitofusin-1 (MFN1) (sc-50330, 1:1000 dilution), and anti-mitochondrial fission 1 protein (FIS1) (sc-98900, 1:1000 dilution) were from Santa Cruz Biotechnology (Dallas, TX, USA). Anti-microtubule-associated proteins 1A/1B light chain 3B I and II (LC3B-I/II) (L7543-200UL, 1:2500 dilution), anti-ubiquitin-binding protein p62 (p62, P0067, 1:3000 dilution), and anti-voltage-dependent anion channel (VDAC) (V2139-200UL, 1:5000 dilution) were from Sigma Aldrich. Anti-kidney injury molecule-1 (KIM-1) (GTX85067, 1:3000 dilution), anti-mitochondrial transcription factor A (TFAM) (GTX112760, 1:4000 dilution), anti-alpha smooth muscle actin (α-SMA) (GTX100034, 1:4000 dilution), and anti-β-actin (GTX109639, 1:10,000 dilution) were from Genetex (Irvine, CA, USA). Anti-mitofusin-2 (MFN2) (94825) was from Cell Signaling (Danvers, MA, USA). Protein bands were detected by chemiluminescence that was visualized using horseradish peroxidase (HRP) secondary antibody (Cell Signaling, 7074) and the Immobilon Crescendo HRP substrate (WBLUR0100, Merck Millipore Burlington, MA, USA). To analyze immunoblots; Image Studio Lite 5.2, Licor Biosciences software was used. All the uncropped blots and the densitometric quantifications are shown in Figure S1 (Supplementary Materials).

### 2.8. Statistical Analysis

Data were presented as the mean ± standard error of the mean (SEM). They were analyzed by one-way analysis of variance with a subsequent Tukey test using the software Graph-Pad Prism 7 (San Diego, CA, USA). The level of significance was set at *p* < 0.05. Pearson’s correlation coefficient was used to determine the correlation of KIM-1, NGAL, TGF-β1, and α-SMA with PGC-1α, NRF1, or TFAM.

## 3. Results

### 3.1. Kidney Damage

**Figure 1:**
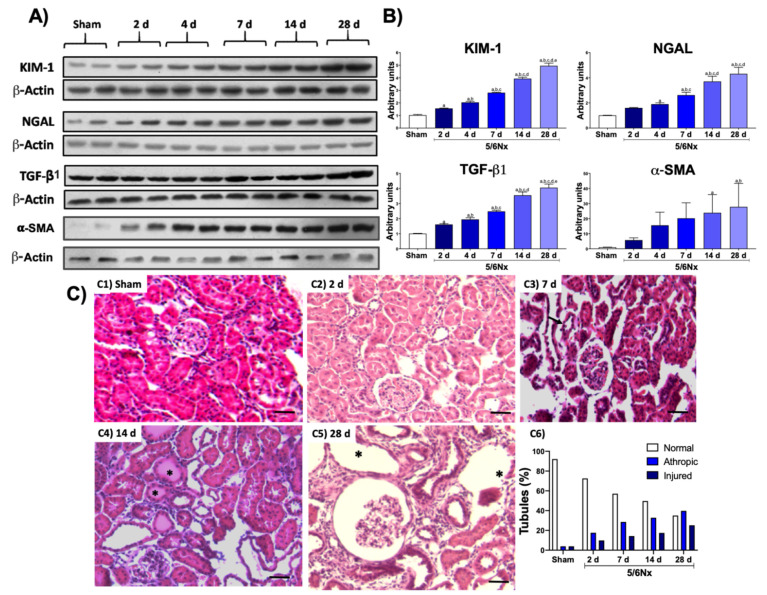
Temporal progression of kidney damage. (**A**) Representative Western blots of renal damage markers and fibrotic markers. (**B**) Quantifications of renal damage markers kidney injury molecule-1 (KIM-1) and neutrophil gelatinase-associated lipocalin (NGAL), as well as the profibrotic molecule transforming growth factor beta (TGF-β1) and alpha smooth muscle actin (α-SMA). β-Actin was used as loading control. Data are the mean ± SEM, *n* = 4. Tukey test. a = *p* ≤ 0.05 vs. sham, b = *p* ≤ 0.05 vs. 2 days, c = *p* ≤ 0.05 vs. 4 days, d = *p* ≤ 0.05 vs. 7 days, e = *p* ≤ 0.05 vs. 14 days, sham = simulated operation/control group. (**C**) Representative micrographs of kidney: (**C1**) Normal kidney histology from sham animal. (**C2**) After 2 days of 5/6 nephrectomy (5/6Nx), there were occasional cortical proximal tubules with flattened epithelium that correspond to tubular atrophy (arrows). (**C3**) After 1 week of nephrectomy, more tubules showed extensive epithelium damage (arrows). (**C4**) After 2 weeks of kidney resection, even more tubules show epithelial damage with hyaline casts in their lumen (asterisks). (**C5**) After 5/6Nx, there were numerous atrophic and dilated tubules (asterisks), as well as a glomerulus with mesangial hypercellularity surrounded by mild inflammatory infiltrate and fibrosis. (**C6**) The morphometric determination of tubular damage confirmed the progressive tubular damage when compared with the sham simulated/control group (S). (H/E staining, all micrographs 200× magnification, scale magnification bar = 50 µm).

The 5/6Nx model induces the increase in classic renal damage markers from the initial stages and the permanent alteration in structure and function of the remnant kidney [15,17]. To confirm the presence of renal damage, we evaluated the level of the proteins KIM-1 and NGAL, two renal damage markers associated principally with tubular injury, as well as the fibrotic markers TGF-β1 and α-SMA. KIM-1 and NGAL proteins showed a progressive increase in remnant kidney over time in the 5/6Nx group compared to the control group (Figure 1A,B), confirming tubular impairment progression. Moreover, TGF-β1 and α-SMA showed a progressive increase over time after day 2, indicating the development of fibrosis.

The histological evaluation using H&E-stained sections showed normal histology in sham animals (Figure 1C-C1). After two days of kidney resection, focal areas were randomly distributed of proximal convoluted tubules characterized by flattened atrophic epithelium (Figure 1C-C2); some tubules revisited with necrotic cells and hyaline casts in their lumens, surrounded by a slight inflammatory infiltrate and edema, were seen after 1 (Figure 1C-C3) and 2 (Figure 1C-C4) weeks of nephrectomy. These focal areas of tubular atrophy increased progressively in size and number. Thus, at day 28 after kidney resection, there were extensive areas of epithelial tubular atrophy and chronic inflammation with scare fibrosis (Figure 1C-C5); moderately sized arteries and arterioles exhibited thickening of the muscular wall with reduction of their lumens and glomeruli with an increase in the mesangial matrix, as well as cellularity and mild fibrosis. Morphometry determination of tubular damage confirmed these features, showing progressive tubular damage (Figure 1C-C6).

### 3.2. 5/6Nx Induces the Progressive Decrease in Mitochondrial Biogenesis Related to Tubular Damage and Fibrosis

**Figure 2:**
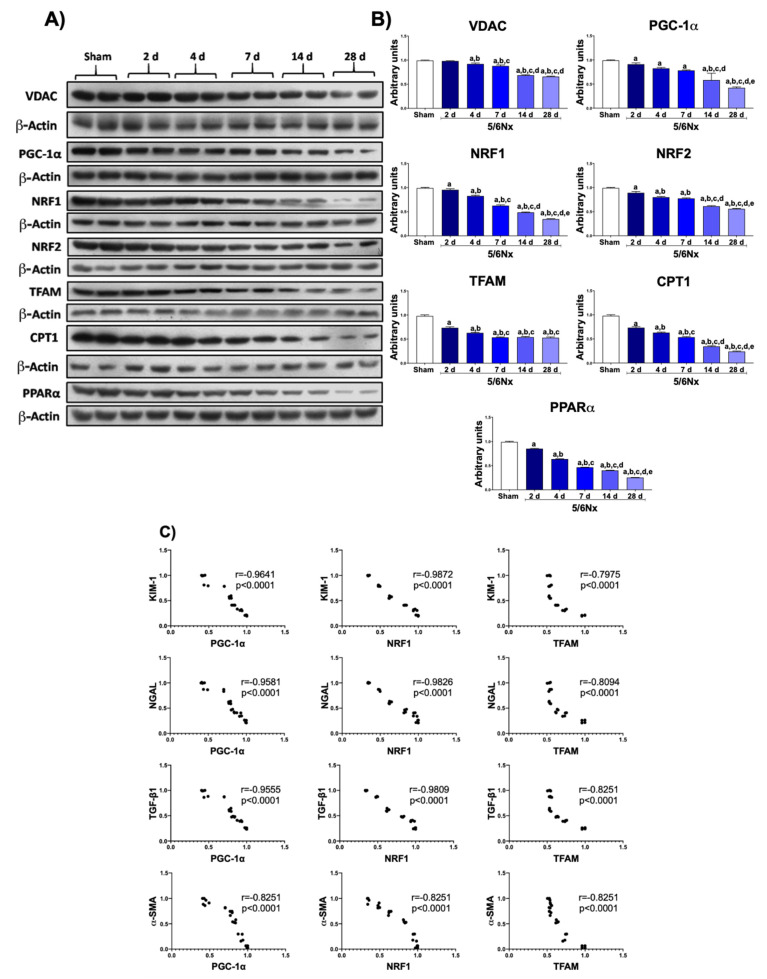
Progressive decrease in mitochondrial biogenesis in remnant renal mass. (**A**) Western blots and (**B**) quantifications of mitochondrial proteins: voltage-dependent anion channel (VDAC), peroxisome proliferator-activated receptor gamma coactivator 1-alpha (PGC-1α), nuclear respiratory factor 1 (NRF1) and 2 (NRF2), mitochondrial transcription factor A (TFAM), carnitine palmitoyltransferase 1 (CPT1), and peroxisome proliferator-activated receptor alpha (PPARα). β-Actin was used as loading control. (**C**) Correlation analyses of kidney injury molecule-1 (KIM-1), neutrophil gelatinase-associated lipocalin (NGAL), profibrotic molecule transforming growth factor beta 1 (TGF-β1), and alpha smooth muscle actin (α-SMA) with PGC-1α, NRF1, or TFAM. Data are the mean ± SEM, *n* = 4. Tukey test. a = *p* ≤ 0.05 vs sham, b = *p* ≤ 0.05 vs. 2 days, c = *p* ≤ 0.05 vs. 4 days, d = *p* ≤ 0.05 vs. 7 days, e = *p* ≤ 0.05 vs. 14 days, 5/6Nx = 5/6 nephrectomy, d = days after 5/6Nx, sham = simulated operation/control group.

To determine if the reduction in mitochondrial ATP production along the CKD transition [14,15] was related to mitochondrial content changes, we determined VDAC in remnant kidney at the various stages of progression. As shown in Figure 2A, VDAC progressively decreased over time in the 5/6Nx group, suggesting a reduction in mitochondrial mass in remnant kidney. Furthermore, the temporal evaluation of the primary regulator of mitochondria biogenesis, the PGC-1α protein, also showed a progressive decrease over time (Figure 2B), triggering a reduction in its downstream factors NRF1 and NRF2, as well as in TFAM, the principal transcriptional factor of mitochondrial deoxyribonucleic acid (mtDNA). These results strongly suggested that the impairment of mitochondrial biogenesis triggers a progressive reduction in mitochondrial mass in 5/6Nx. Furthermore, the levels of CPT1 (Figure 2A) and PPARα (Figure 2B), the principal metabolic regulators of fatty-acid metabolism, also progressively decreased over time, implying a progressive impairment of mitochondria β-oxidation by the reduction in mitochondrial biogenesis.

To evaluate if the progressive reduction in mitochondrial biogenesis markers was related to tubular damage (the nephron sections with a higher mitochondrial density), we performed a statistical correlation analysis of KIM-1, NGAL, TGF-β1, and α-SMA with PGC-1α, NRF1, or TFAM levels. Interestingly, the reduction in PGC-1α and NRF1 levels showed a strong correlation with the increase in tubular damage markers (Figure 2C). Furthermore, the increase in the fibrotic markers TGF-β1 and α-SMA also negatively correlated with the levels of mitochondrial biogenesis markers (Figure 2C). These results suggested that the reduction in mitochondrial content by impaired mitochondrial biogenesis promotes the tubular damage and the fibrotic processes in this model.

### 3.3. 5/6Nx Induces the Increase in Mitochondrial Fission and the Reduction in Mitochondrial Fusion

**Figure 3:**
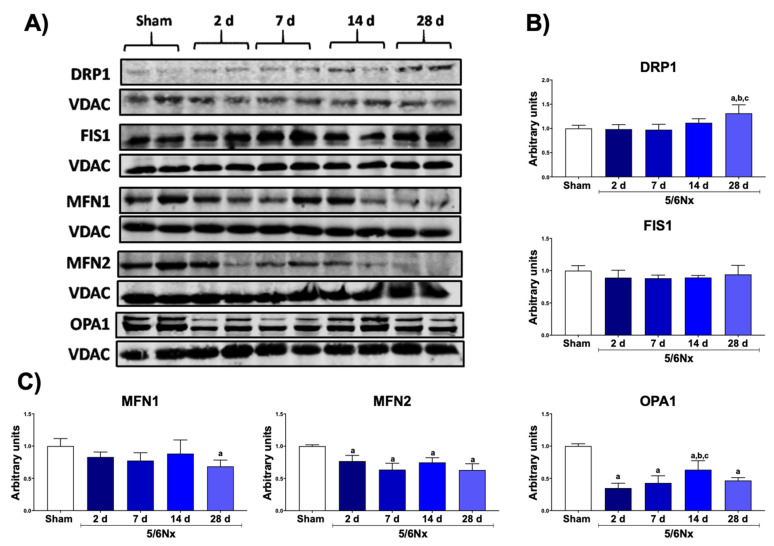
Alteration in mitochondrial dynamics in remnant renal mass. (**A**) Representative Western blots of the fission and fusion proteins. (**B**) Quantification of the fission proteins: dynamin-1-like protein (DRP1) and mitochondrial fission 1 protein (FIS1). (**C**) Quantification of the fusion proteins: mitofusin 1 (MFN1) and 2 (MFN2) and optic atrophy 1 (OPA1). Voltage-dependent anion channel (VDAC) was used as loading control. Data are the mean ± SEM, *n* = 4. Tukey test. a = *p* ≤ 0.05 vs. sham, b = *p* ≤ 0.05 vs. 2 days, c = *p* ≤ 0.05 vs. 4 days, 5/6Nx = 5/6 nephrectomy, d = days after 5/6Nx, sham = simulated operation/control group.

**Figure 4:**
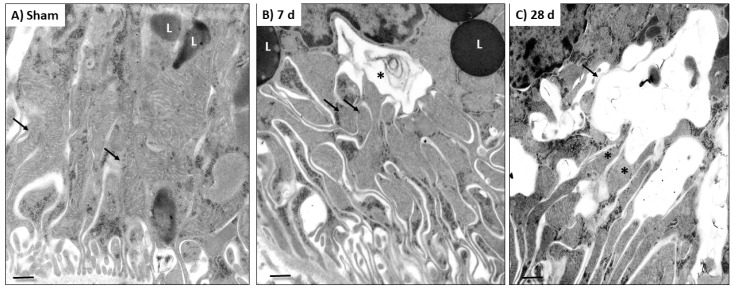
Representative ultrastructural micrographs of epithelial cells from proximal convoluted tubules in (**A**) sham animals and in (**B**,**C**) rats with 5/6 nephrectomy (5/6Nx). (**A**) Normal appearance of the mild and basal cytoplasmic areas of an epithelial tubular cell from a sham animal, showing long mitochondria (arrows) and round electron dense lysosomes (L). (**B**) After 7 days (d) of 5/6Nx, there were small and round mitochondria (arrows) and large cytoplasmic vacuoles limited by a double membrane corresponding to autophagosomes (asterisk), which were near to mitochondria and large lysosomes (L). (**C**) After 28 days of kidney resection, these cytoplasmic double-membrane vacuoles (arrow) were larger and directly connected to mitochondria (asterisks). Scale magnification bar = 500 nm.

The mitochondrion is a dynamic organelle whose distribution, quality control, and mass depend on the balance between the processes of fusion (merger) and fission (fragmentation) [18]. In fact, in many renal damage models, the impairment of mitochondrial ATP production has been linked with an imbalance in mitochondrial dynamics [5,16,18,19,20,21]. We previously showed that, 24 h after 5/6Nx, there is an increase in the localization of fusion proteins MFN1 and OPA1 in mitochondria [14], together with an increase in mitochondria mass in the proximal tubular section [14,22], suggesting a shift to mitochondrial fusion at early times after 5/6Nx. However, the changes in mitochondrial dynamics along the CKD transition remained poorly understood. Therefore, we evaluated the changes in mitochondrial dynamics proteins over time. Interestingly, DRP1 showed an increase only at 28 days after surgery, without significative changes in FIS1 levels (Figure 3A,B). On the other hand, fusion proteins MFN2 and OPA1 decreased in the 5/6Nx group, from day 2 until the end of the follow-up (Figure 3A,B). The ultrastructural analysis showed normal mitochondria of the sham group (Figure 4A); in contrast, there was a progressive increase in small and round mitochondria, suggesting a shift to mitochondrial fission (Figure 4B), after day 7 of 5/6Nx. Therefore, these results imply that the shift of mitochondrial dynamics to fission is a later process during CKD progression.

### 3.4. 5/6Nx Induces Temporal Alterations in Autophagy in the Remnant Kidney

**Figure 5:**
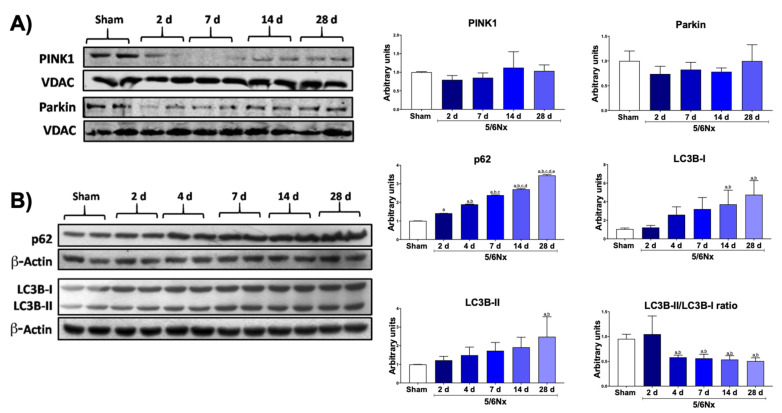
Alteration in mitophagy and autophagy markers in remnant mass. (**A**) Western blots and their quantifications of mitophagy proteins: phosphate and tensin homolog (PTEN)-induced kinase 1 (PINK1) and PARKIN in isolated mitochondria. Voltage-dependent anion channel (VDAC) was used as loading control. (**B**) Western blots and their quantifications of autophagy proteins: p62 and microtubule-associated proteins 1A/1B light chain 3B I and II (LC3B-I/II). β-Actin was used as loading control. Data are the mean ± SEM, *n* = 4. Tukey test. a = *p* ≤ 0.05 vs. sham, b = *p* ≤ 0.05 vs. 2 days, c = *p* ≤ 0.05 vs. 4 days, d = *p* ≤ 0.05 vs. 7 days, e = *p* ≤ 0.05 vs. 14 days, 5/6Nx = 5/6 nephrectomy, d = days after 5/6Nx, sham = simulated operation/control group.

The reduction in mitochondrial biogenesis can be also related to the removal of damaged mitochondria by the mitophagy process. Moreover, the ultrastructural study of the kidney section showed a progressive increase in the number of small and round mitochondria after day 7 (Figure 4B), whereby some of them were included or in direct contact with large double-membrane vacuoles that corresponded to autophagosomes (mitophagy), especially at 28 days (Figure 4C). Therefore, these results imply that mitochondrial degradation by autophagy is induced at later times after nephrectomy. Thus, we proceeded to evaluate the changes over time in the levels of mitophagy proteins PINK and Parkin, two proteins of the classical mitophagy pathway. Although WB images suggested that both proteins were decreased in 5/6Nx compared to sham, this reduction was not significant at any evaluated time point (Figure 5A). However, macroautophagy proteins p62 and LC3B-I/II showed a temporal increase in 5/6Nx (Figure 5B), suggesting autophagy induction. Congruently, the reduction in LC3B-II/LC3B-I ratio (Figure 5B) also suggests an increase in the induction of autophagy flux. Together with the observed increase in autophagosomes bodies at later times (Figure 4C), these findings suggest impairment in autophagy flux.

## 4. Discussion

Renal mass reduction in 5/6Nx triggers several adaptations in remnant nephrons to maintain kidney function [13]. Among them, the increases in single-nephron GFR, in solute reabsorption, and in the biosynthetic processes related to hypertrophy generate an increase in ATP demand, especially in tubular segments [23,24]. However mitochondrial adaptations after 5/6Nx are not enough to maintain the ATP supply in energetic stress conditions [13,25,26]. Such an unfavorable energy environment leads to the impairment in mitochondria, characterized by a reduction in OXPHOS capacity, by a decrease in respiratory complex I (CI) activity at the early stage [14], and by a decrease in respiratory complex III (CIII) activity at the later stage, together with mitochondrial β-oxidation reduction, especially in the time interval between 2 days and 28 days after subtotal renal ablation [15]. However, at the earliest time point (1 day) after 5/6Nx, these mitochondrial alterations are not linked to mitochondrial protein levels [15]. Nevertheless, at more advanced times of the disease, the participation of the processes that coordinate mitochondrial turnover (mitochondrial biogenesis and mitochondrial removal) cannot be excluded, especially because mitochondrial bioenergetics is tightly coordinated with mitochondrial turnover and dynamics, favoring specific changes in these processes depending of the energy demands [27,28,29]. However, the development of mitochondrial biogenesis and dynamics changes is unclear in the time interval between day 2 and day 28 following subtotal renal ablation [5].

Our results showed that, in this interval, there is a progressive reduction in mitochondrial biogenesis genes in the remnant kidney (Figure 2). In fact, in addition to mitochondrial bioenergetics alterations, there has been reported an early increase in mitochondrial volume per cell starting from day 1 and persisting until day 14 [14,22], without an increase in mtDNA or in the expression of nuclear encoded mitochondrial genes (with exception of glutathione transporters) [14,22,30,31]. This is congruent with our results, in which we observed a reduction in PGC-1α, NRF1, NRF2, and TFAM levels (Figure 2B), implying the downregulation of mitochondrial biogenesis machinery over time. This reduction appeared after day 2 and was progressive over time (Figure 2B), triggering a decrease in VDAC proteins from day 4 to day 28 (Figure 2A). In agreement with these data, proteomic analyses of the renal cortex of 5/6Nx rats at day 28 showed a downregulation of mitochondrial proteins: medium chain acetyl dehydrogenase (MCAD), phosphoglycerate kinase 1 (PGK-1), glucose-regulated protein-75 (GRP-75), nicotinamide adenine dinucleotide (NADH) dehydrogenase-ubiquinone 1 beta subcomplex subunit 8 (NDUFB8), and cytochrome c oxidase subunits I (COXI) and IV (COXIV) [32]. Furthermore, lower levels of ATP synthase β subunit, COXI, and NDUFB8 were also detected at 8 weeks after surgery in 5/6Nx rats [33]. Others also reported that a reduction in mitochondrial biogenesis appears after the impairment of bioenergetics [14]. However, the reduction in mitochondrial biogenesis starts from day 2, leading to a subsequent reduction in mitochondrial proteins of OXPHOS and β-oxidation at 4 weeks after surgery [32,33]. We previously reported that mitochondrial β-oxidation was impaired in the remnant kidney as early as day 2 with a reduction in ATP production [15]; this observation is in agreement with a reduction in CPT1 (Figure 2A) and PPARα (Figure 2B) levels, reported in the present study.

Some authors showed that, 12–13 weeks after 5/6Nx, there is a significant decrease in mtDNA in the remnant kidney, associated with impaired biogenesis induced by the increase in TGF-β1 level [34,35]. This is particularly interesting because the reduction in the expression of fatty-acid β-oxidation genes preceded the increase in fibrotic processes in other CKD models [36,37]. In fact, it was recently demonstrated that the reduction in fatty-acid β-oxidation proteins alone is enough to reprogram tubular epithelial cells into a profibrotic phenotype via the TGF-β1 pathway [36]. In agreement, our results showed a positive correlation between the reduction in NRF1 and PGC-1α (Figure 2B) and an increase in tubular damage proteins KIM-1 and NGAL (Figure 1A,B), implying that a reduction in mitochondrial biogenesis can be an essential trigger of tubular reprograming to fibrotic phenotype in 5/6Nx model.

In several models of renal damage PINK–Parkin-mediated mitophagy has been reported as the main mechanism involved in removing damaged mitochondria [38,39,40] However, in the periods evaluated in this study, we did not observe significant changes in Parkin and PINK levels in mitochondria isolated from remnant kidneys (Figure 5A). In contrast, the macroautophagy markers p62, LC3B-I, and LC3B-II progressively increased with time (Figure 5B), implying the induction of autophagy. In addition, electron microscopy showed, at day 28, substantial evidence of autophagy and mitochondria near autophagy bodies (Figure 4C), suggesting that this mechanism would principally be present later. However, deeper studies are still necessary to determinate the participation of these mechanism in the reduction in mitochondrial mass after nephrectomy, such as the evaluation of PINK–Parkin-independent autophagy markers over time.

Ours results also showed a progressive shift of mitochondrial dynamics from fusion to fission (Figure 3). At day 1, after 5/6Nx, the increase in MFN1 and OPA1 proteins and the decrease in FIS1 and DRP1 levels in mitochondria lead to larger mitochondrial size in the proximal tubule [14]. Morphometric analysis in this section confirmed this mitochondrial volume enlargement, reaching its maximum at day 14 (66% compared to control) [22]. We also noticed at this time point that mtDNA did not increase and that mitochondrial biogenesis was reduced (Figure 2). Other authors also concluded that mitochondria underwent pathologic hypertrophy (size enlargement) rather than proliferation [22,41]. However, this tendency changes along with CKD progression; in fact, our results showed that DRP1 increased 28 days after surgery (Figure 3A,B) and fusion proteins MFN2 and OPA1 started to increase from day 2 (Figure 3A,C), whereas mitochondrial fragmentation was observed by electron microscopy after day 7 (Figure 4). Taken together, these data suggest the gradual and slow shift to mitochondrial fission. In agreement with others, we reported an increase in mitochondrial fragmentation at day 28 [15,42] without changes in mtDNA. This tendency persisted even at 8 weeks after surgery [33]. Therefore, our data suggest that, in the remnant kidney, the interval from day 2 to day 28 led to a shift from mitochondrial fusion to fission in this model (Figure 3 and Figure 4).

**Figure 6:**
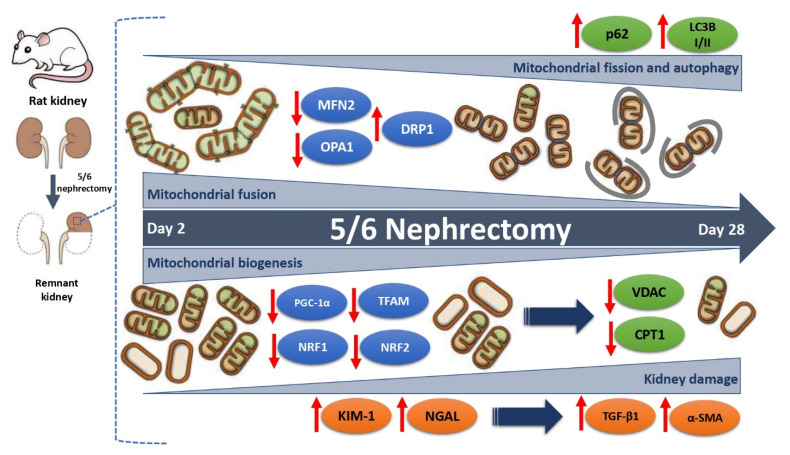
Evolution of mitochondrial biogenesis and dynamics changes in the time interval between day 2 and day 28 after 5/6 nephrectomy (5/6Nx). In this interval, there is a progressive reduction in the levels of peroxisome proliferator-activated receptor gamma coactivator 1-alpha (PGC-1α), peroxisome proliferator-activated receptor alpha (PPARα), nuclear respiratory factor 1 (NRF1) and 2 (NRF2), and mitochondrial transcription factor A (TFAM), which implies the downregulation of the mitochondrial biogenesis machinery over time. This reduction appears from day 2 and is progressive over time, triggering the decrease in mitochondrial proteins such as voltage-dependent anion channel (VDAC) and carnitine palmitoyltransferase 1 (CPT1) from day 2 to day 28. There is a positive correlation between the reduction in mitochondrial biogenesis factors and the increase in the tubular damage proteins kidney injury molecule-1 (KIM-1) and neutrophil gelatinase-associated lipocalin (NGAL) and the fibrotic markers transforming growth factor beta (TGF-β1) and alpha smooth muscle actin (α-SMA). In addition, a slow and gradual change in mitochondrial dynamics from fusion to fission is also shown. This trend started with a reduction on day 2 after 5/6Nx in the fusion proteins mitofusin 2 (MFN2) and optic atrophy 1 (OPA1), favoring the mitochondrial fragmentation observed from 7 days, and continued with an increase in dynamin-1-like protein (DRP1) 28 days after surgery. In addition, macroautophagy markers ubiquitin-binding protein p62 and microtubule-associated proteins 1A/1B light chain 3B I and II (LC3B-I/II) progressively increased with the time, implying the induction of autophagy. This mechanism would principally be present at a chronic stage.

We integrate the results in the diagram in Figure 6, in which we show the temporal progression of the changes in mitochondrial biogenesis and dynamics in the time interval between day 2 and day 28 after 5/6Nx, as well as its relationship with the progression of renal damage. Taking into account the data obtained in this manuscript, mitochondria may be an effective target to attenuate renal damage. Mitochondrion-targeted therapy would help in maintaining mitochondrial homeostasis and, thus, in preventing the development of CKD as previously suggested [43,44].

## 5. Conclusions

As demonstrated herein, 5/6Nx induces a progressive reduction in mitochondrial mass via a decrease in mitochondrial biogenesis, as well a slow shift from mitochondrial fission to fusion in remnant kidney. These pathological changes contribute to the impairment in mitochondrial ATP production and β-oxidation, favoring CKD progression in this model.

## Data Availability

Not applicable.

## Supplementary Materials

The following are available online at https://www.mdpi.com/article/10.3390/biology10050349/s1: Figure S1. Uncropped blots and densitometric quantification.
