# Supplementary material for: Progressive Reduction in Mitochondrial Mass Is Triggered by Alterations in Mitochondrial Biogenesis and Dynamics in Chronic Kidney Disease Induced by 5/6 Nephrectomy
Source: Biology (Basel). 2021 Apr 21;10(5):349. doi: 10.3390/biology10050349 (PMC8143166; doi:10.3390/biology10050349)
Supplement: Supplementary file 1 [file biology-10-00349-s001.zip › biology-1185419-supplementary.pdf]

KIM-1, β-Actin (Figure 1)

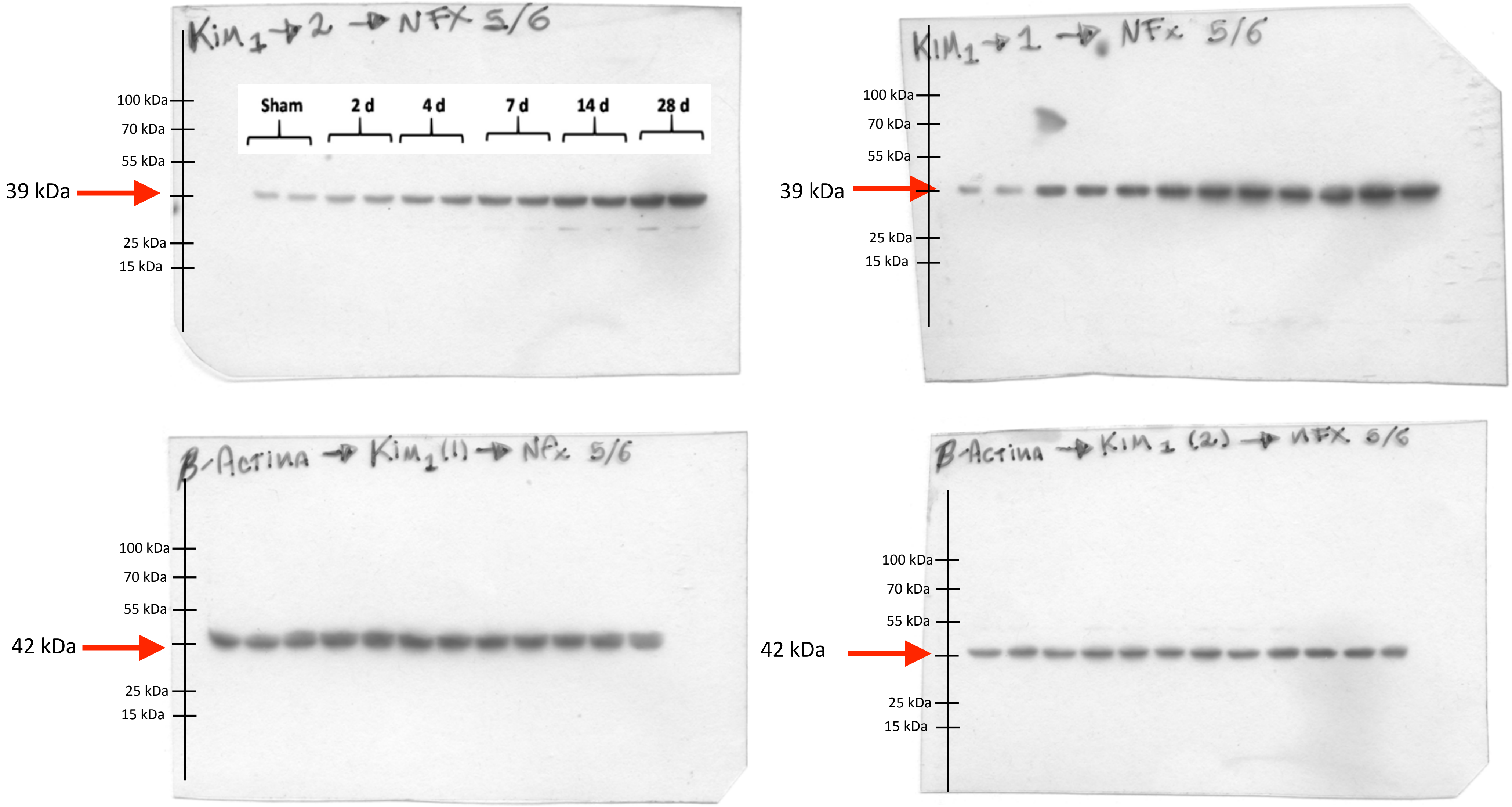

NGAL, β-Actin (Figure 1)

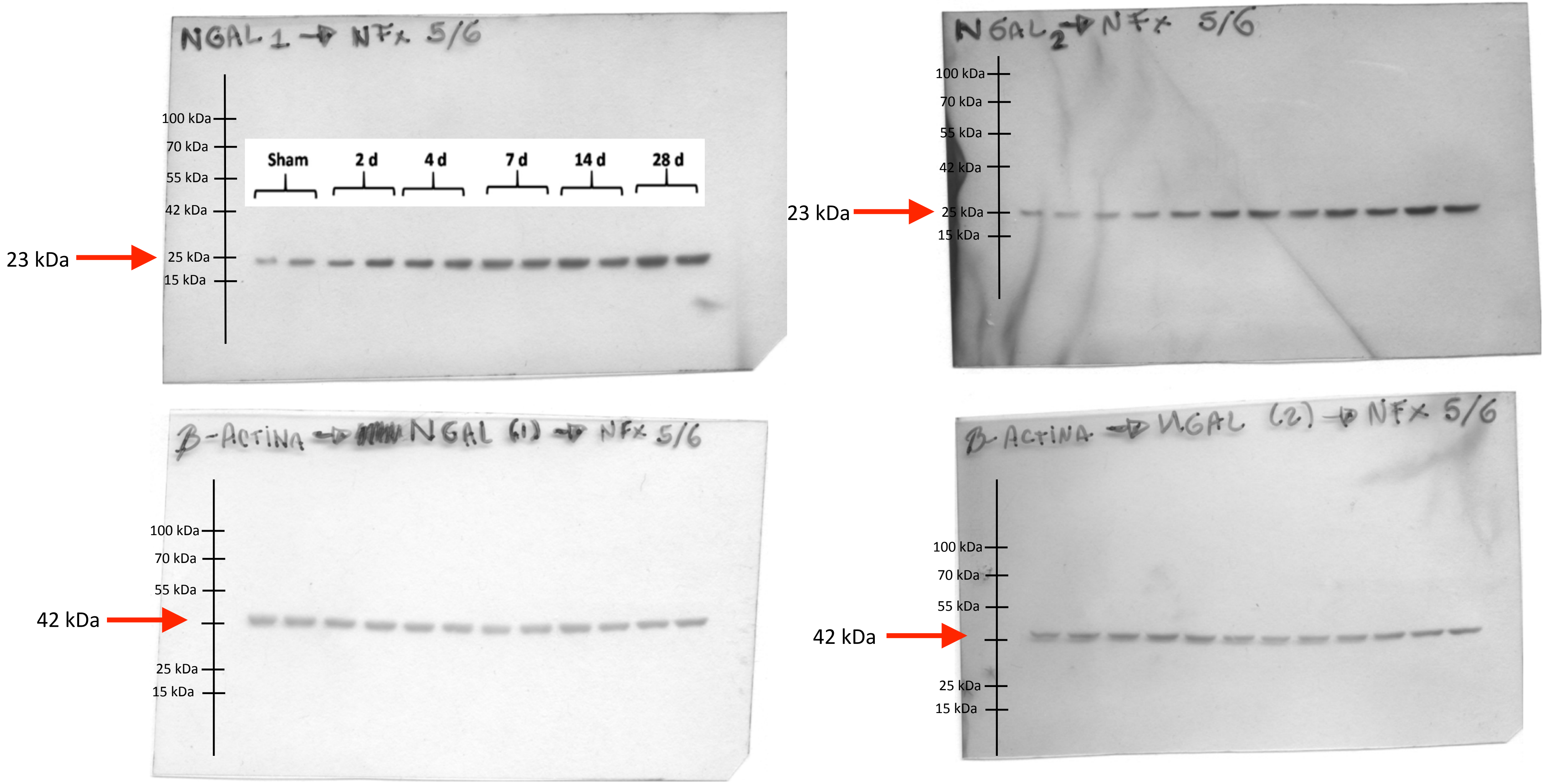

TGF-β1, β-Actin (Figure 1)

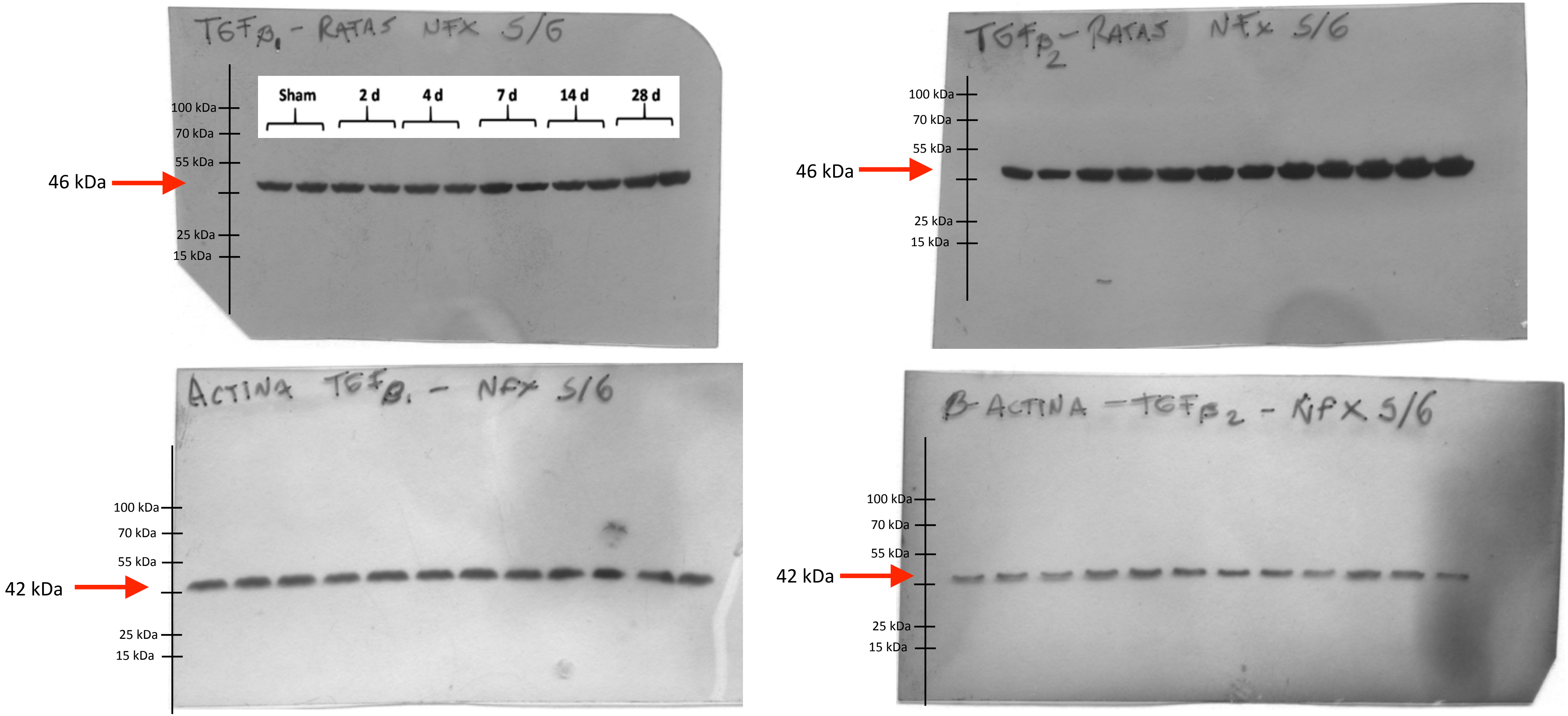

α-SMA, β-Actin (Figure 1)

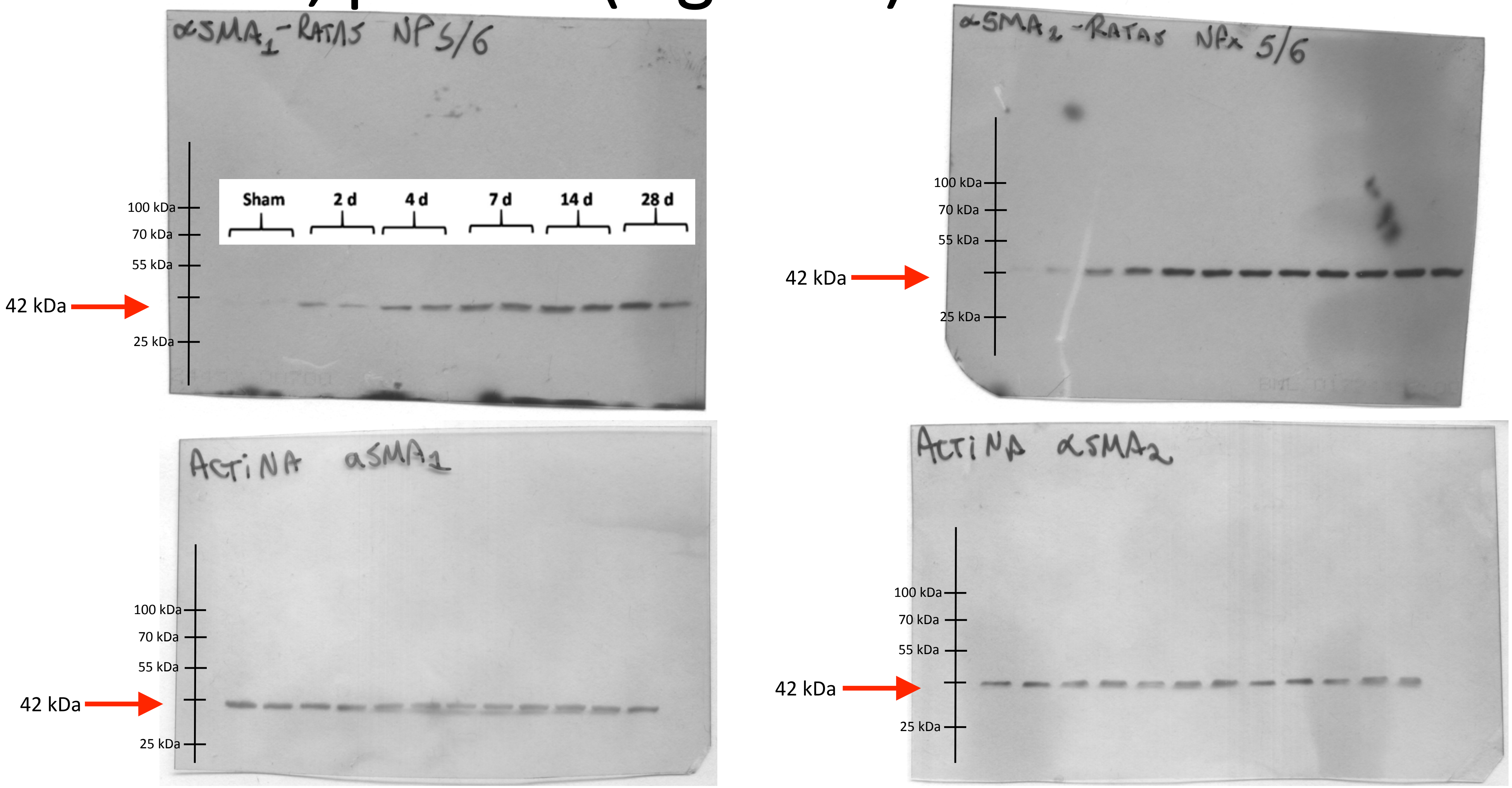

VDAC, β-Actin (Figure 2)

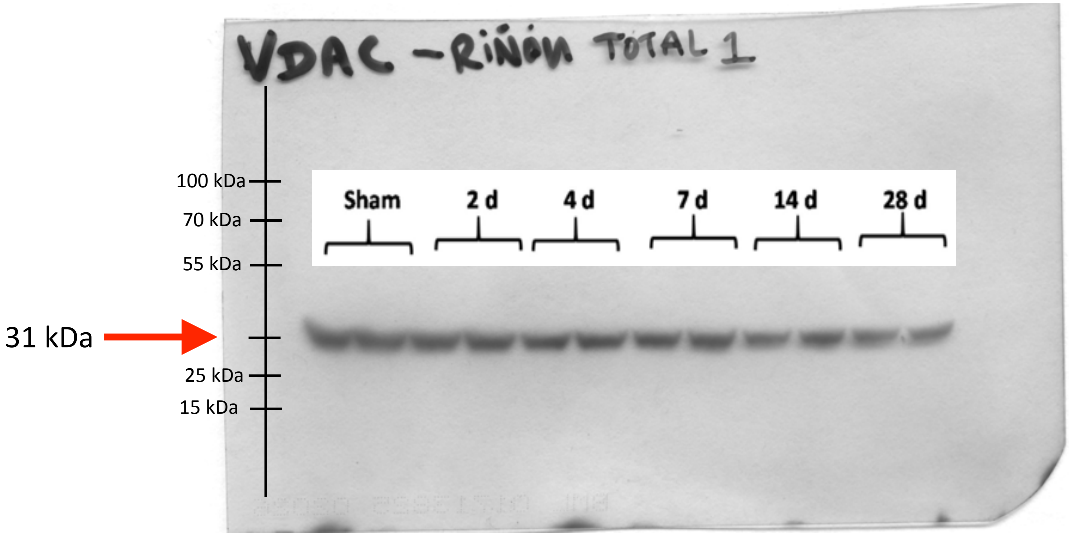

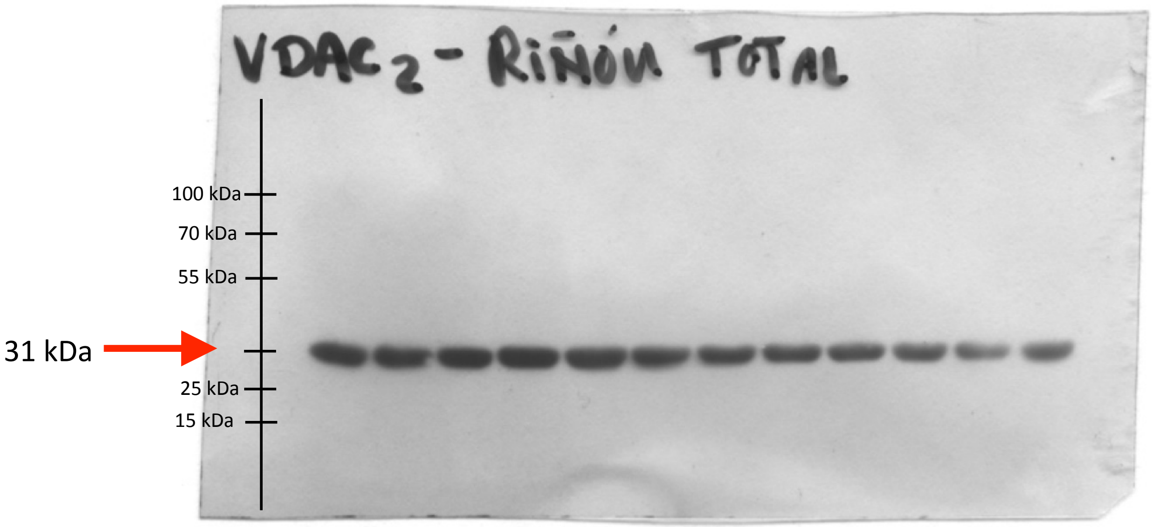

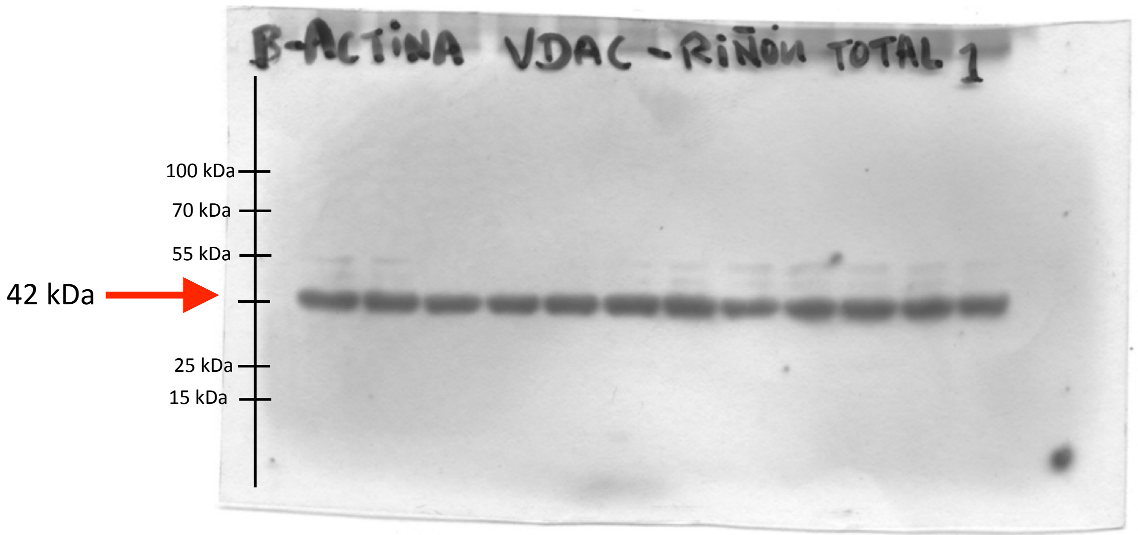

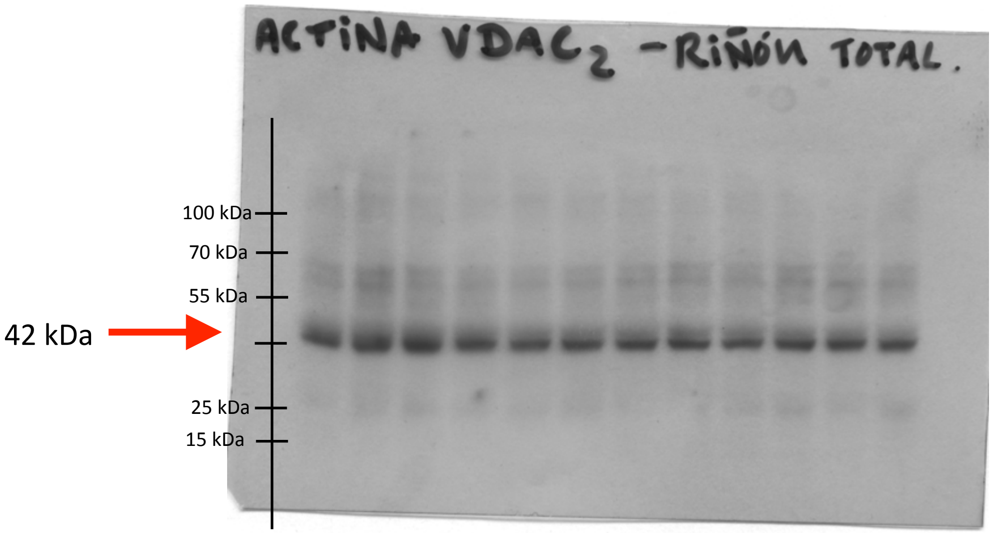

PGC-1α, β-Actin (Figure 2)

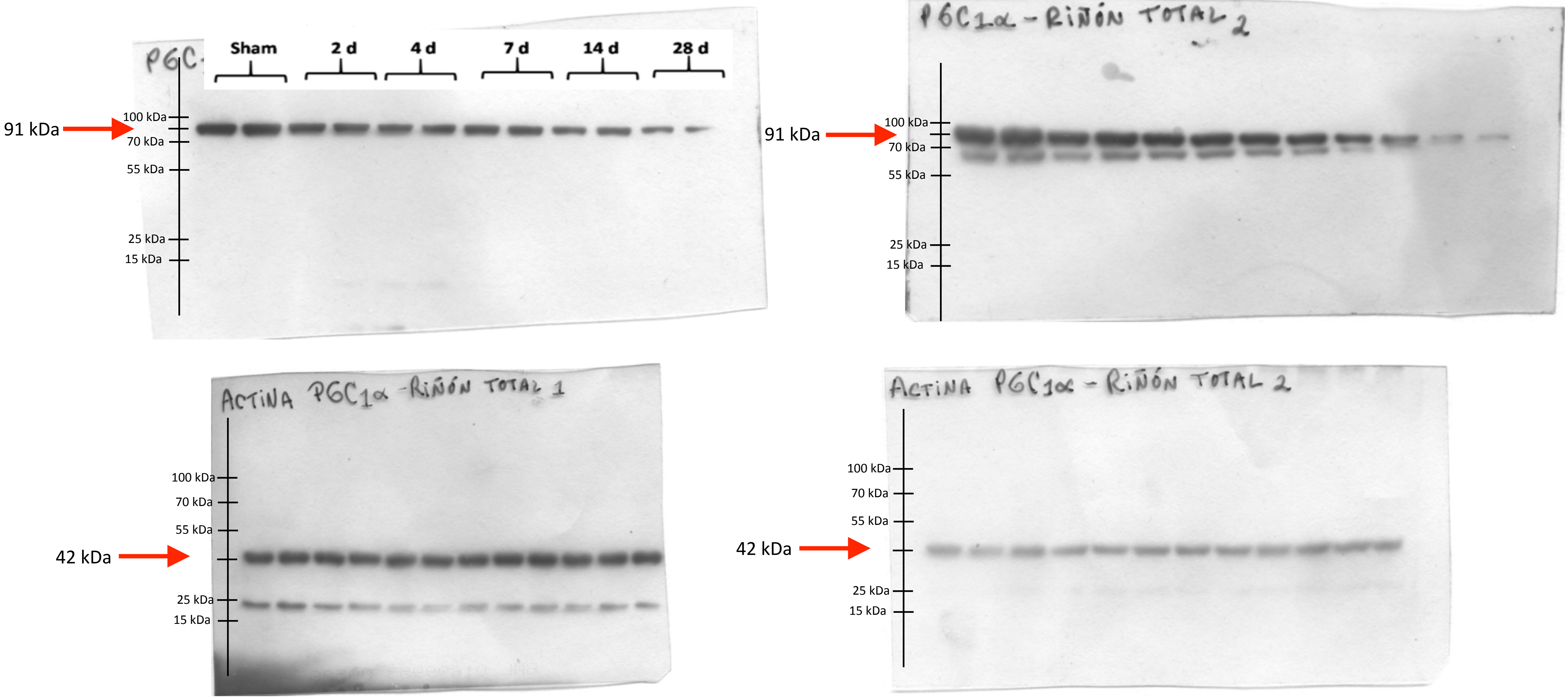

NRF1, β-Actin (Figure 2)

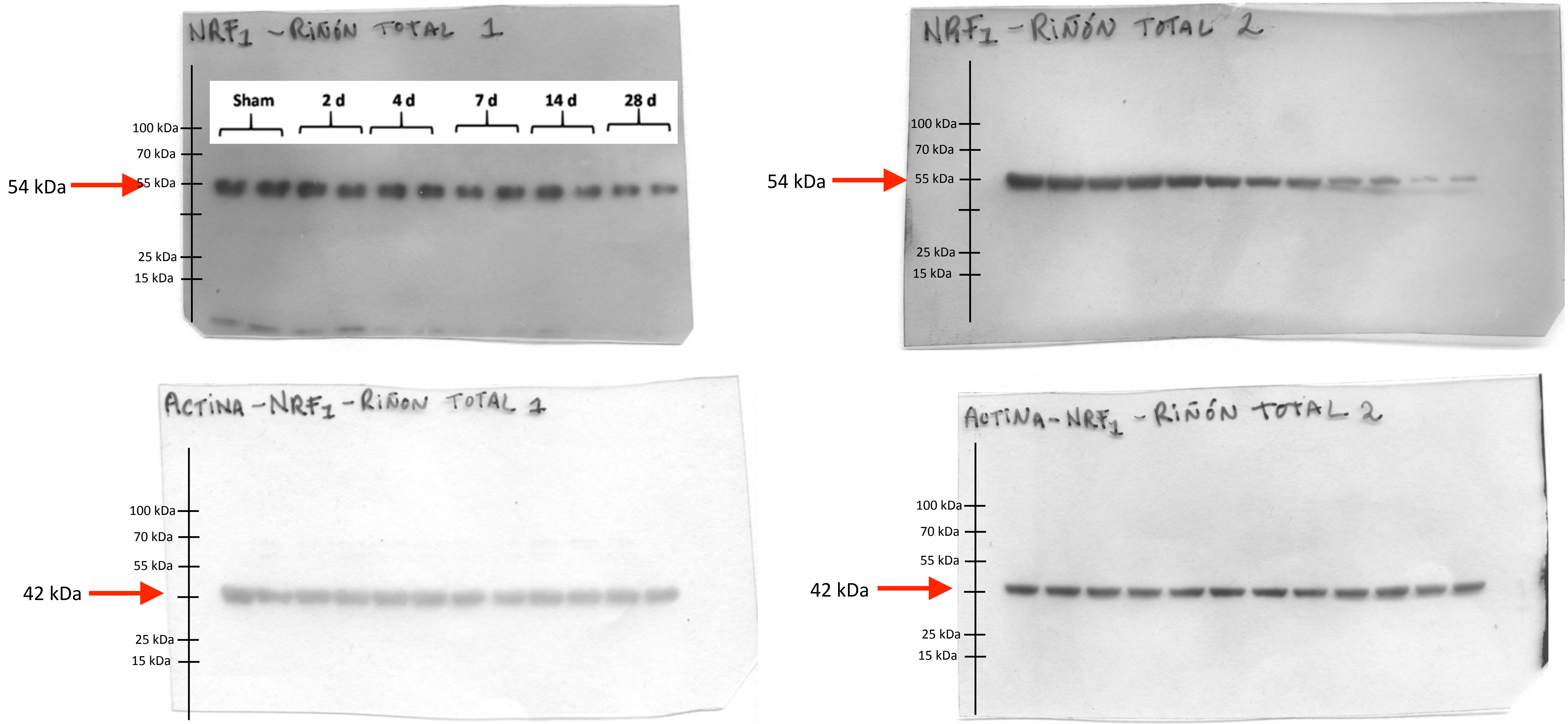

NRF2, β-Actin (Figure 2)

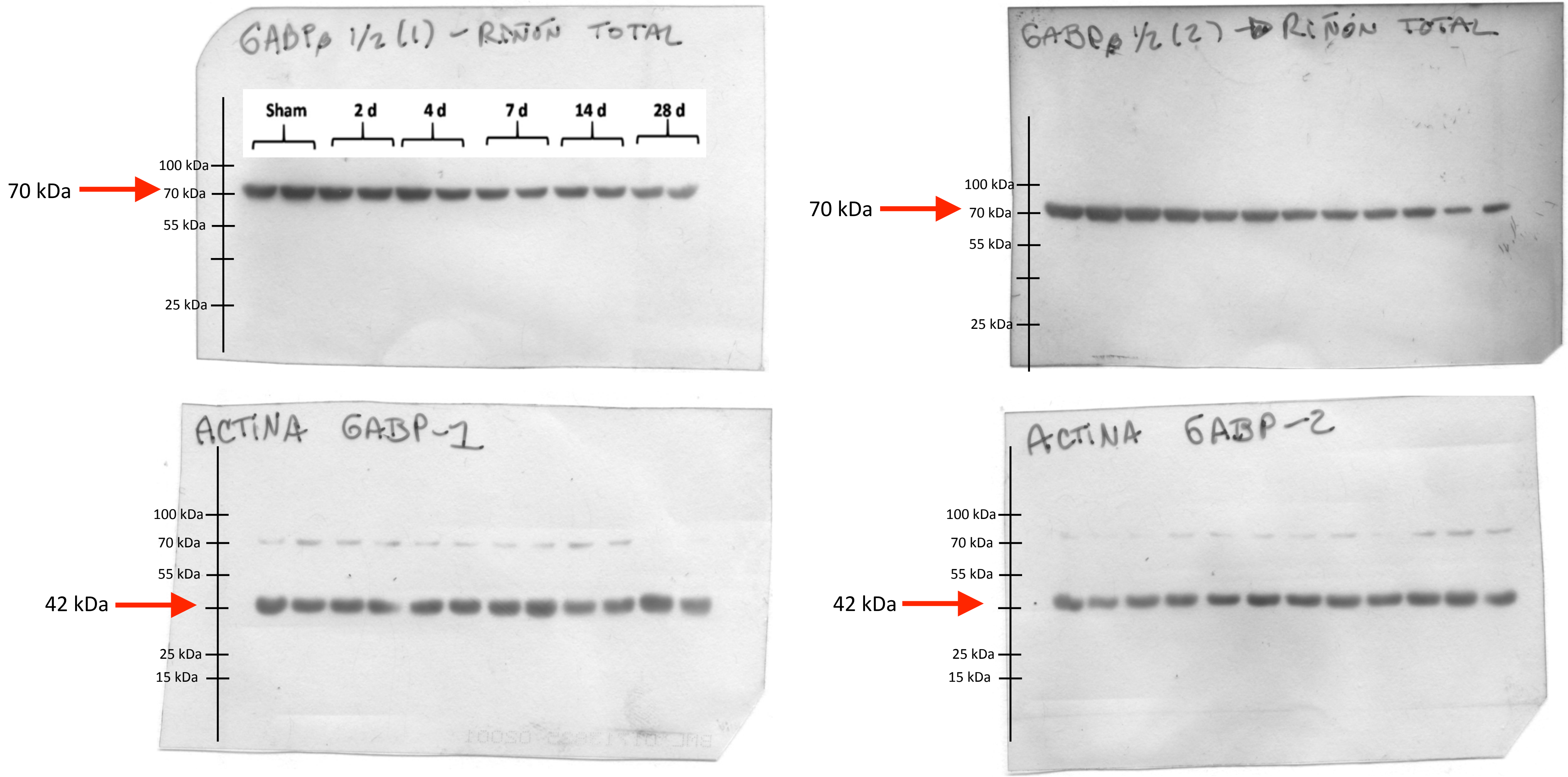

TFAM, β-Actin (Figure 2)

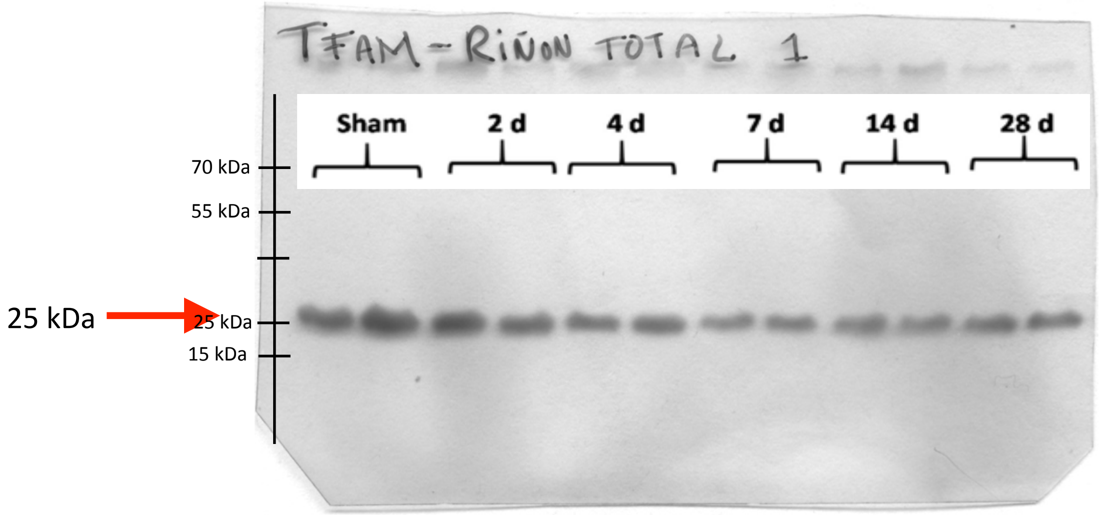

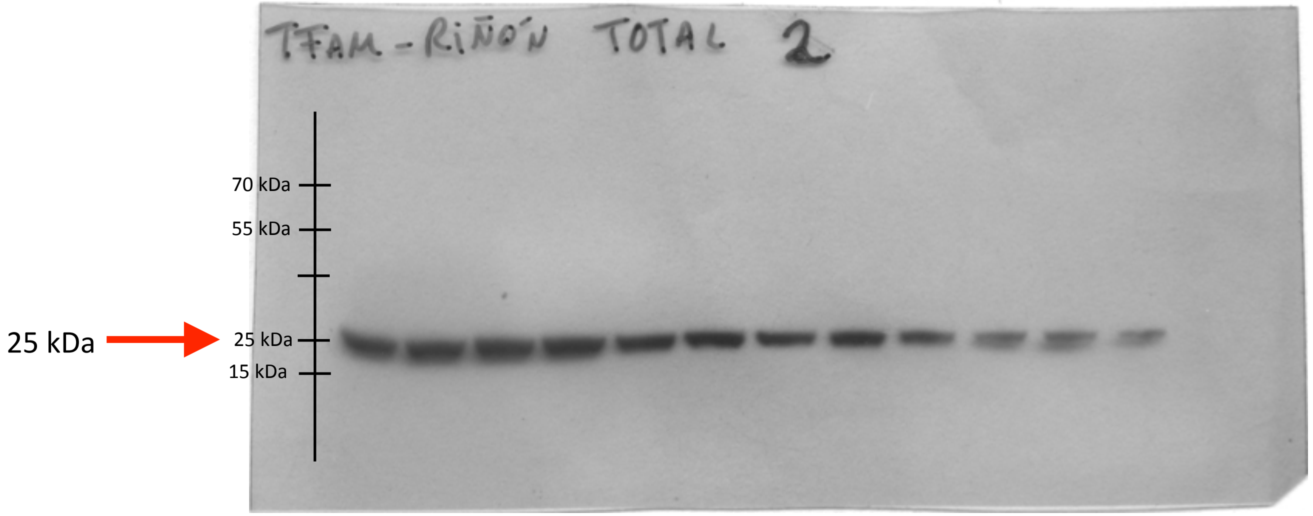

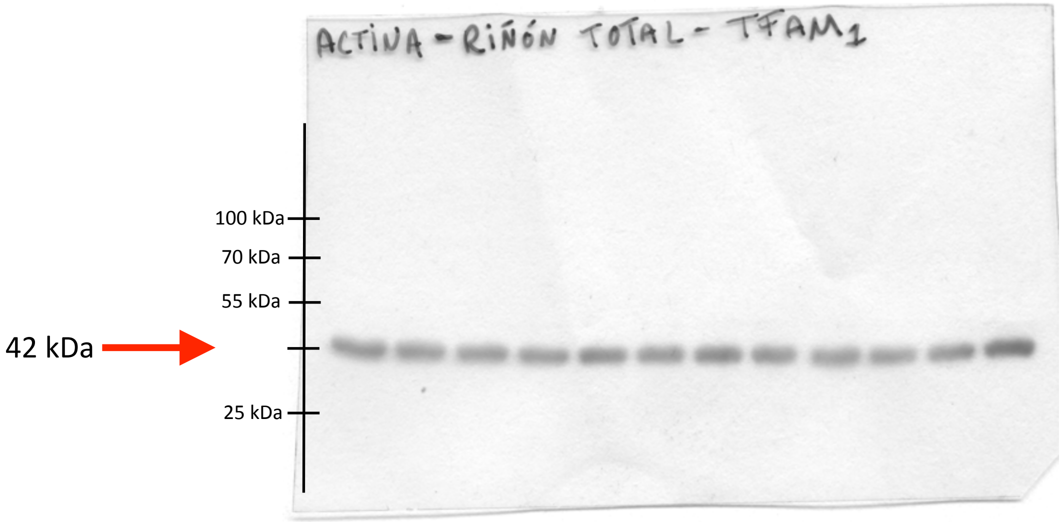

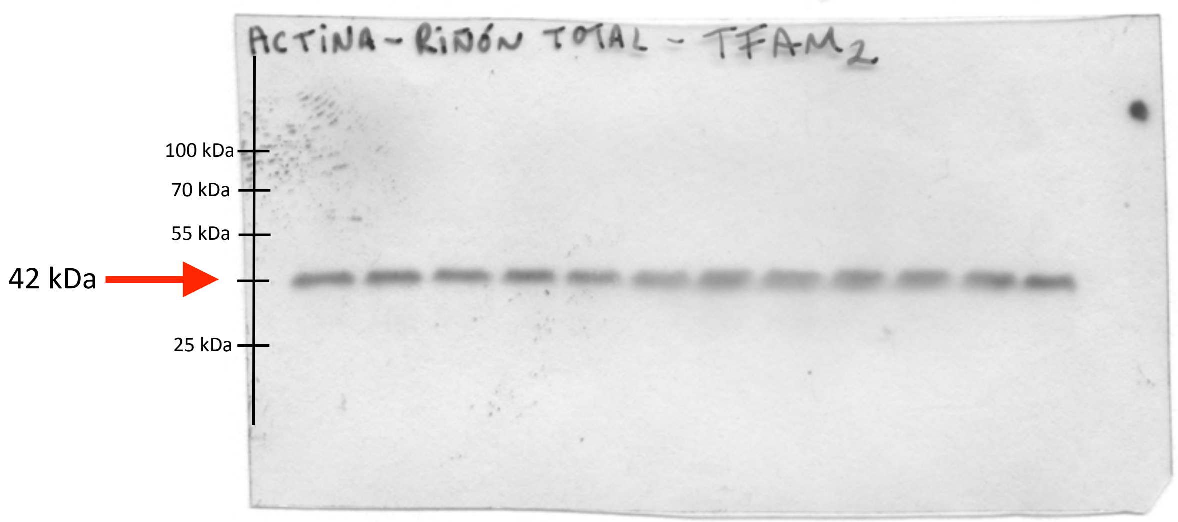

CPT1, β-Actin (Figure 2)

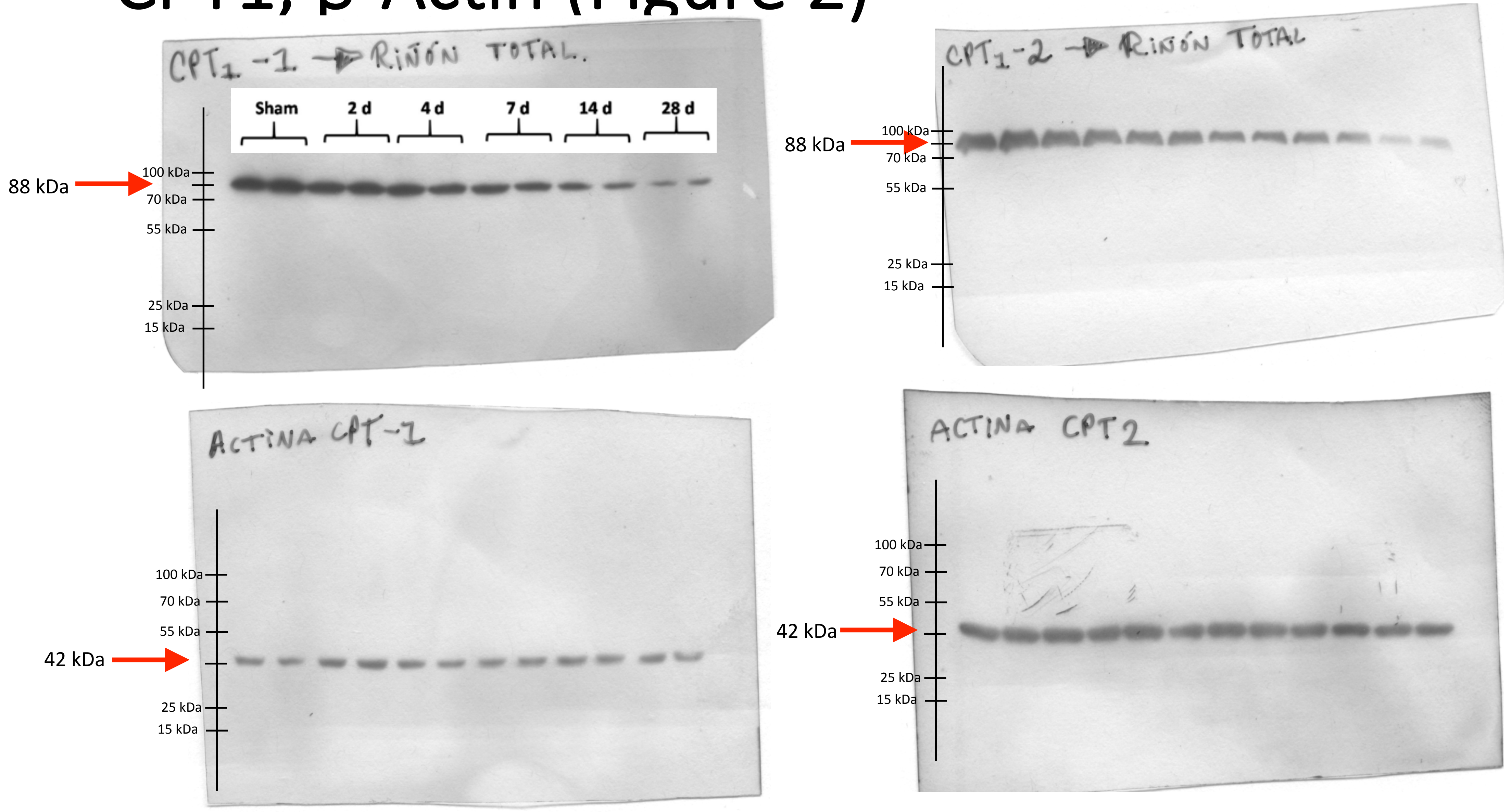

PPARα, β-Actin (Figure 2)

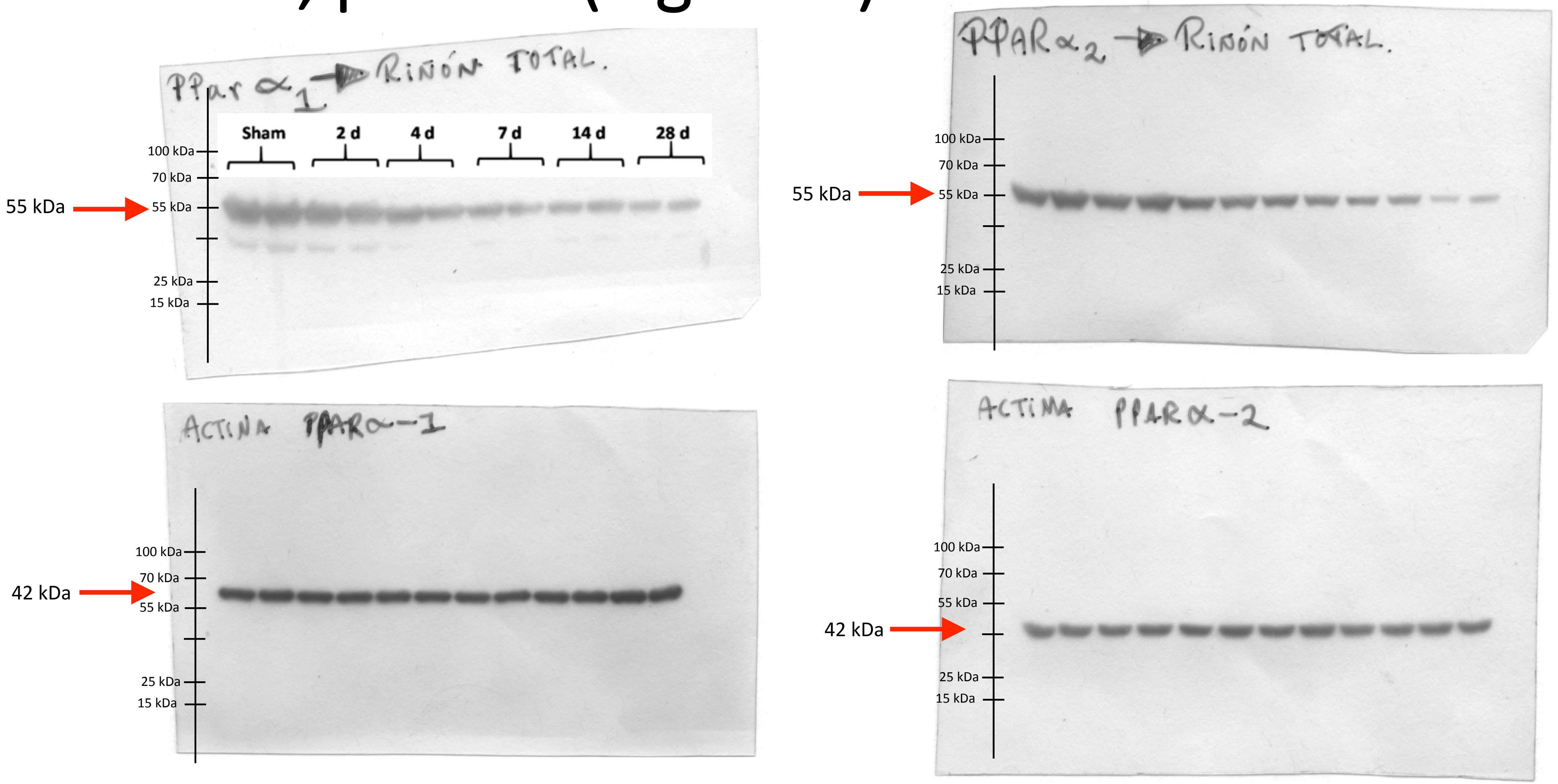

DRP1, VDAC (Figure 3)

90 kDa

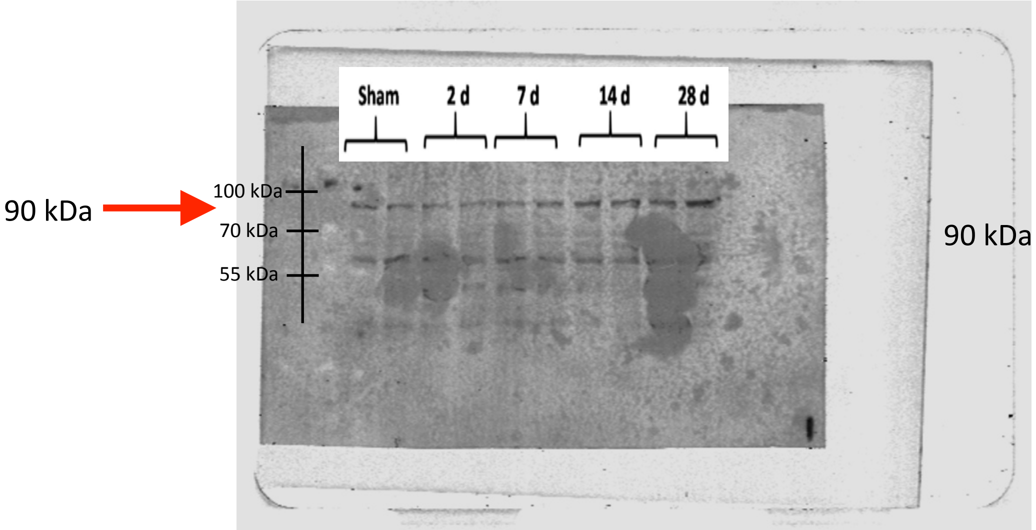

90 kDa

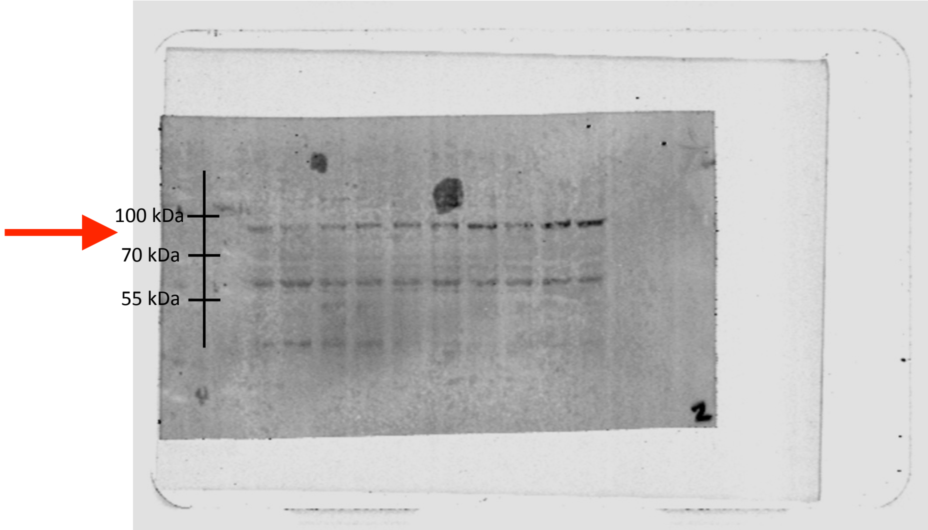

31 kDa

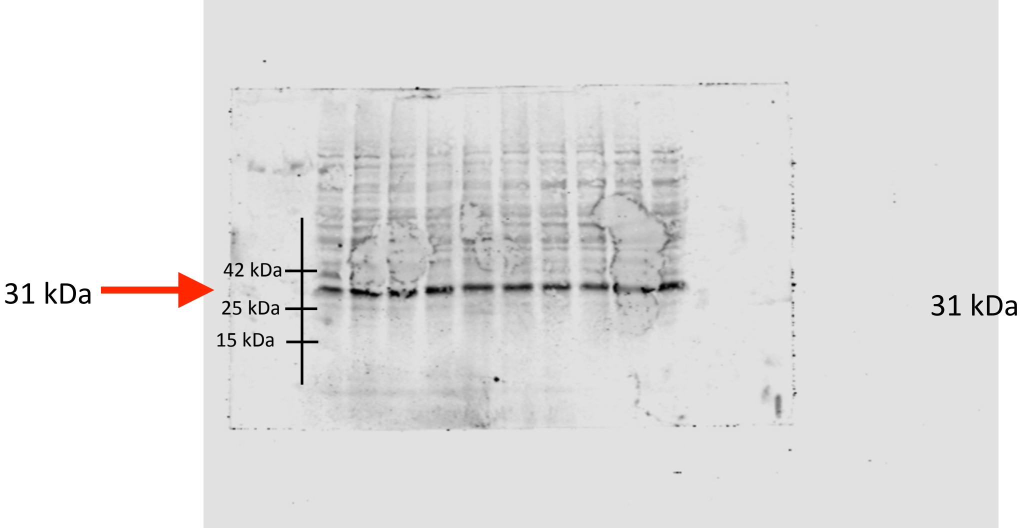

31 kDa

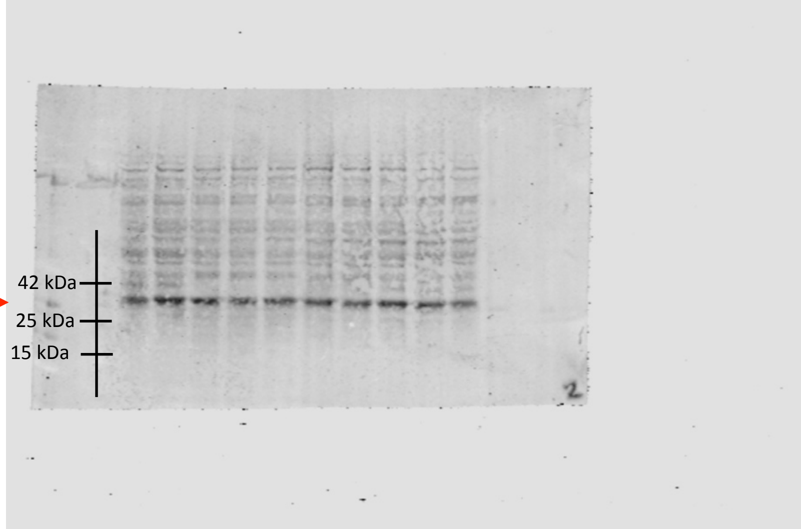

FIS1, VDAC (Figure 3)

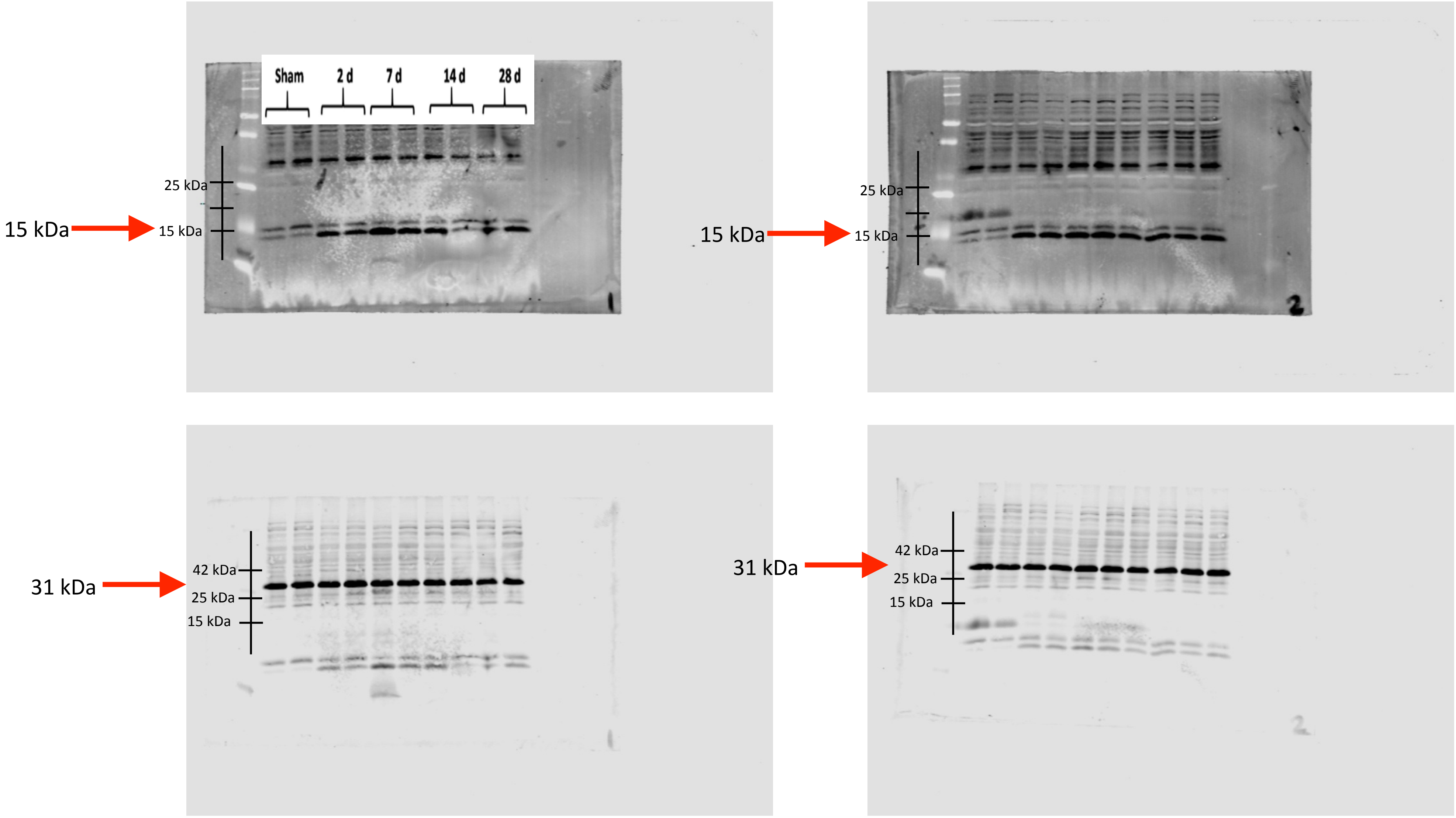

MFN1, VDAC (Figure 3)

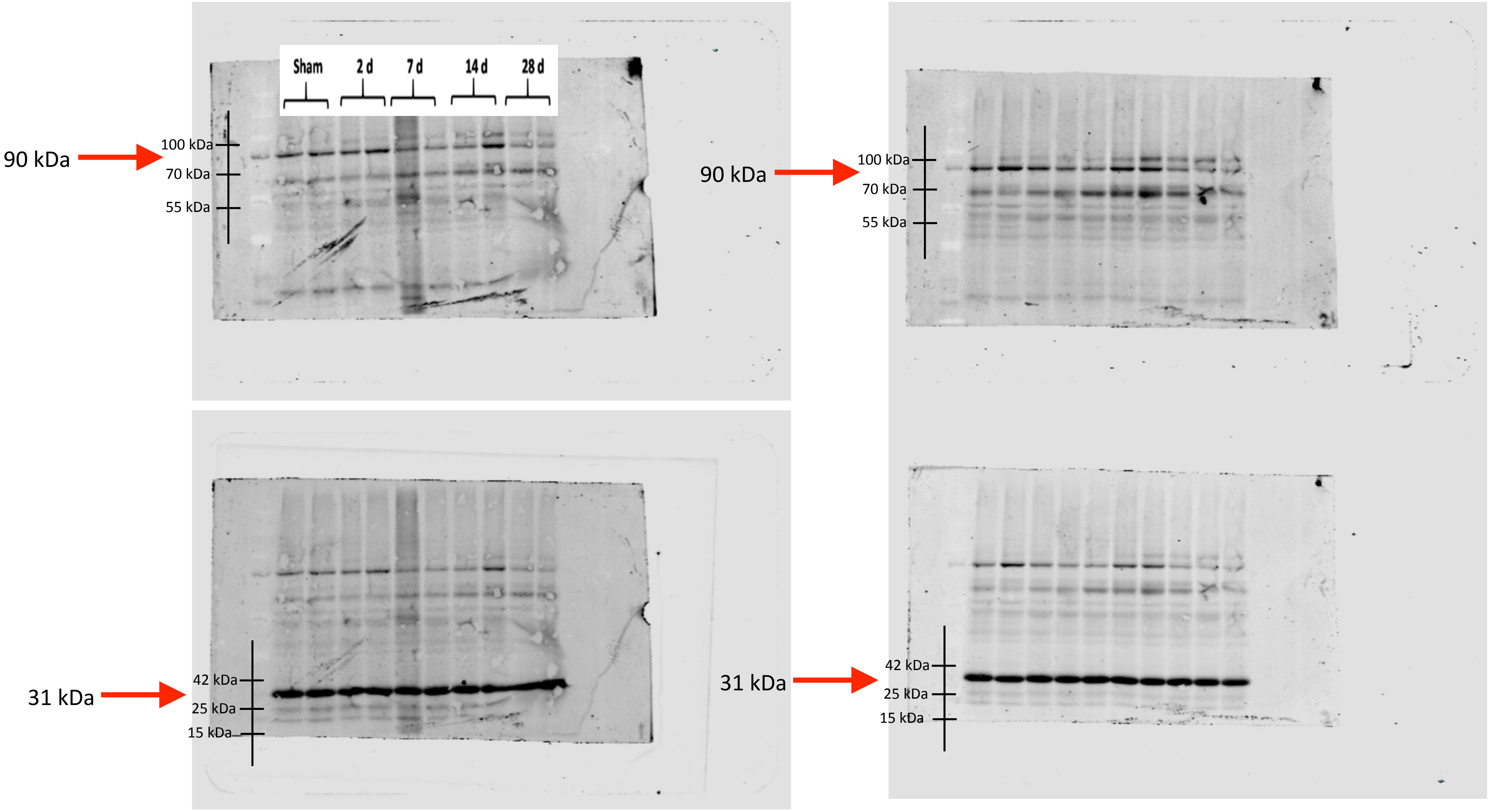

MFN2, VDAC (Figure 3)

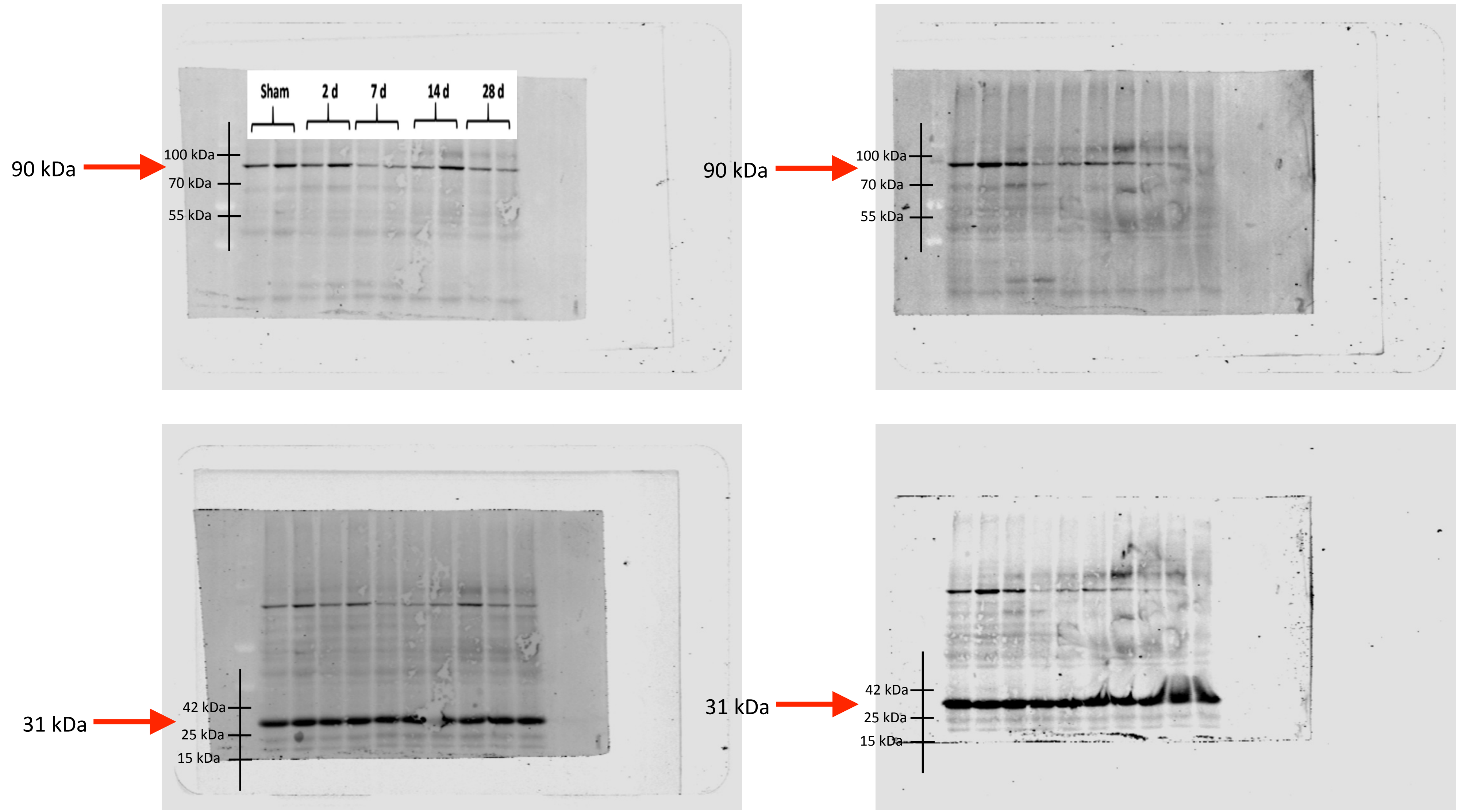

OPA1, VDAC (Figure 3)

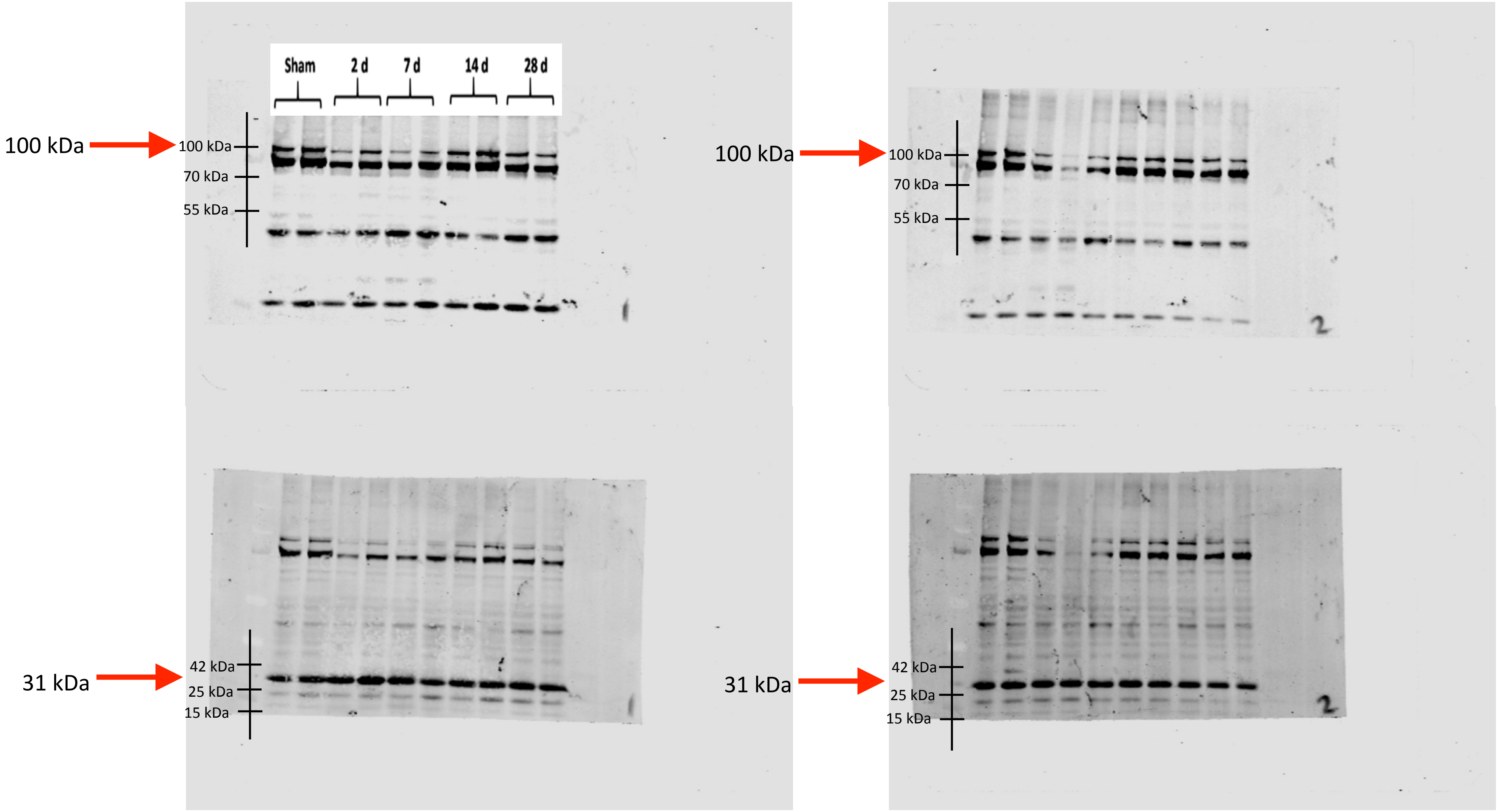

PINK1, VDAC (Figure 5)

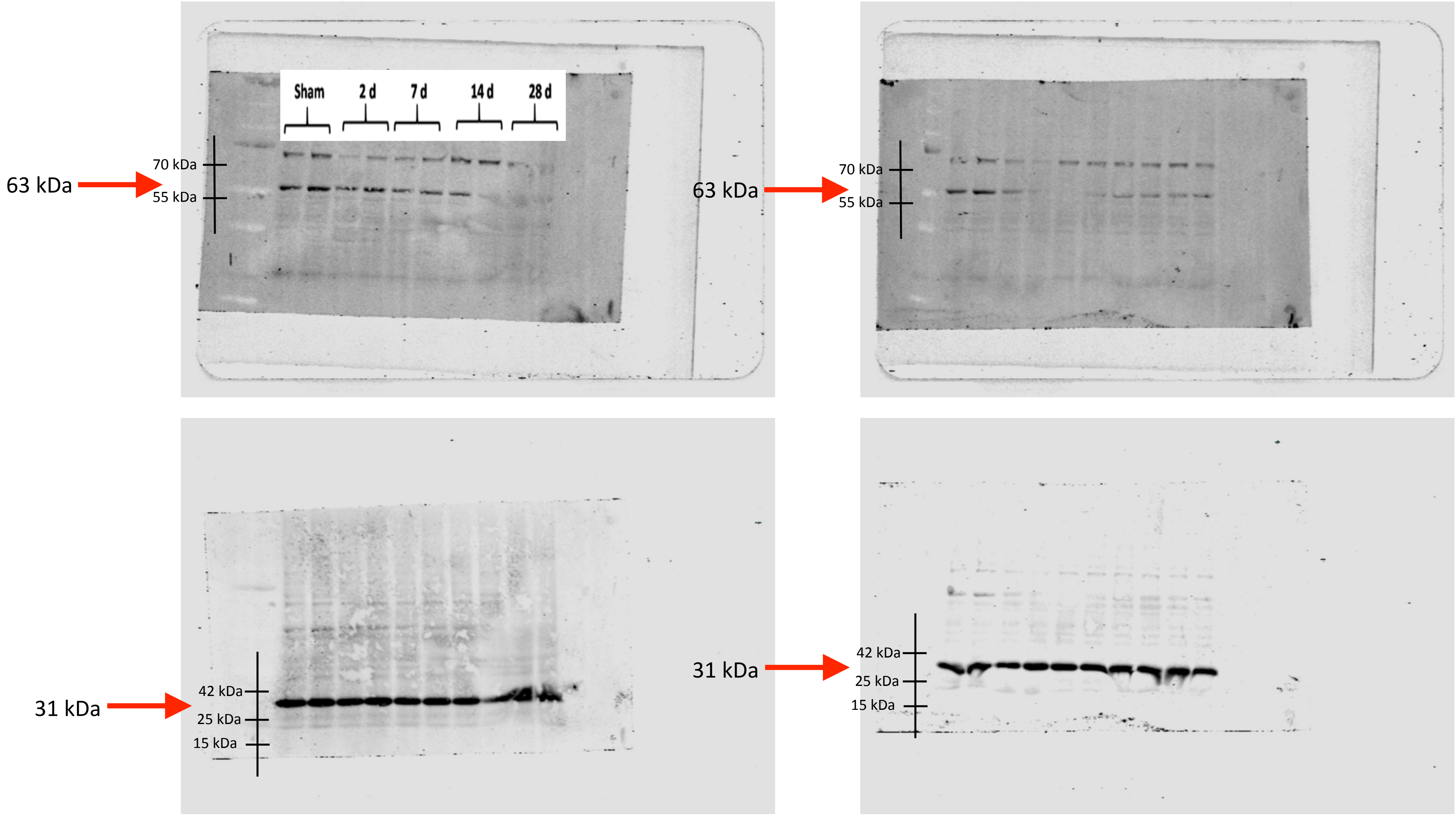

Parkin, VDAC (Figure 5)

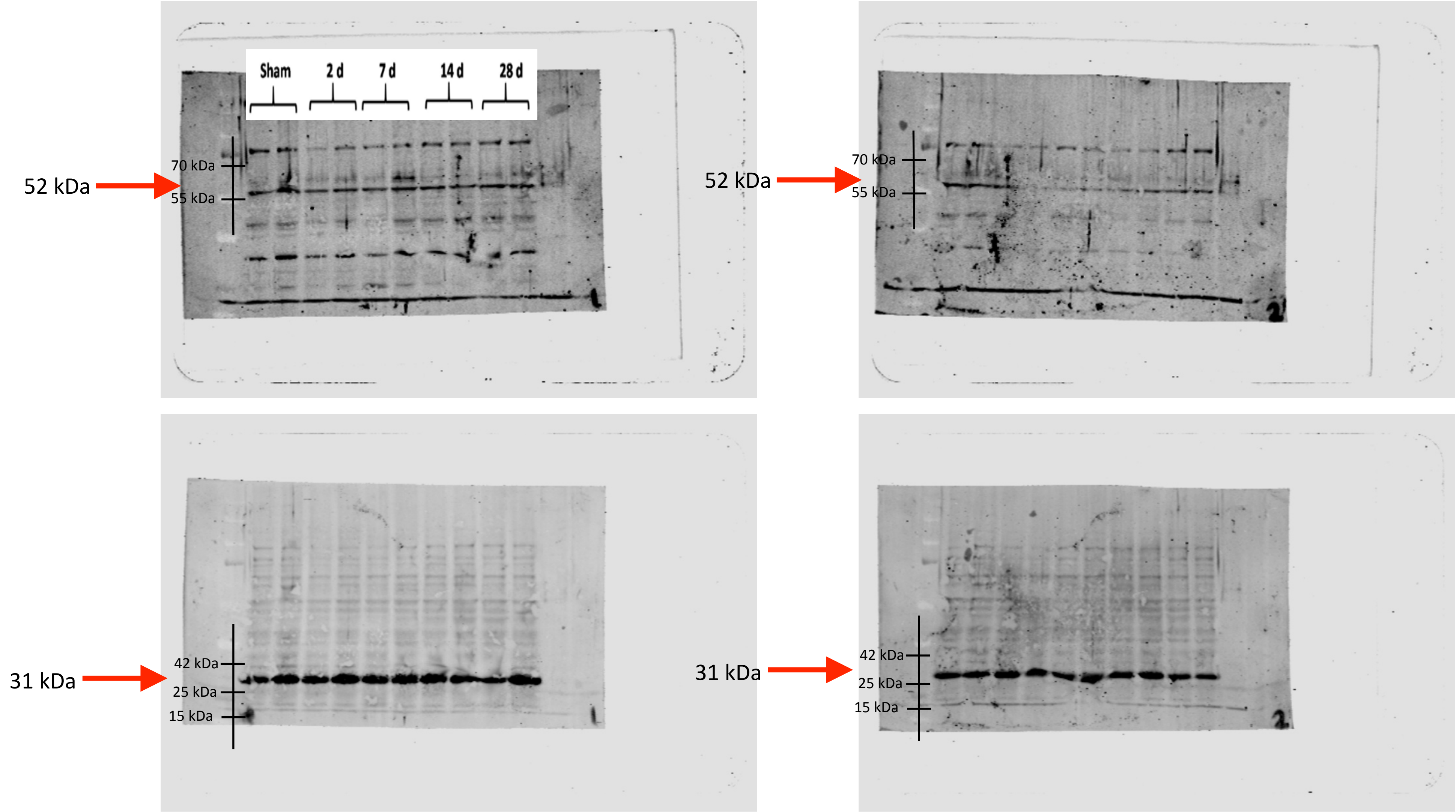

p62, β-Actin (Figure 5)

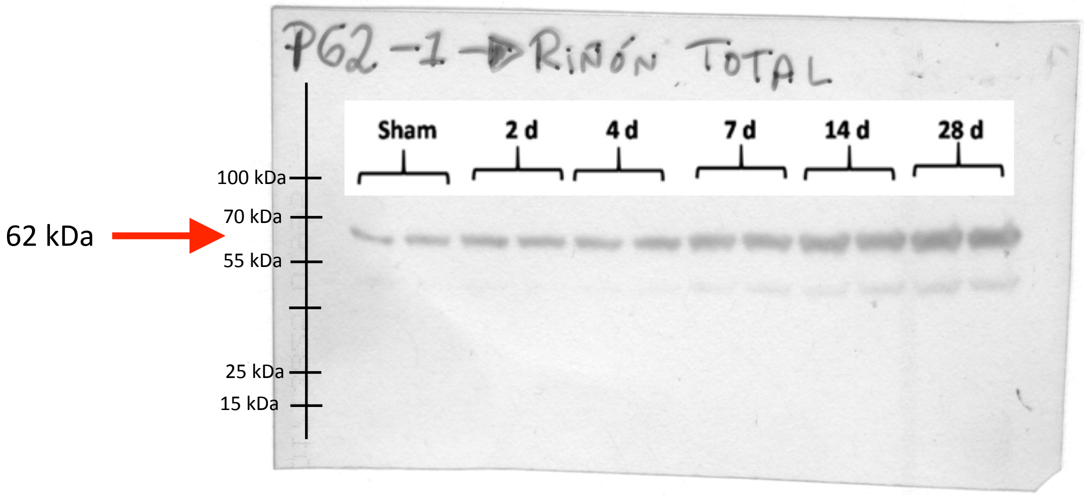

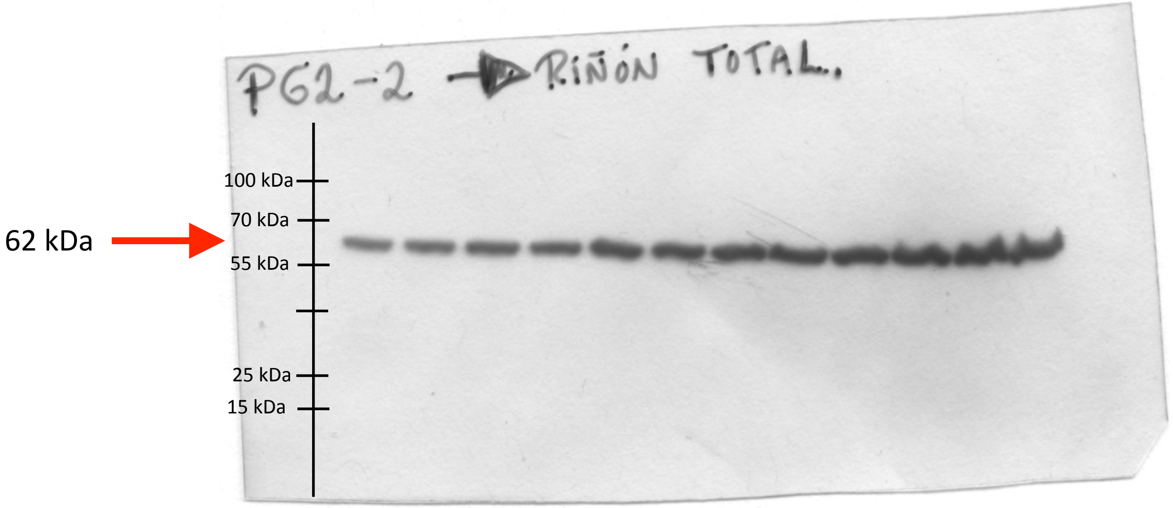

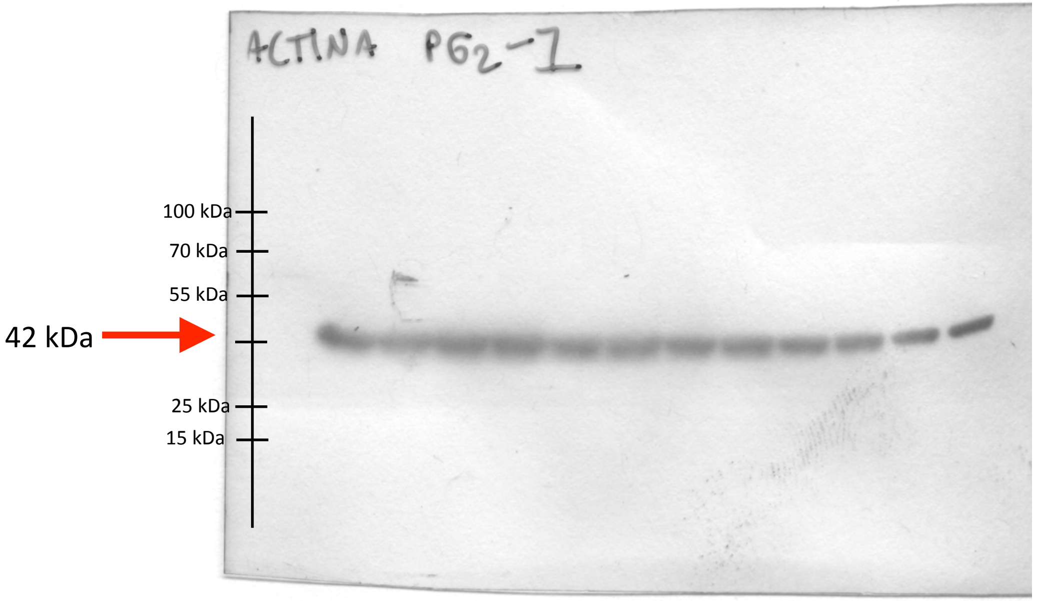

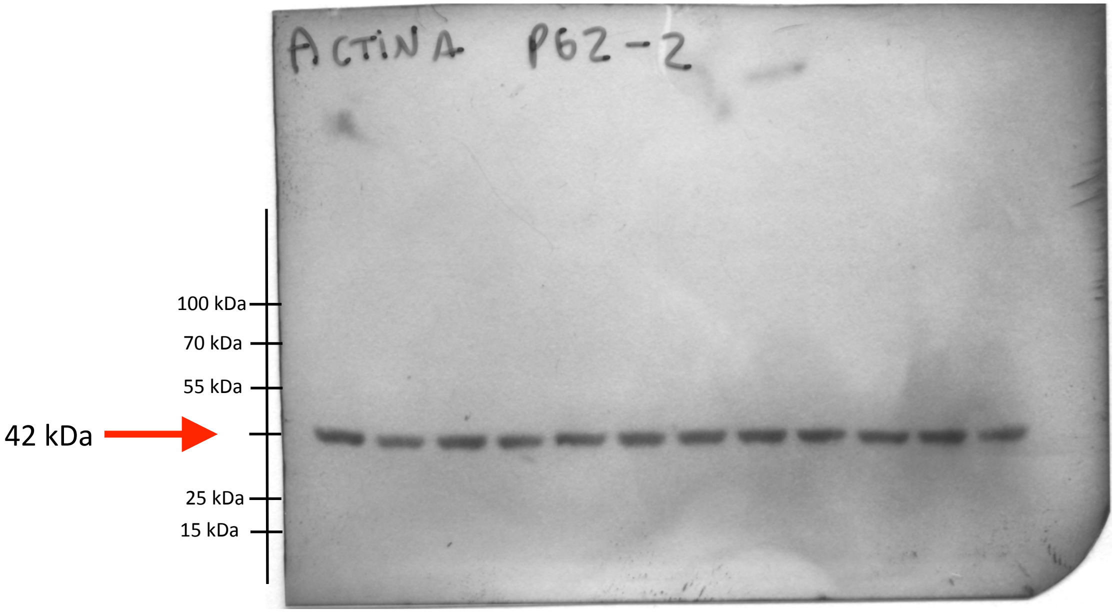

LC3B I/II, β-Actin (Figure 5)

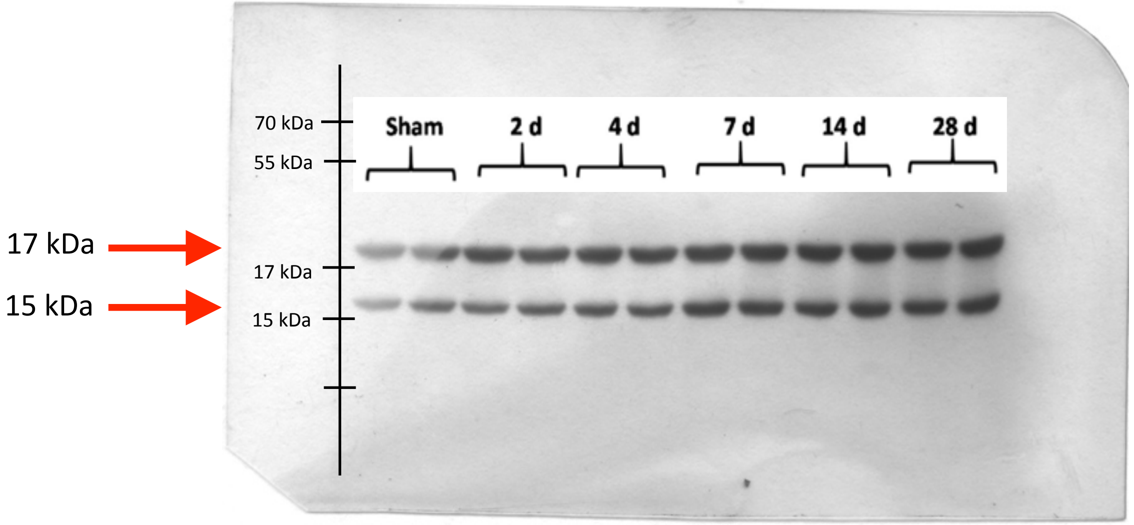

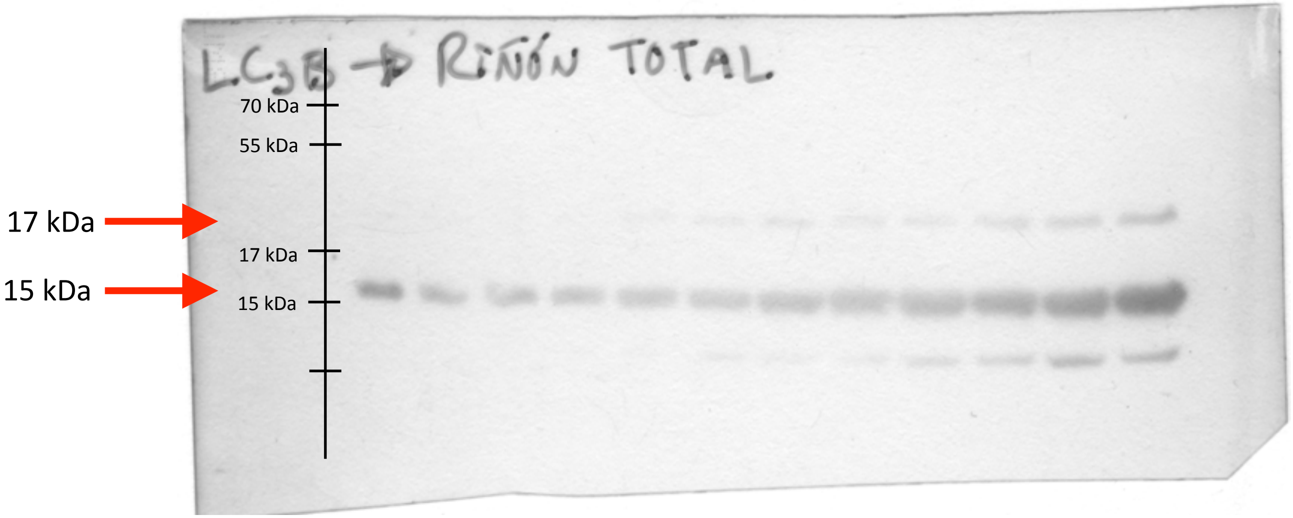

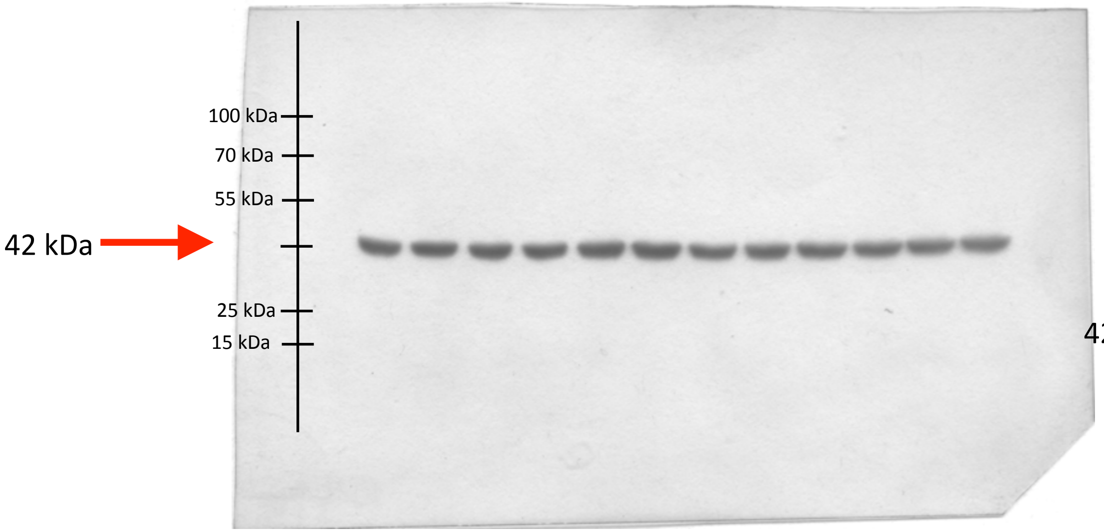

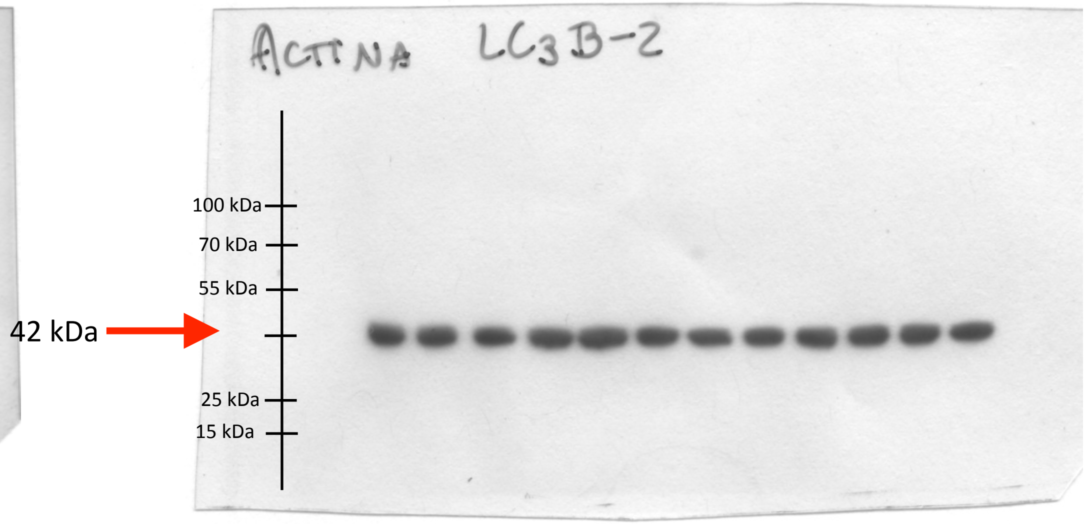

KIM-1, β-Actin (Figure 1)

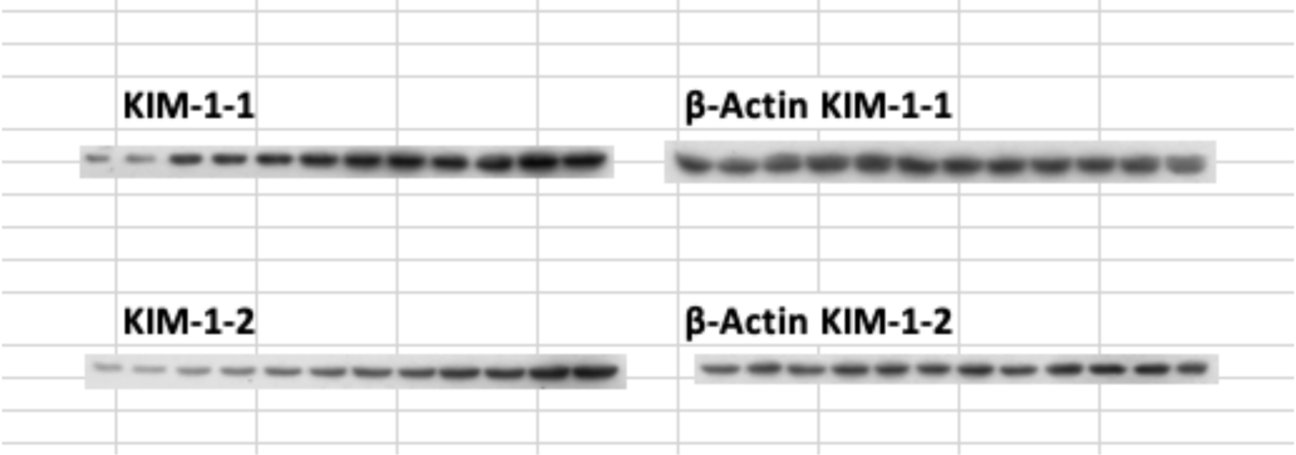

Group	KIM-1-1	β-Actin KIM-1-1	KIM-1-2	β-Actin KIM-1-2	KIM-1-1/β-Actin	KIM-1-2/β-Actin	Correction 1	Correction 2
Control	0.066723969	0.176636475	0.118006586	36.32730531	0.3777474	0.003248427	1.008292177	0.999999885
Control	0.066968934	0.178755041	0.128366399	35.60631693	0.374640812	0.003605158	1	1.109816539
Nx5/6 (2 days)	0.107720502	0.179469239	0.16822378	33.6409086	0.60021708	0.005000572	1.60211344	1.539382553
Nx5/6 (2 days)	0.104541276	0.180583141	0.173554018	33.92926172	0.57890939	0.005115172	1.545238457	1.574661287
Nx5/6 (4 days)	0.129765026	0.175358165	0.220603577	32.06912059	0.73999991	0.006879003	1.975225033	2.117641246
Nx5/6 (4 days)	0.129826374	0.17540786	0.220693687	32.28952664	0.740140007	0.006834838	1.975598981	2.104045467
Nx5/6 (7 days)	0.188325488	0.181331507	0.276619307	30.21562924	1.038570141	0.009154842	2.772175661	2.818238444
Nx5/6 (7 days)	0.189174973	0.176573983	0.277867061	30.33962246	1.071363797	0.009158554	2.859709253	2.819381101
Nx5/6 (14 days)	0.264168752	0.181355831	0.388020643	29.7268097	1.456632244	0.013052885	3.888076785	4.018217274
Nx5/6 (14 days)	0.251221635	0.1760442	0.369003448	28.19105189	1.427037271	0.013089382	3.809081191	4.029452416
Nx5/6 (28 days)	0.322296078	0.17902	0.473400167	28.25897627	1.800335591	0.016752205	4.80549778	5.157020654
Nx5/6 (28 days)	0.326284369	0.183153858	0.479258314	28.87775608	1.781476912	0.016596106	4.755159757	5.108967033

NGAL, β-Actin (Figure 1)

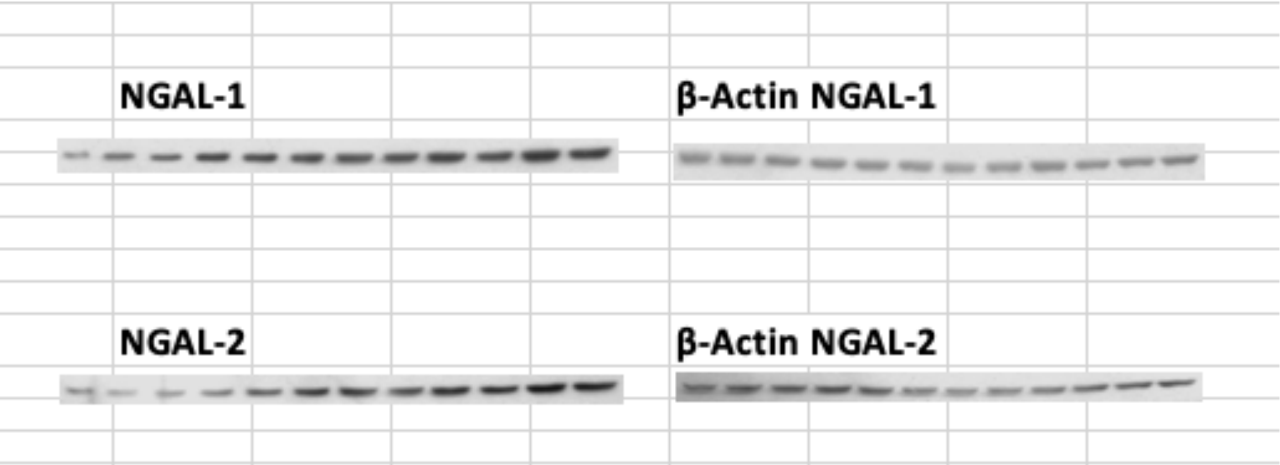

Group	NGAL-1	β-Actin NGAL-1	NGAL-2	β-Actin NGAL-2	NGAL-1/β-Actin	NGAL-2/β-Actin	Correction 1	Correction 2
Control	7.476032535	7.531095346	1.974461573	5.177240242	0.992688605	0.381373373	1	1
Control	7.677664653	7.621422804	1.995016684	5.074487505	1.007379442	0.393146437	1.014799039	1.030870179
Nx5/6 (2 days)	12.01646214	7.651873416	3.012881019	4.794384398	1.570394789	0.628418743	1.581961132	1.647778235
Nx5/6 (2 days)	11.89821891	7.651873416	2.983233958	4.835479474	1.554941942	0.616946877	1.566394471	1.617697826
Nx5/6 (4 days)	14.0213729	7.651873416	3.515571205	4.570378678	1.832410462	0.76920786	1.845906615	2.016941703
Nx5/6 (4 days)	13.61444495	7.651873416	3.413542381	4.601790177	1.77923029	0.741785751	1.792334757	1.945038126
Nx5/6 (7 days)	18.32611457	7.651873416	4.594896742	4.306225588	2.394984022	1.067035771	2.412623667	2.797876955
Nx5/6 (7 days)	18.67879361	7.651873416	4.683323767	4.323896667	2.44107457	1.083125738	2.459053683	2.840066492
Nx5/6 (14 days)	25.59321928	7.651873416	6.416973954	4.236560739	3.344699773	1.514665869	3.369334307	3.971608865
Nx5/6 (14 days)	25.37404601	7.651873416	6.362020762	4.017689918	3.316056688	1.583502184	3.340480258	4.152104726
Nx5/6 (28 days)	29.27783729	7.651873416	7.340816226	4.027370264	3.826231264	1.822731893	3.854412395	4.779389497
Nx5/6 (28 days)	29.65233509	7.651873416	7.434713856	4.115556593	3.875173239	1.806490492	3.90371484	4.736802882

TGF-β1, β-Actin (Figure 1)

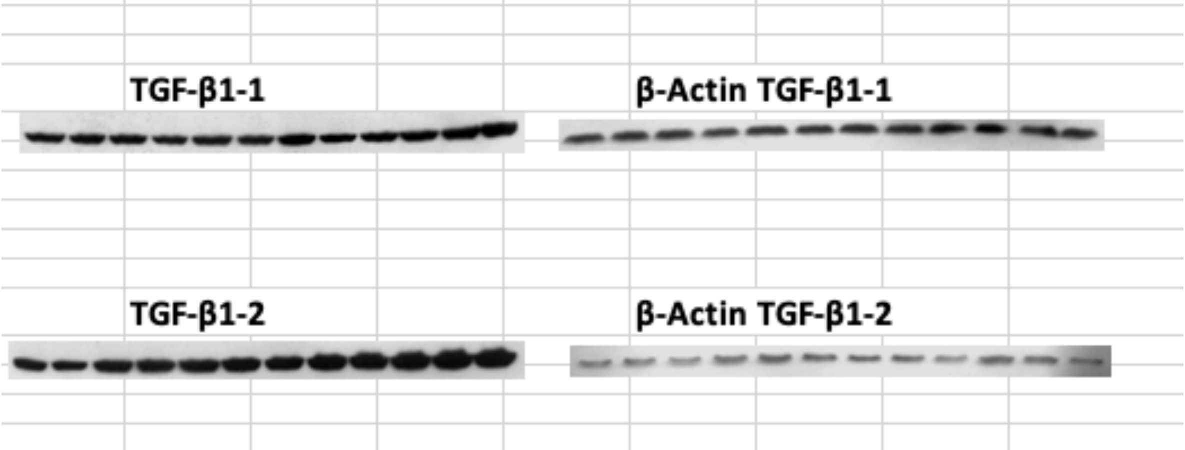

Group	TGF-β1-1	β-Actin TGF-β1-1	TGF-β1-2	β-Actin TGF-β1-2	TGF-β1-1/β-Actin	TGF-β1-2/β-Actin	Correction 1	Correction 2
Control	32.66846674	0.025225442	1.913327892	10.63887917	1295.06025	0.179842995	1	0.999999999
Control	33.54955067	0.025527994	1.931164474	10.42772924	1314.225896	0.185195111	1.014799038	1.029759936
Nx5/6 (2 days)	52.50905368	0.025629988	2.914410508	9.852136261	2048.734978	0.295815083	1.581961131	1.644851851
Nx5/6 (2 days)	51.99235916	0.025789065	2.988684439	9.936583867	2016.062224	0.300775848	1.556732379	1.672435715
Nx5/6 (4 days)	61.27003222	0.025042886	3.406170814	9.391819629	2446.604262	0.362674215	1.889181806	2.016615745
Nx5/6 (4 days)	59.49185482	0.025049983	3.520821455	9.456368138	2374.925941	0.372322799	1.833834326	2.070265785
Nx5/6 (7 days)	80.08071948	0.025895939	3.987195728	8.849002862	3092.404578	0.450581358	2.387846108	2.505415116
Nx5/6 (7 days)	81.62184219	0.025216517	4.06392778	8.885315737	3236.840423	0.457375731	2.499374391	2.543194584
Nx5/6 (14 days)	111.8362218	0.025899413	5.868292951	8.705846301	4318.098782	0.674063468	3.334284086	3.748066296
Nx5/6 (14 days)	110.8784873	0.025140859	5.520607627	8.256081541	4410.29035	0.668671645	3.405471174	3.718085574
Nx5/6 (28 days)	127.9371176	0.025565833	6.36995187	8.275973997	5004.2226	0.769692108	3.864084779	4.279800322
Nx5/6 (28 days)	129.5735831	0.026156189	6.451431009	8.457190949	4953.840384	0.762833788	3.825181403	4.241665282

α-SMA, β-Actin (Figure 1)

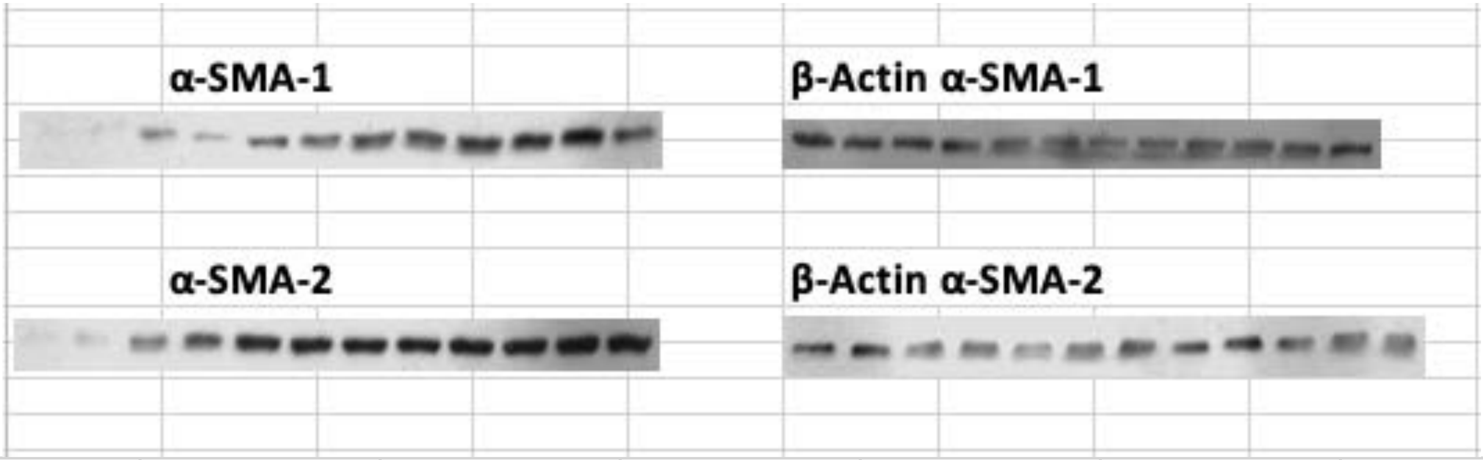

Group	α-SMA-1	β-Actin α-SMA-1	α-SMA-2	β-Actin α-SMA-2	α-SMA-1/β-Actin	α-SMA-2/β-Actin	Correction 1	Correction 2
Control	0.003182799	1.005355244	0.209077991	1.054218273	0.003165845	0.198325144	1.006204609	1.17573092
Control	0.003161997	1.004981506	0.177761989	1.053826369	0.003146323	0.168682426	1.000000088	1
Nx5/6 (2 days)	0.01451311	1.015295102	1.34341999	1.064641235	0.014294474	1.261852299	4.543231645	7.480638789
Nx5/6 (2 days)	0.01458818	1.032550695	1.209077991	1.082735497	0.014128294	1.116688235	4.490414347	6.620062689
Nx5/6 (4 days)	0.026095169	1.028654271	3.961575971	1.078649696	0.02536826	3.672717831	8.062827571	21.77297255
Nx5/6 (4 days)	0.025877333	0.999626023	4.298943967	1.048210595	0.025887014	4.101221631	8.227703898	24.31327156
Nx5/6 (7 days)	0.036814516	1.028654271	5.508021957	1.078649696	0.035789008	5.106404775	11.3748677	30.27229864
Nx5/6 (7 days)	0.036961943	1.033743478	5.10499596	1.083986252	0.035755431	4.709465594	11.36419581	27.91912416
Nx5/6 (14 days)	0.042767151	1.036500132	6.314073951	1.086876887	0.041261115	5.809373652	13.11407479	34.43970892
Nx5/6 (14 days)	0.044733556	1.022809634	6.179731952	1.072520994	0.043735955	5.761875046	13.90065652	34.15812295
Nx5/6 (28 days)	0.047621593	0.992705279	7.120125945	1.040953485	0.047971532	6.840003948	15.24685557	40.54959435
Nx5/6 (28 days)	0.043052733	1.013068574	7.523151941	1.062306491	0.042497353	7.08190339	13.50698999	41.98364678

VDAC, β-Actin (Figure 2)

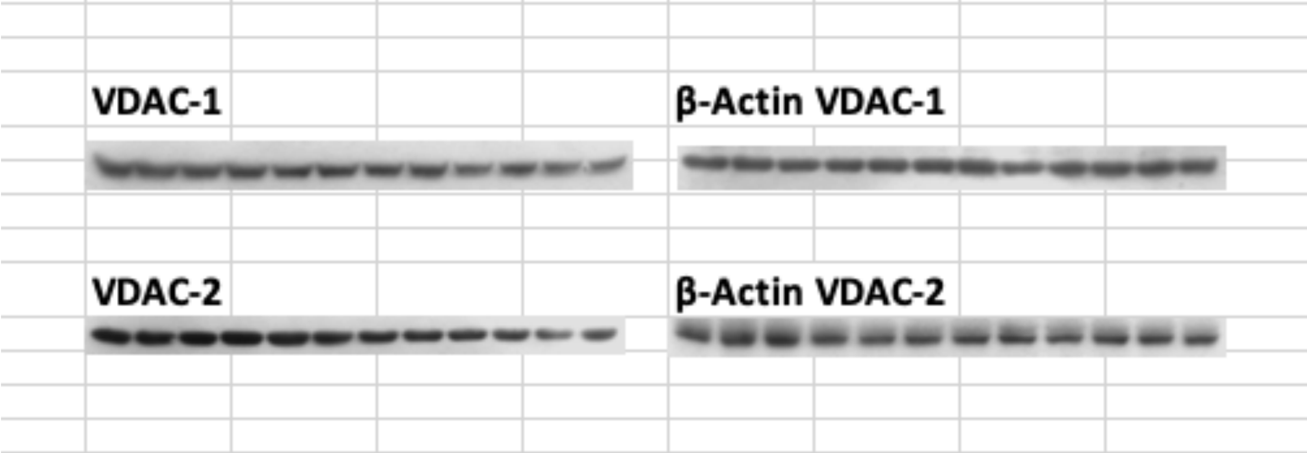

Group	VDAC-1	β-Actin VDAC-1	VDAC-2	β-Actin VDAC-2	VDAC-1/β-Actin	VDAC-2/β-Actin	Correction 1	Correction 2
Control	1.257822566	144.1434007	12.30043338	293.6137972	0.008726189	0.04189324	0.984218341	0.988943885
Control	1.263861699	142.5497338	12.30043338	290.3675675	0.008866111	0.042361595	0.999999949	1.000000007
Nx5/6 (2 days)	1.231615055	142.8602997	12.06532167	291.0001766	0.008621115	0.041461561	0.972367126	0.978753546
Nx5/6 (2 days)	1.244044723	142.2502595	12.12677136	289.7575515	0.008745465	0.041851442	0.9863925	0.987957182
Nx5/6 (4 days)	1.17556632	142.7796332	11.20721484	290.8358626	0.008233431	0.038534501	0.928640689	0.909656518
Nx5/6 (4 days)	1.142768199	143.6669644	11.70588105	292.6433173	0.007954287	0.040000507	0.897156208	0.944263477
Nx5/6 (7 days)	1.122192695	141.456199	10.37820928	288.1400849	0.007933146	0.03601793	0.894771802	0.850249609
Nx5/6 (7 days)	1.131555586	143.3150569	10.92109858	291.9264972	0.007895581	0.03741044	0.890534827	0.883121601
Nx5/6 (14 days)	0.877474309	142.1706014	8.33932133	289.5952914	0.006171981	0.028796467	0.696131755	0.679777692
Nx5/6 (14 days)	0.856752467	143.0216326	8.635545085	291.3288048	0.00599037	0.02964192	0.675647958	0.699735689
Nx5/6 (28 days)	0.851943688	143.0367576	8.136878867	291.3596137	0.005956117	0.027927271	0.671784638	0.659259193
Nx5/6 (28 days)	0.841775002	143.3921942	7.962103127	292.0836225	0.005870438	0.02725967	0.662120981	0.64349961

PGC-1α, β-Actin (Figure 2)

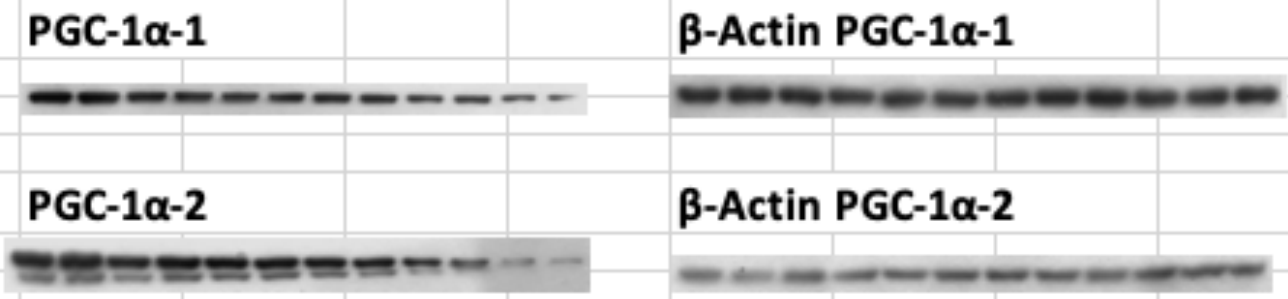

Group	PGC-1α-1	β-Actin PGC-1α-1	PGC-1α-2	β-Actin PGC-1α-2	PGC-1α-1/β-Actin	PGC-1α-2/β-Actin	Correction 1	Correction 2
Control	1.222467664	784.2063445	4.319690195	3883.945104	0.00155886	0.001112191	0.999999805	1.000000325
Control	1.180368681	775.536064	4.239805385	3841.003736	0.001522004	0.001103827	0.976356824	0.99248012
Nx5/6 (2 days)	1.059326625	777.2256853	4.010122361	3849.371937	0.001362959	0.00104176	0.874330504	0.936673799
Nx5/6 (2 days)	1.119226575	773.9067864	3.882871175	3832.934399	0.001446203	0.001013028	0.927731377	0.910840261
Nx5/6 (4 days)	0.993870809	776.7868226	3.592776676	3847.198378	0.001279464	0.000933868	0.820769051	0.839665415
Nx5/6 (4 days)	0.977453464	781.6143119	3.627612353	3871.107523	0.001250557	0.000937099	0.802225521	0.842570523
Nx5/6 (7 days)	0.922455471	769.5867321	3.37487683	3811.538431	0.001198637	0.000885437	0.768919237	0.796119475
Nx5/6 (7 days)	0.96336827	779.6997735	3.312407616	3861.625374	0.001235563	0.000857775	0.792606841	0.771248369
Nx5/6 (14 days)	0.5265434	773.4734095	2.999140998	3830.78801	0.000680752	0.000782904	0.436698472	0.70392986
Nx5/6 (14 days)	0.595709657	778.1034106	3.016534405	3853.719054	0.000765592	0.000782759	0.491122927	0.703799342
Nx5/6 (28 days)	0.491833416	778.1856974	1.925376771	3854.126596	0.000632026	0.000499562	0.405441009	0.449169621
Nx5/6 (28 days)	0.504671529	780.119436	1.832512688	3863.703839	0.000646916	0.000474289	0.41499283	0.426445744

NRF1, β-Actin (Figure 2)

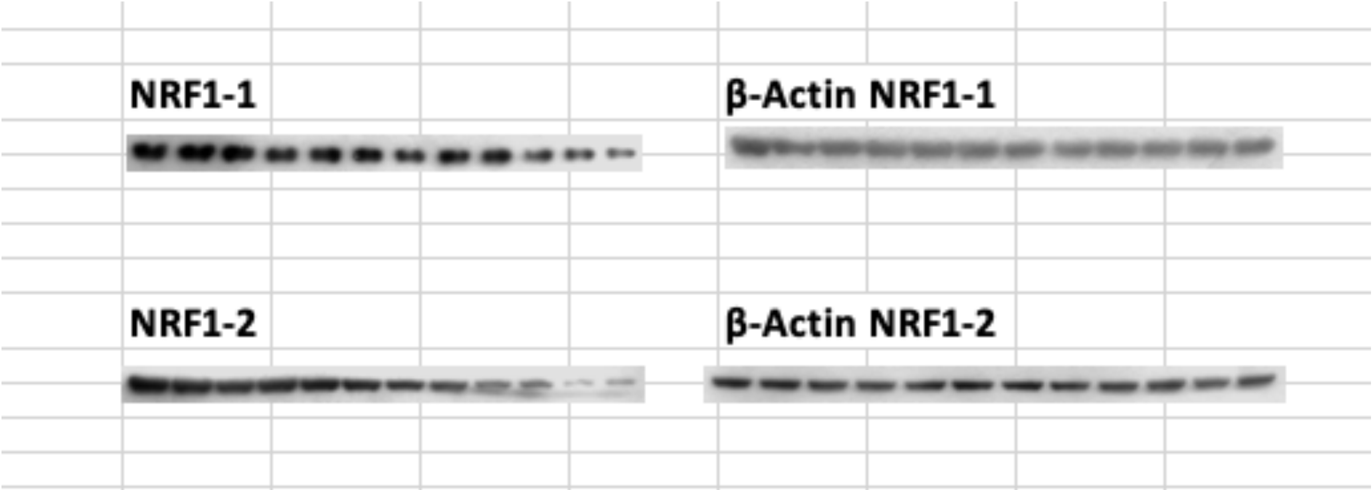

Group	NRF1-1	β-Actin NRF1-1	NRF1-2	β-Actin NRF1-2	NRF1-1/β-Actin	NRF1-2/β-Actin	Correction 1	Correction 2
Control	4.975327703	11908613.81	4.286904892	3535110.585	4.17792E-07	1.21267E-06	0.993513617	0.99999588
Control	5.005949667	11904186.81	4.158283558	3533796.415	4.2052E-07	1.17672E-06	1.000000205	0.970353421
Nx5/6 (2 days)	4.850173432	12026353.22	4.053209437	3570061.908	4.03295E-07	1.13533E-06	0.959039859	0.936225927
Nx5/6 (2 days)	4.831849317	12230748.82	4.3356759	3630737.405	3.95058E-07	1.19416E-06	0.939450018	0.984734916
Nx5/6 (4 days)	4.330563528	12184594.98	3.656435143	3622036.487	3.55413E-07	1.0095E-06	0.845175036	0.83245817
Nx5/6 (4 days)	4.206436196	12184594.98	3.581764704	3638036.487	3.45226E-07	9.84532E-07	0.820949708	0.811871635
Nx5/6 (7 days)	3.324577074	12184594.98	2.728087723	3619036.487	2.72851E-07	7.53816E-07	0.648841549	0.621616789
Nx5/6 (7 days)	3.20575962	12244877.55	2.714400841	3634931.564	2.61804E-07	7.46754E-07	0.622572383	0.615793512
Nx5/6 (14 days)	2.461651409	12277530.61	2.206031696	3644624.731	2.00501E-07	6.05284E-07	0.476791903	0.499133014
Nx5/6 (14 days)	2.502597972	12115364.21	2.076820379	3596485.109	2.06564E-07	5.77458E-07	0.491210858	0.47618755
Nx5/6 (28 days)	1.736040378	12249979.59	1.58247082	3636446.121	1.41718E-07	4.3517E-07	0.33700613	0.358852453
Nx5/6 (28 days)	1.724694109	11999979.59	1.497421758	3562232.812	1.43725E-07	4.2036E-07	0.34177864	0.346640423

NRF2, β-Actin (Figure 2)

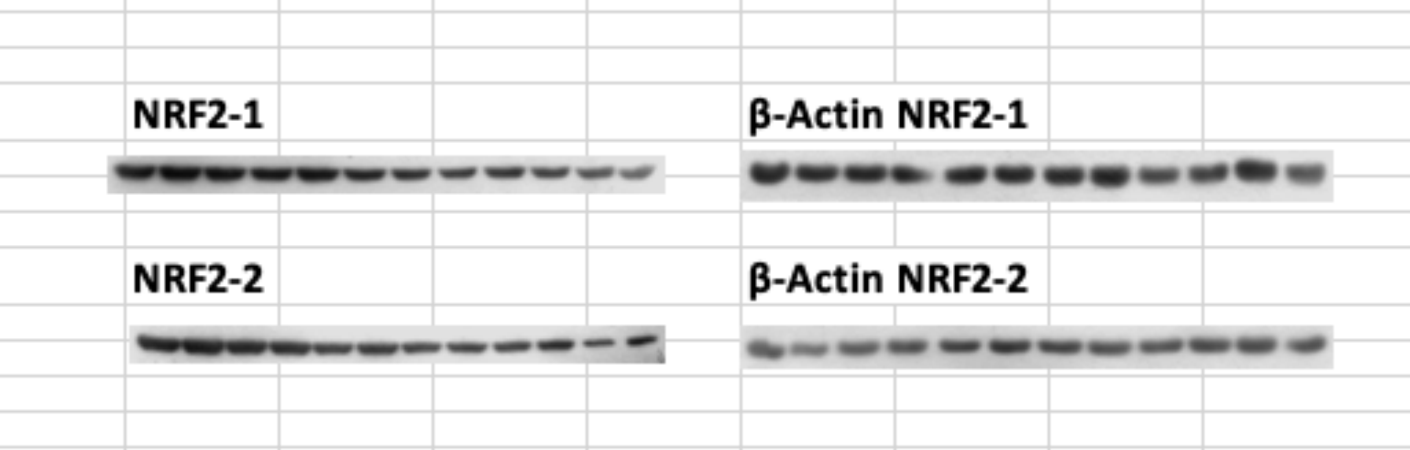

Group	NRF2-1	β-Actin NRF2-1	NRF2-2	β-Actin NRF2-2	NRF2-1/β-Actin	NRF2-2/β-Actin	Correction 1	Correction 2
Control	0.602390855	3.438922996	32.35804567	1.305125141	0.175168463	24.79305981	1.000390993	0.99139325
Control	0.581645896	3.400901846	32.27816086	1.290695519	0.171026958	25.00834658	0.976738764	1.000001863
Nx5/6 (2 days)	0.522000451	3.408311219	28.04847784	1.293507492	0.15315516	21.68404746	0.874672531	0.86707403
Nx5/6 (2 days)	0.551517127	3.393757093	28.92122665	1.287983973	0.162509311	22.45464793	0.928094295	0.897887818
Nx5/6 (4 days)	0.489746031	3.406386706	25.63113215	1.292777109	0.143772881	19.82641243	0.821090126	0.79279329
Nx5/6 (4 days)	0.481656117	3.427556344	25.66596783	1.300811318	0.140524639	19.7307384	0.802539342	0.788967599
Nx5/6 (7 days)	0.45455496	3.374812673	23.41323231	1.220794269	0.134690427	19.17868792	0.769220029	0.766892908
Nx5/6 (7 days)	0.474715408	3.419160658	23.35076309	1.207625024	0.138839749	19.33610403	0.7929169	0.773187463
Nx5/6 (14 days)	0.259462838	2.491856637	20.03749648	1.28726272	0.104124304	15.56597279	0.594656218	0.622432264
Nx5/6 (14 days)	0.293545638	2.712160244	20.05488988	1.294968257	0.108233147	15.48678106	0.618121912	0.619265646
Nx5/6 (28 days)	0.242358928	2.41252109	17.96373225	1.295105204	0.100458781	13.8704811	0.573722336	0.554635105
Nx5/6 (28 days)	0.248685117	2.571000973	17.87086817	1.298323452	0.096726963	13.76457318	0.552409843	0.550400194

TFAM, β-Actin (Figure 2)

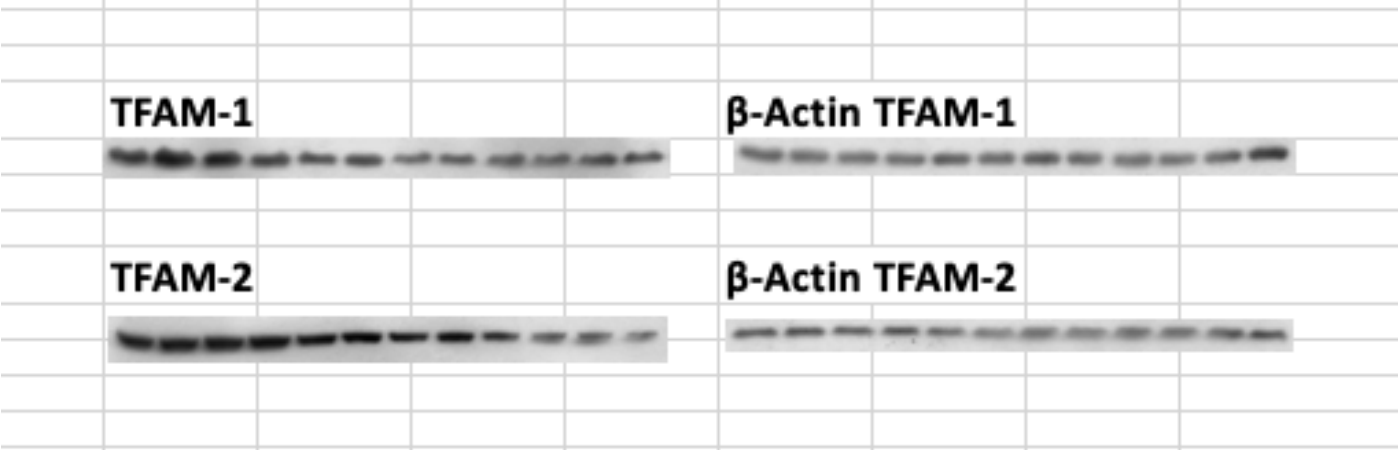

Group	TFAM-1	β-Actin TFAM-1	TFAM-2	β-Actin TFAM-2	TFAM-1/β-Actin	TFAM-2/β-Actin	Correction 1	Correction 2
Control	0.379356608	314.2230507	0.281295283	135.3741581	0.001207284	0.00207791	0.960478859	0.960299169
Control	0.399703207	317.9918211	0.287110902	132.6873859	0.001256961	0.002163815	0.999999722	0.999999814
Nx5/6 (2 days)	0.303814893	319.2623248	0.199008131	125.3632671	0.000951615	0.001587452	0.757076183	0.73363559
Nx5/6 (2 days)	0.299623009	321.2438738	0.19339125	126.4378186	0.000932696	0.001529536	0.742024942	0.70687024
Nx5/6 (4 days)	0.249349655	311.9490327	0.158828692	119.5059794	0.000799328	0.001329044	0.635921233	0.614213273
Nx5/6 (4 days)	0.253536278	312.0374359	0.161835986	120.3273253	0.000812519	0.001344965	0.646415269	0.621570948
Nx5/6 (7 days)	0.214149406	322.57516	0.134107426	112.5989207	0.000663874	0.001191019	0.52815835	0.550425405
Nx5/6 (7 days)	0.2093188	314.1118826	0.131905142	113.060983	0.000666383	0.001166673	0.53015403	0.539173877
Nx5/6 (14 days)	0.226971683	322.6184319	0.126134696	110.7773285	0.00070353	0.001138633	0.55970695	0.526215395
Nx5/6 (14 days)	0.212935132	313.1694377	0.122661553	105.0543078	0.000679936	0.001167601	0.540936324	0.539603137
Nx5/6 (28 days)	0.211876744	318.4631631	0.122402426	105.3074289	0.00066531	0.001162334	0.529300473	0.537168933
Nx5/6 (28 days)	0.223880077	325.8169857	0.117518155	107.6133196	0.000687134	0.001092041	0.546663306	0.504683156

CPT1, β-Actin (Figure 2)

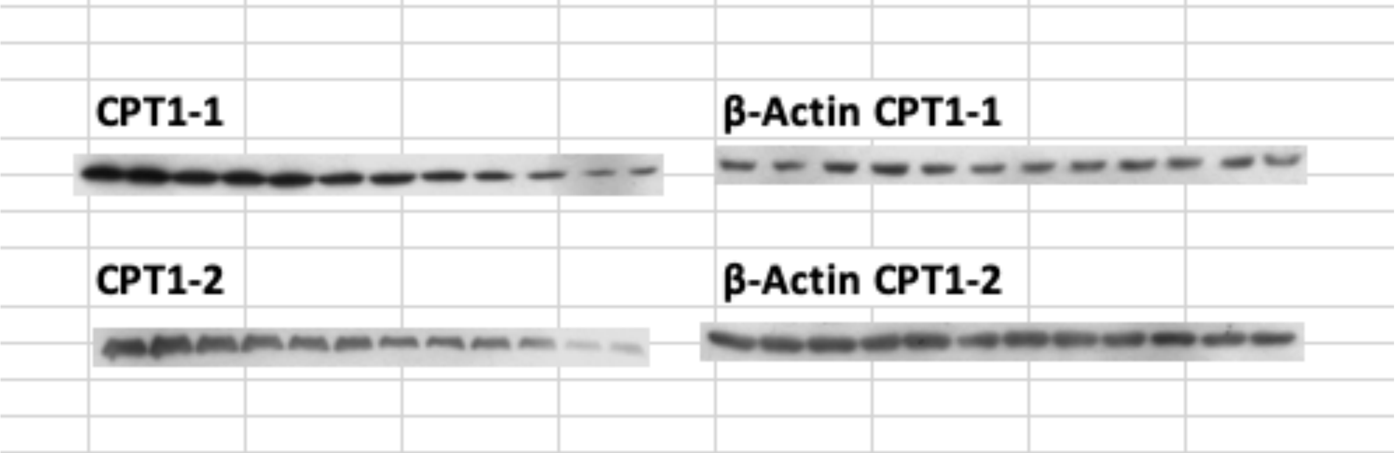

Group	CPT1-1	β-Actin CPT1-1	CPT1-2	β-Actin CPT1-2	CPT1-1/β-Actin	CPT1-2/β-Actin	Correction 1	Correction 2
Control	3.387150955	1.059121675	3.993676523	9.53510176	3.198075381	0.418839423	0.960479126	0.960299347
Control	3.568819068	1.07182471	4.076243512	9.345858505	3.329666721	0.436155064	1	1
Nx5/6 (2 days)	2.712663707	1.076107076	2.825408562	8.82998296	2.520812071	0.319978937	0.757076393	0.733635726
Nx5/6 (2 days)	2.675235751	1.082786094	2.745663156	8.905669177	2.470696442	0.308305092	0.742025148	0.706870372
Nx5/6 (4 days)	2.226361434	1.05145686	2.254962875	8.417423907	2.117406353	0.267892279	0.635921409	0.614213387
Nx5/6 (4 days)	2.263742415	1.051754832	2.297658786	8.475275546	2.152348006	0.271101367	0.646415448	0.621571064
Nx5/6 (7 days)	1.912069936	1.087273334	1.903983856	7.930924058	1.75859177	0.240070872	0.528158497	0.550425507
Nx5/6 (7 days)	1.868939032	1.058746972	1.872717043	7.963469493	1.765236721	0.23516346	0.530154177	0.539173977
Nx5/6 (14 days)	1.202655585	1.087419186	1.17907914	7.802619904	1.10597238	0.151113236	0.332157081	0.346466769
Nx5/6 (14 days)	1.190122808	1.055570362	1.174148162	7.399517972	1.12746895	0.158679007	0.338613154	0.363813285
Nx5/6 (28 days)	0.891778074	1.073413418	0.737802671	7.417346598	0.83078715	0.099469893	0.249510603	0.228060846
Nx5/6 (28 days)	0.898951912	1.098200247	0.768458476	7.579762399	0.818568303	0.101382924	0.245840912	0.232446972

PPARα, β-Actin (Figure 2)

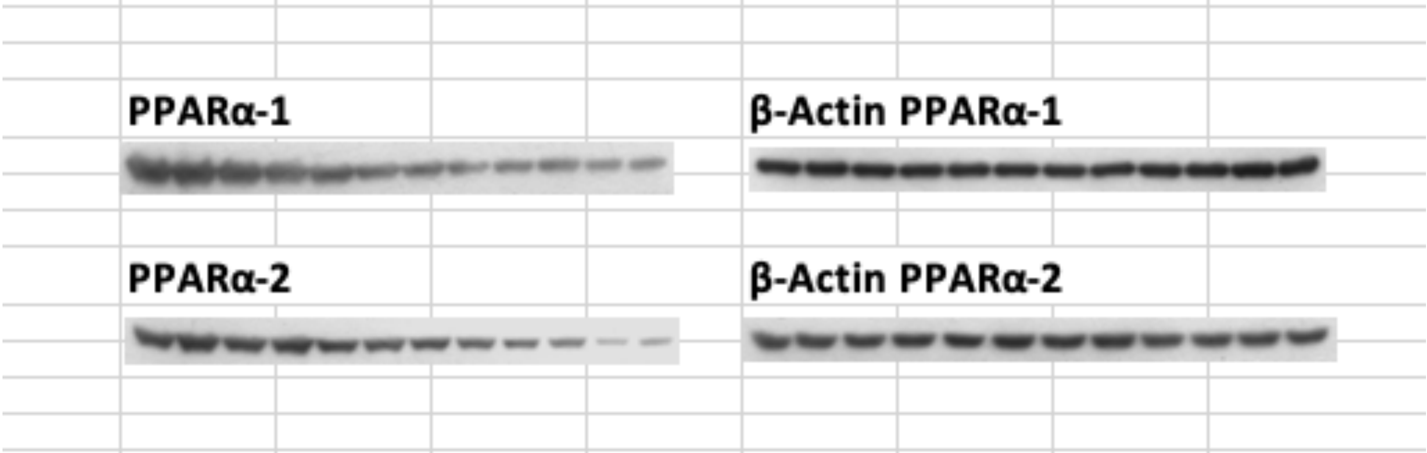

Group	PPARα-1	β-Actin PPARα-1	PPARα-2	β-Actin PPARα-2	PPARα-1/β-Actin	PPARα-2/β-Actin	Correction 1	Correction 2
Control	0.218574551	0.056223613	2.068487118	1.935015028	3.887593497	1.068977289	0.999227239	0.968188832
Control	0.221370376	0.056897955	2.094083858	1.896610766	3.890656096	1.104118934	1.000014418	1.000017149
Nx5/6 (2 days)	0.191066928	0.057125285	1.695427582	1.791921067	3.344699773	0.946150817	0.859687393	0.856943046
Nx5/6 (2 days)	0.189430683	0.057479842	1.699721877	1.807280522	3.295602044	0.940485916	0.847067816	0.851812259
Nx5/6 (4 days)	0.136814145	0.055816725	1.208667662	1.708198	2.451131712	0.707568831	0.630013806	0.640855748
Nx5/6 (4 days)	0.139447081	0.055832543	1.214119632	1.719938177	2.497595008	0.70590888	0.641956256	0.639352305
Nx5/6 (7 days)	0.10467697	0.057718047	0.832828272	1.609469686	1.813591673	0.517455084	0.46614704	0.468666864
Nx5/6 (7 days)	0.101639037	0.056203722	0.833222002	1.616074325	1.808404043	0.515583961	0.464813665	0.466972159
Nx5/6 (14 days)	0.08970925	0.05772579	0.69134714	1.583432159	1.554058422	0.436613047	0.39943927	0.395447013
Nx5/6 (14 days)	0.088826502	0.056035091	0.670942956	1.501628282	1.58519421	0.446810282	0.407442094	0.404682802
Nx5/6 (28 days)	0.05581254	0.056982292	0.428232549	1.505246351	0.979471657	0.284493331	0.251753369	0.257669895
Nx5/6 (28 days)	0.05731783	0.058298104	0.429804725	1.538206357	0.983185154	0.279419418	0.252707848	0.253074375

DRP1, VDAC (Figure 3)

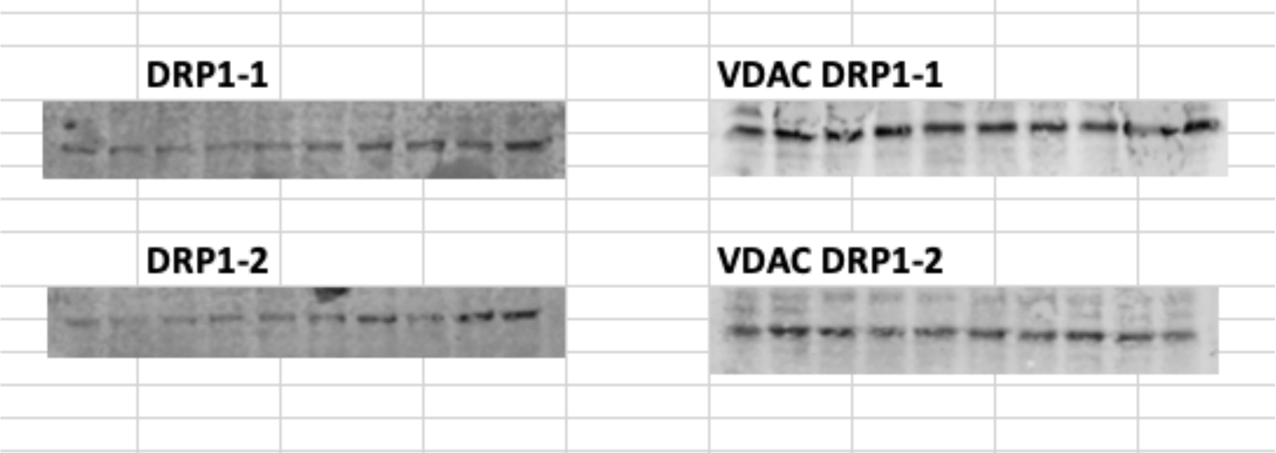

Group	DRP1-1	VDAC DRP1-1	DRP1-2	VDAC DRP1-2	DRP1-1/VDAC	DRP1-2/VDAC	Correction 1	Correction 2
Control	78.40236686	357.6388889	92.70833333	345.7142857	0.219222152	0.268164601	1.067618313	1.042507223
Control	78.63247863	410.7142857	91.48148148	371.4285714	0.191452991	0.246296296	0.932381687	0.957492777
Nx5/6 (2 days)	76.38888889	364.1025641	92.22222222	363.9053254	0.209800469	0.253423668	1.021734443	0.985200893
Nx5/6 (2 days)	71.67832168	407.0512821	91.98717949	331.3609467	0.176091625	0.277604167	0.857571381	1.079204147
Nx5/6 (7 days)	72.8	394.984326	93.70629371	326.1904762	0.184311111	0.287274769	0.89760052	1.116799239
Nx5/6 (7 days)	67.85714286	376.4367816	92.02279202	355.0295858	0.180261723	0.259197531	0.877879881	1.00764716
Nx5/6 (14 days)	78.67132867	324.0740741	98.35164835	321.4285714	0.242757243	0.305982906	1.182234896	1.189528331
Nx5/6 (14 days)	77.16049383	348.7654321	92.03296703	351.0971787	0.221238938	0.262129611	1.077440121	1.019045813
Nx5/6 (28 days)	81.36094675	361.7463617	107.6923077	315.3846154	0.224911583	0.341463415	1.095326008	1.327461102
Nx5/6 (28 days)	106.2962963	396.9230769	117.0940171	298.816568	0.267800746	0.391859186	1.304197494	1.523377922

FIS1, VDAC (Figure 3)

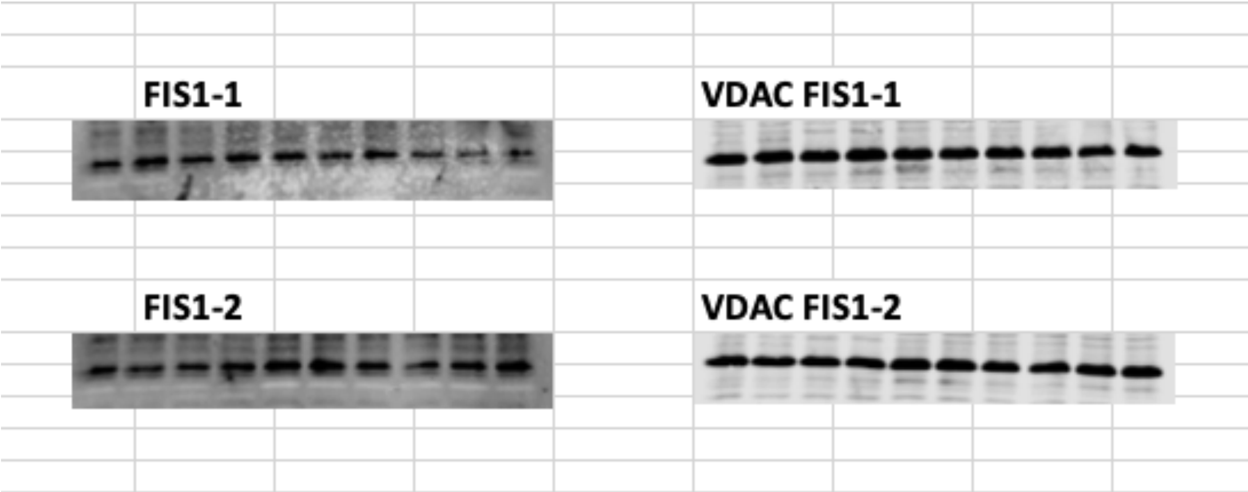

Group	FIS1-1	VDAC FIS1-1	FIS1-2	VDAC FIS1-2	FIS1-1/VDAC	FIS1-2/VDAC	Correction 1	Correction 2
Control	900	3479.020979	976	3562.962963	0.258693467	0.273929314	1.091952171	0.975560173
Control	987.9807692	4592.592593	932	3240	0.215124845	0.287654321	0.908047829	1.024439827
Nx5/6 (2 days)	992.3076923	4185.185185	924	3651.851852	0.237100068	0.253022312	1.000805845	0.901102869
Nx5/6 (2 days)	891.2037037	5142.857143	1060	4037.037037	0.173289609	0.262568807	0.731460159	0.935101349
Nx5/6 (7 days)	868.1318681	4370.37037	1292	5071.428571	0.198640343	0.254760563	0.838466296	0.907293402
Nx5/6 (7 days)	814.8148148	4074.074074	1288	4888.888889	0.2	0.263454545	0.844205447	0.938255779
Nx5/6 (14 days)	1011.363636	4786.324786	948	3640	0.21130276	0.26043956	0.891914703	0.92751834
Nx5/6 (14 days)	801.7241379	3740.740741	860	3596.296296	0.214322294	0.239134912	0.904660241	0.851644876
Nx5/6 (28 days)	685.8974359	2541.818182	1068	4653.846154	0.2698452	0.229487603	1.139023939	0.81728736
Nx5/6 (28 days)	909.0909091	4391.304348	1196	4555.555556	0.207020702	0.262536585	0.873840021	0.934986595

MFN1, VDAC (Figure 3)

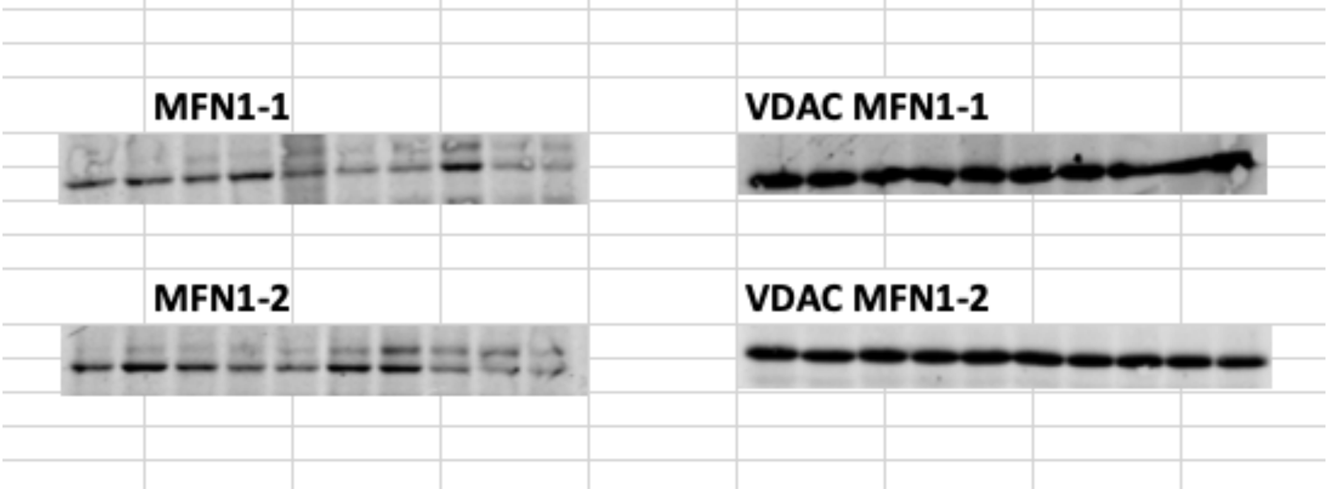

Group	MFN1-1	VDAC MFN1-1	MFN1-2	VDAC MFN1-2	MFN1-1/VDAC	MFN1-2/VDAC	Correction 1	Correction 2
Control	223.3333333	596.6735967	219.2592593	535.5555556	0.374297329	0.409405256	1.023418897	0.859726466
Control	230	643.956044	295.6349206	544.4444444	0.357167235	0.543002915	0.976581103	1.140273534
Nx5/6 (2 days)	219.6153846	649.867374	215.7142857	562.2222222	0.337938776	0.383681536	0.92400587	0.805708198
Nx5/6 (2 days)	252.4137931	809.0909091	191.958042	544.4444444	0.311972104	0.352575995	0.853006746	0.74038843
Nx5/6 (7 days)	244.6428571	727.2727273	183.7931034	593.3333333	0.336383929	0.309763657	0.919754545	0.65048509
Nx5/6 (7 days)	182.7586207	712.1212121	237.5862069	602.2222222	0.256639765	0.394515842	0.701714827	0.82845959
Nx5/6 (14 days)	204.5833333	745.4545455	240.7142857	528.8888889	0.274441057	0.455132053	0.750387839	0.955749996
Nx5/6 (14 days)	268.5714286	639.3939394	175.5952381	542.2222222	0.420040623	0.323843677	1.148492062	0.680052285
Nx5/6 (28 days)	184.6153846	757.5757576	180.4347826	508.8888889	0.243692308	0.354566167	0.666313365	0.74456767
Nx5/6 (28 days)	182.9710145	896.969697	170.5685619	462.2222222	0.203987955	0.369018523	0.557752119	0.774916752

MFN2, VDAC (Figure 3)

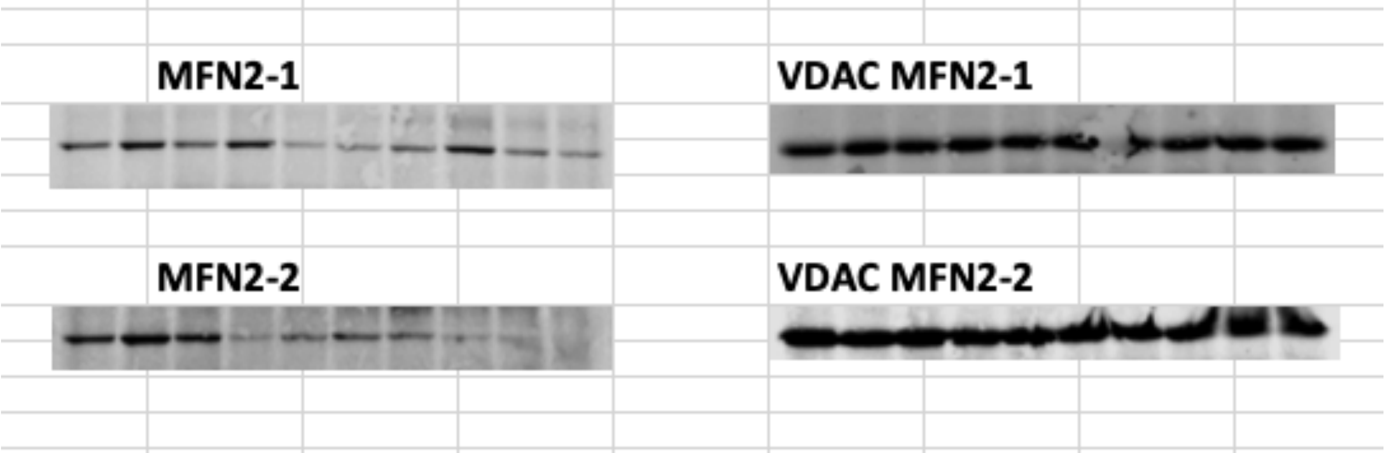

Group	MFN2-1	VDAC MFN2-1	MFN2-2	VDAC MFN2-2	MFN2-1/VDAC	MFN2-2/VDAC	Correction 1	Correction 2
Control	195.6989247	448.9795918	184.0740741	569.124424	0.435874878	0.323433798	0.950545331	0.684663701
Control	269.4444444	559.9078341	366.5178571	589.8617512	0.481229995	0.621362305	1.049454669	1.315336299
Nx5/6 (2 days)	190.0862069	534.3915344	195.6349206	550.6912442	0.355705872	0.355253371	0.775714714	0.752021244
Nx5/6 (2 days)	247.5	589.9470899	122.7678571	421.6589862	0.419529148	0.291154372	0.914898961	0.616332711
Nx5/6 (7 days)	138.8888889	566.1375661	133.6666667	470.0460829	0.245327103	0.284369281	0.535003378	0.601969632
Nx5/6 (7 days)	154.589372	584.6560847	147.5095785	435.483871	0.264410781	0.338725699	0.5766206	0.717034496
Nx5/6 (14 days)	177.5	444.4444444	137.164751	426.2672811	0.399375	0.321781091	0.870947286	0.681165154
Nx5/6 (14 days)	276.6666667	542.3280423	112.4668435	382.4884793	0.510146341	0.29403982	1.112514733	0.622440797
Nx5/6 (28 days)	171.5517241	624.3386243	116.5680473	336.40553	0.274773524	0.346510497	0.599219418	0.733513815
Nx5/6 (28 days)	163.8888889	589.2857143	111.1111111	301.843318	0.278114478	0.368108567	0.606505289	0.77923388

OPA1, VDAC (Figure 3)

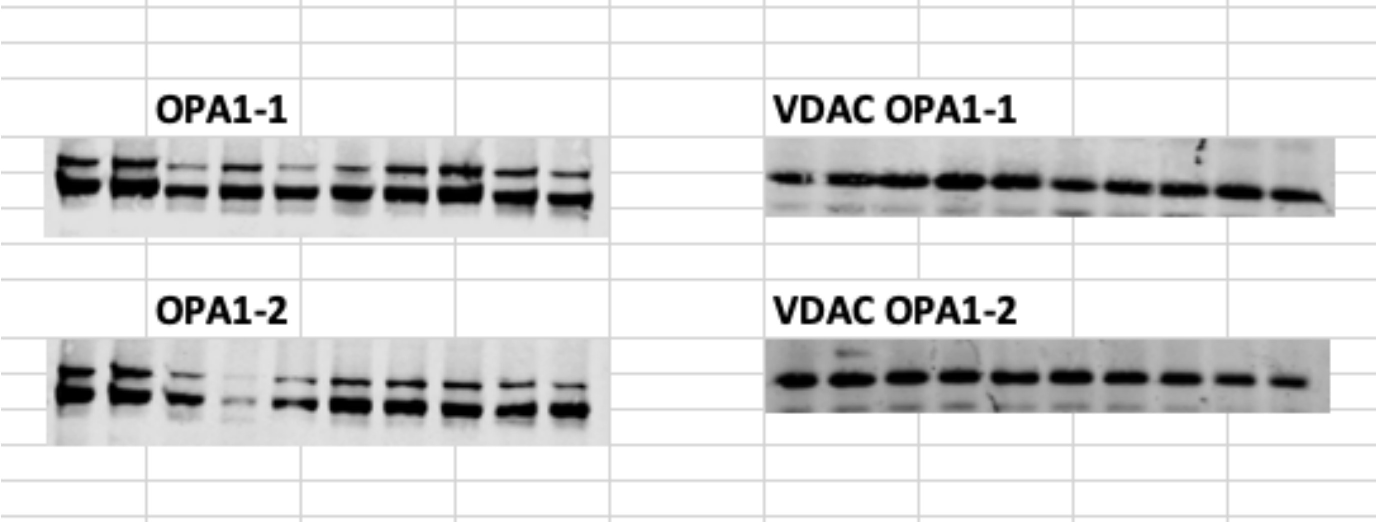

Group	OPA1-1	VDAC OPA1-1	OPA1-2	VDAC OPA1-2	OPA1-1/VDAC	OPA1-2/VDAC	Correction 1	Correction 2
Control	324.5	720	0.763888889	635.4166667	0.450694444	0.001202186	1.06118663	1.029292666
Control	347.6851852	872	0.776699029	685.0649351	0.398721543	0.00113376	0.93881337	0.970707334
Nx5/6 (2 days)	121.8106996	1064	0.383211679	583.3333333	0.11448374	0.000656934	0.269558713	0.562456875
Nx5/6 (2 days)	198.5576923	1432	0.123214286	570.5128205	0.138657606	0.000215971	0.326477506	0.184911083
Nx5/6 (7 days)	115.3846154	1128	0.450381679	539.3939394	0.102291326	0.000834977	0.240850955	0.714894475
Nx5/6 (7 days)	150.9615385	824	0.614068441	547.6190476	0.183205751	0.001121342	0.431368737	0.960075793
Nx5/6 (14 days)	243.9814815	956	0.687037037	491.0714286	0.255210755	0.001399057	0.600908762	1.197850919
Nx5/6 (14 days)	353.4188034	912	0.644194757	462.962963	0.387520618	0.001391461	0.912440132	1.191346859
Nx5/6 (28 days)	233.1730769	1048	0.587078652	401.2345679	0.222493394	0.001463181	0.523873808	1.252752371
Nx5/6 (28 days)	149.1452991	876	0.521172638	414.5454545	0.170257191	0.001257215	0.400880589	1.076407554

PINK1, VDAC (Figure 5)

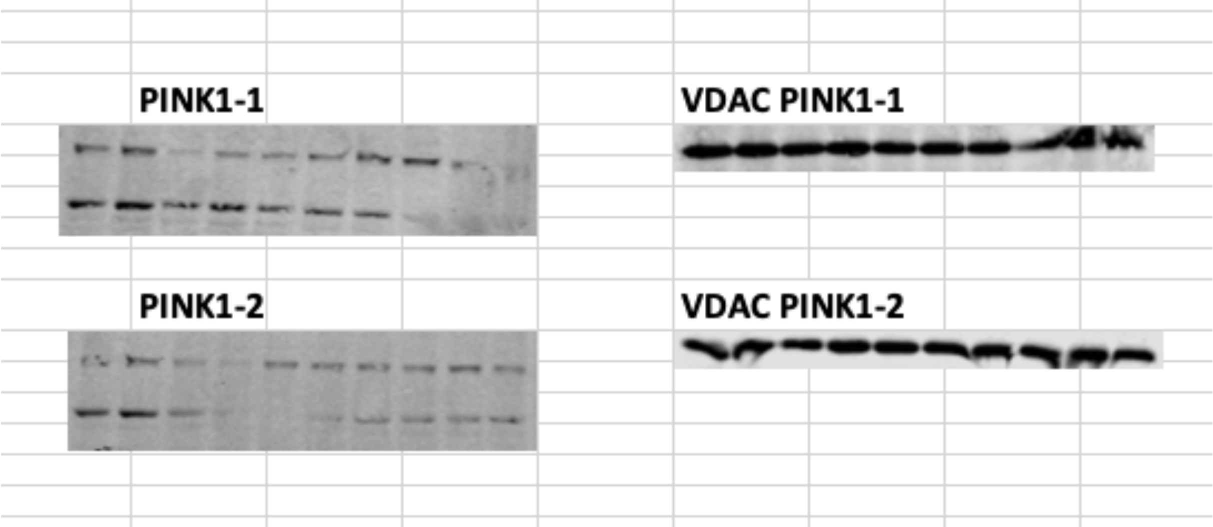

Group	PINK1-1	VDAC PINK1-1	PINK1-2	VDAC PINK1-2	PINK1-1/VDAC	PINK1-2/VDAC	Correction 1	Correction 2
Control	78.84615385	826.1904762	72.85714286	520.3761755	0.095433385	0.140008606	0.982976543	0.9883391
Control	82.51748252	835.7142857	80.41666667	561.1285266	0.098738868	0.143312384	1.017023457	1.0116609
Nx5/6 (2 days)	61.27946128	750	66.02564103	526.645768	0.081705948	0.125370116	0.841582122	0.885004081
Nx5/6 (2 days)	67.003367	830.6451613	62.58741259	708.4639498	0.080664248	0.08834241	0.830852466	0.62362065
Nx5/6 (7 days)	66.32996633	723.1182796	71.53846154	699.0595611	0.091727686	0.102335288	0.944807349	0.722398211
Nx5/6 (7 days)	70.37037037	739.2473118	71.63636364	664.5768025	0.095191919	0.107792453	0.980489413	0.760921053
Nx5/6 (14 days)	73.58974359	693.5483871	70.54545455	633.2288401	0.106106142	0.111405941	1.092907357	0.786429137
Nx5/6 (14 days)	81.11111111	481.1827957	70.90909091	567.3981191	0.168566108	0.124972376	1.736253305	0.88219638
Nx5/6 (28 days)	66.08391608	684.2105263	79.11111111	608.1504702	0.096584185	0.130084765	0.994829936	0.918285408
Nx5/6 (28 days)	68.88888889	556.4516129	70.83333333	523.5109718	0.123800322	0.135304391	1.275159763	0.955131432

Parkin, VDAC (Figure 5)

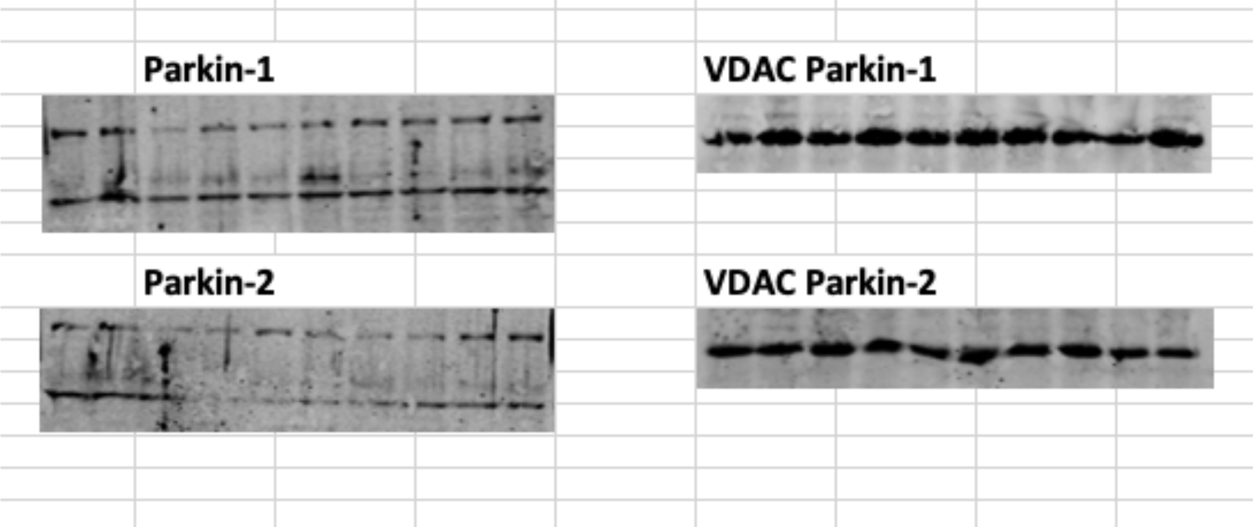

Group	Parkin-1	VDAC Parkin-1	Parkin-2	VDAC Parkin-2	Parkin-1/VDAC	Parkin-2/VDAC	Correction 1	Correction 2
Control	126	784.1191067	108.8	600	0.160689873	0.181333333	1.244624616	0.968018721
Control	116.4	1193.548387	117.6	608.3333333	0.097524324	0.193315068	0.755375384	1.031981279
Nx5/6 (2 days)	86.4	1024.813896	97.6	683.3333333	0.08430799	0.142829268	0.65300817	0.762470987
Nx5/6 (2 days)	99.2	1300.248139	99.2	561.1111111	0.07629313	0.176792079	0.590929007	0.943775969
Nx5/6 (7 days)	92.8	1012.406948	97.2	522.2222222	0.091662745	0.18612766	0.709974478	0.993612457
Nx5/6 (7 days)	99.2	1106.699752	96	566.6666667	0.089635874	0.169411765	0.694275336	0.904377352
Nx5/6 (14 days)	105.6	1203.473945	91.6	577.7777778	0.087745979	0.158538462	0.67963714	0.846331978
Nx5/6 (14 days)	104	962.7791563	94.4	658.3333333	0.108020619	0.143392405	0.836674509	0.765477201
Nx5/6 (28 days)	106.4	873.4491315	109.6	511.1111111	0.121815909	0.214434783	0.943526035	1.144725463
Nx5/6 (28 days)	113.6	1540.942928	113.6	452.7777778	0.073721095	0.250895706	0.571007293	1.339366213

p62, β-Actin (Figure 5)

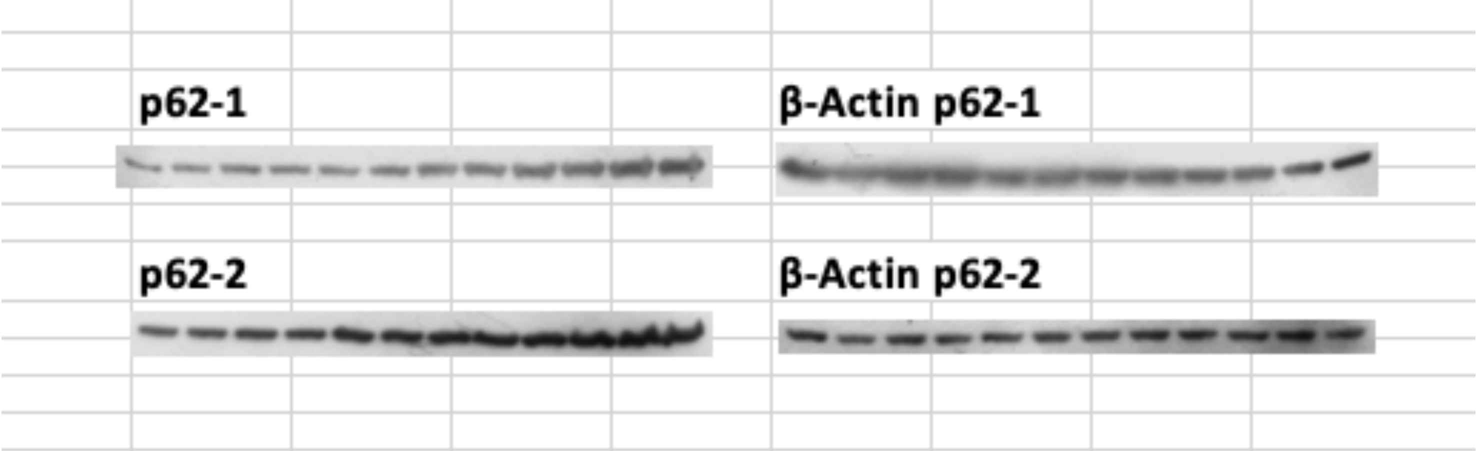

Group	p62-1	β-Actin p62-1	p62-2	β-Actin p62-2	p62-1/β-Actin	p62-2/β-Actin	Correction 1	Correction 2
Control	0.012021369	6.377000087	2.21886875	293.6137972	0.001885113	0.0075571	1.006204698	0.999999965
Control	0.0119428	6.374629451	2.199616815	290.3675675	0.001873489	0.007575284	1.000000176	1.002406226
Nx5/6 (2 days)	0.017045928	6.440048918	3.093197504	291.0001766	0.002646863	0.010629538	1.412798836	1.406563131
Nx5/6 (2 days)	0.017329466	6.54950169	3.092023261	289.7575515	0.002645921	0.010671071	1.412296137	1.412058925
Nx5/6 (4 days)	0.023021334	6.524786548	4.195200768	290.8358626	0.003528289	0.014424634	1.883271952	1.908752517
Nx5/6 (4 days)	0.022198572	6.340659454	4.220600964	292.6433173	0.003500988	0.014422338	1.86869949	1.908448802
Nx5/6 (7 days)	0.028998738	6.524786548	5.22688738	288.1400849	0.004444396	0.018140091	2.37225647	2.400403735
Nx5/6 (7 days)	0.029127848	6.55706755	5.291878785	291.9264972	0.004442206	0.018127436	2.371087547	2.398729093
Nx5/6 (14 days)	0.032985464	6.574553092	5.983226283	289.5952914	0.005017142	0.020660648	2.677966981	2.733938637
Nx5/6 (14 days)	0.032458577	6.487713835	6.009287935	291.3288048	0.005003084	0.020627167	2.670463511	2.729508289
Nx5/6 (28 days)	0.040117552	6.29676096	7.732773442	291.3596137	0.006371141	0.026540306	3.400682408	3.511969734
Nx5/6 (28 days)	0.041745959	6.42592598	7.552426446	292.0836225	0.006496489	0.025857069	3.467588636	3.421559755

LC3B I/II, β-Actin (Figure 5)

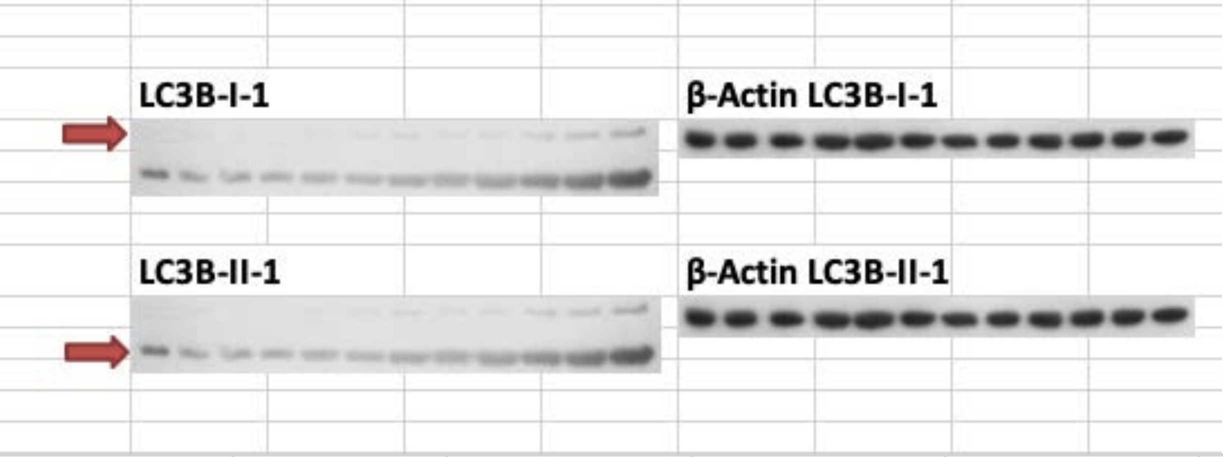

Group	LC3B-I-1	β-Actin LC3B-I-1	LC3B-I-1/β-Actin
Control	9	48.48716139	0.185616145
Control	11	48.46913639	0.226948545
Nx5/6 (2 days)	10	48.96654963	0.204221046
Nx5/6 (2 days)	9	49.79876762	0.180727364
Nx5/6 (4 days)	28	49.61084743	0.564392697
Nx5/6 (4 days)	32	48.21084743	0.663751037
Nx5/6 (7 days)	41	49.61084743	0.826432164
Nx5/6 (7 days)	38	49.85629421	0.762190624
Nx5/6 (14 days)	47	49.98924455	0.940202246
Nx5/6 (14 days)	46	49.32896715	0.932514964
Nx5/6 (28 days)	53	47.8770677	1.107001797
Nx5/6 (28 days)	56	48.85916667	1.146151353

Group	LC3B-II-1	β-Actin LC3B-II-1	LC3B-II-1/β-Actin
Control	0.848750016	48.48716139	0.017504634
Control	0.843202826	48.46913639	0.017396696
Nx5/6 (2 days)	1.203501197	48.96654963	0.024578027
Nx5/6 (2 days)	1.223519969	49.79876762	0.024569282
Nx5/6 (4 days)	1.625385493	49.61084743	0.032762704
Nx5/6 (4 days)	1.567295648	48.21084743	0.032509191
Nx5/6 (7 days)	1.817212198	49.61084743	0.036629332
Nx5/6 (7 days)	1.856526354	49.85629421	0.037237552
Nx5/6 (14 days)	2.071249443	49.98924455	0.041433902
Nx5/6 (14 days)	2.062290785	49.32896715	0.041806892
Nx5/6 (28 days)	2.832437357	47.8770677	0.059160627
Nx5/6 (28 days)	2.947408433	48.85916667	0.060324574

LC3B I/II, β-Actin (Figure 5)

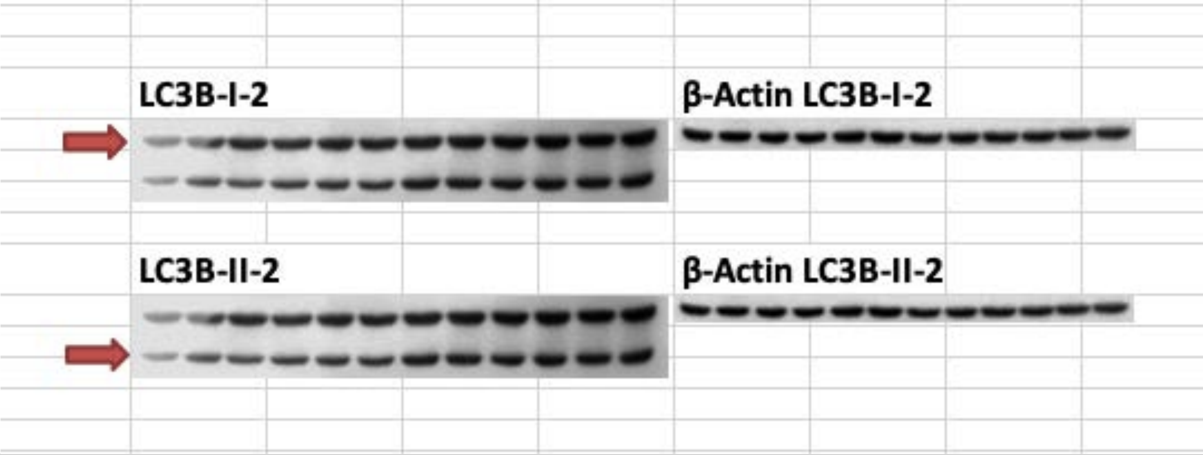

Group	LC3B-I-2	β-Actin LC3B-I-2	LC3B-I-2/β-Actin
Control	21.95151502	1.479672599	14.83538658
Control	21.80804612	1.479122534	14.74390771
Nx5/6 (2 days)	31.12656742	1.49430199	20.83017197
Nx5/6 (2 days)	31.64431984	1.519698613	20.82276023
Nx5/6 (4 days)	42.03790677	1.513963891	27.76678295
Nx5/6 (4 days)	40.53550904	1.471240383	27.55192795
Nx5/6 (7 days)	46.99918715	1.513963891	31.04379663
Nx5/6 (7 days)	48.01598276	1.52145414	31.55927051
Nx5/6 (14 days)	53.56944021	1.525511357	35.1157269
Nx5/6 (14 days)	53.33773933	1.505361809	35.43184037
Nx5/6 (28 days)	73.25630632	1.461054497	50.13933874
Nx5/6 (28 days)	76.22984299	1.491025006	51.12579781

Group	LC3B-II-2	β-Actin LC3B-II-2	LC3B-II-2/β-Actin
Control	30	1.479672599	20.27475539
Control	30	1.479122534	20.28229529
Nx5/6 (2 days)	31	1.49430199	20.74547194
Nx5/6 (2 days)	32.32	1.519698613	21.26737481
Nx5/6 (4 days)	34	1.513963891	22.45760298
Nx5/6 (4 days)	33.9	1.471240383	23.04178188
Nx5/6 (7 days)	40.28	1.513963891	26.60565435
Nx5/6 (7 days)	42.23	1.52145414	27.75634106
Nx5/6 (14 days)	44.52	1.525511357	29.18365687
Nx5/6 (14 days)	44.41	1.505361809	29.50121341
Nx5/6 (28 days)	46.42	1.461054497	31.77157327
Nx5/6 (28 days)	46.34	1.491025006	31.07929096

LC3B I/II, β-Actin (Figure 5)

Correction LC3B-I				
Group	LC3B-I-2/β-Actin LC3B-I-2	LC3B-I-1/β-Actin LC3B-I-1	Correction 1	Correction 2
Control	14.83538658	0.185616145	1.00620452	1.000000002
Control	14.74390771	0.226948545	1	1.222676752
Nx5/6 (2 days)	20.83017197	0.204221046	1.412798586	1.100233206
Nx5/6 (2 days)	20.82276023	0.180727364	1.412295888	0.973661876
Nx5/6 (4 days)	27.76678295	0.564392697	1.88327162	3.04064443
Nx5/6 (4 days)	27.55192795	0.663751037	1.86869916	3.57593375
Nx5/6 (7 days)	31.04379663	0.826432164	2.105533841	4.4523722
Nx5/6 (7 days)	31.55927051	0.762190624	2.140495663	4.10627332
Nx5/6 (14 days)	35.1157269	0.940202246	2.381710981	5.065304238
Nx5/6 (14 days)	35.43184037	0.932514964	2.403151259	5.023889293
Nx5/6 (28 days)	50.13933874	1.107001797	3.400681808	5.963930548
Nx5/6 (28 days)	51.12579781	1.146151353	3.467588025	6.174847308

Correction LC3B-II				
Group	LC3B-II-2/β-Actin LC3B-II-2	LC3B-II-1/β-Actin LC3B-II-1	Correction 1	Correction 2
Control	20.27475539	0.017504634	1	1.000000003
Control	20.28229529	0.017396696	1.000371886	0.993833741
Nx5/6 (2 days)	20.74547194	0.024578027	1.023216879	1.404086905
Nx5/6 (2 days)	21.26737481	0.024569282	1.048958392	1.403587306
Nx5/6 (4 days)	22.45760298	0.032762704	1.107663326	1.87165888
Nx5/6 (4 days)	23.04178188	0.032509191	1.136476443	1.857176278
Nx5/6 (7 days)	26.60565435	0.036629332	1.312255257	2.092550575
Nx5/6 (7 days)	27.75634106	0.037237552	1.36900991	2.127296813
Nx5/6 (14 days)	29.18365687	0.041433902	1.43940858	2.367024736
Nx5/6 (14 days)	29.50121341	0.041806892	1.455071237	2.388332806
Nx5/6 (28 days)	31.77157327	0.059160627	1.56705088	3.379712325
Nx5/6 (28 days)	31.07929096	0.060324574	1.532905841	3.44620598

LC3B-II/LC3B-I Ratio (Figure 5)

	LC3B-II		LC3B-I		Ratio	
Group	Correction 1	Correction 2	Correction 1	Correction 2	LC3B-II/LC3B-I	
Control	1	1	1.00620452	1	0.99383374	1
Control	1.00037189	0.99383374	1	1.22267675	1.00037189	0.81283441
Nx5/6 (2 days)	1.02321688	1.4040869	1.41279859	1.10023321	0.72424823	1.27617209
Nx5/6 (2 days)	1.04895839	1.40358731	1.41229589	0.97366188	0.74273274	1.44155517
Nx5/6 (4 days)	1.10766333	1.87165888	1.88327162	3.04064443	0.58815909	0.61554678
Nx5/6 (4 days)	1.13647644	1.85717628	1.86869916	3.57593375	0.60816447	0.51935422
Nx5/6 (7 days)	1.31225526	2.09255057	2.10553384	4.4523722	0.62324111	0.46998554
Nx5/6 (7 days)	1.36900991	2.12729681	2.14049566	4.10627332	0.63957612	0.51806021
Nx5/6 (14 days	1.43940858	2.36702474	2.38171098	5.06530424	0.60435905	0.46730159
Nx5/6 (14 days	1.45507124	2.38833281	2.40315126	5.02388929	0.60548467	0.47539519
Nx5/6 (28 days	1.56705088	3.37971232	3.40068181	5.96393055	0.46080491	0.5666921
Nx5/6 (28 days	1.53290584	3.44620598	3.46758802	6.17484731	0.44206689	0.55810384
